# Supplementary material for: Comparative Analysis of Cyclization Techniques in Stapled Peptides: Structural Insights into Protein–Protein Interactions in a SARS-CoV-2 Spike RBD/hACE2 Model System
Source: Int J Mol Sci. 2023 Dec 21;25(1):166. doi: 10.3390/ijms25010166 (PMC10778704; doi:10.3390/ijms25010166)

Supplementary information

Comparative Analysis of Cyclization Techniques in Stapled Peptides: Structural Insights into Protein-Protein Interactions in a SARS-CoV-2 Spike RBD/hACE2 Model System

Sára Ferková [1], Ulrike Froehlich [1], Marie-Édith Nepveu-Traversy [1], Alexandre Murza [1], Taha Azad [1], Michel Grandbois [1], Philippe Sarret [1], Pierre Lavigne [1] and Pierre-Luc Boudreault [1,*]

Department of Pharmacology and Physiology, Faculty of Medicine and Health Sciences, Institut de Pharmacologie de Sherbrooke, Université de Sherbrooke, 3001 12e Avenue Nord, Sherbrooke, QC J1H 5N4, Canada

* E-mail : Pierre-Luc.Boudreault@USherbrooke.ca

Contents

1. Supplementary figures 2
2. Supplementary tables.. 11
3. Peptide characterization tables and UPLC-MS spectra.. 16
4. 1H Nuclear magnetic resonance (NMR) spectra 43

a. ^{1}H NMR of **4** (600 MHz, tampon phosphate, pH 6.8, 40nM KPO4, 40 KCl). 43

b. ^{1}H NMR of **9** (600 MHz, tampon phosphate, pH 6.8, 40nM KPO4, 40 KCl). 43

c. ^{1}H NMR of **10** (600 MHz, tampon phosphate, pH 6.8, 40nM KPO4, 40 KCl). 44

d. ^{1}H NMR of **11** (600 MHz, tampon phosphate, pH 6.8, 40nM KPO4, 40 KCl). 44

Supplementary information

1. Supplementary figures

A **B**

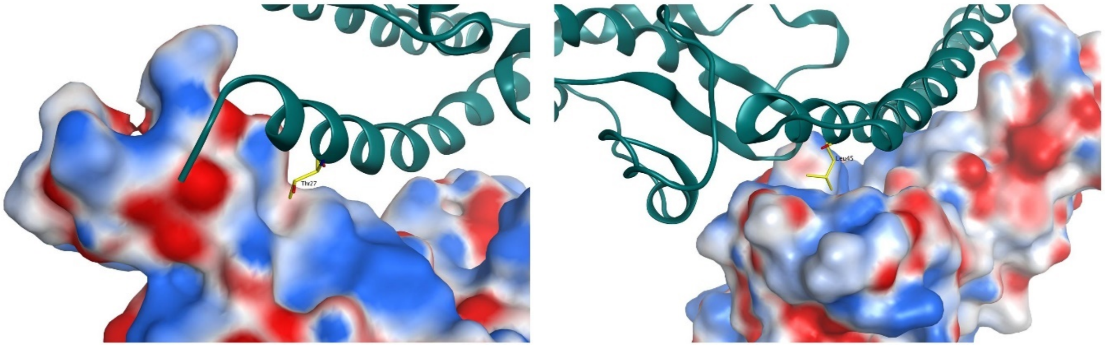

Figure S1. Representation of (**A**) Thr27 and (**B**) Leu45 hACE2 residues interacting with the hydrophobic binding pocket of SARS-CoV-2 RBD, showed with the electrostatic potential interpolated at its molecular surface. Thr27 of ACE2 pointing inside a small binding pocket of SARS-CoV-2 RBD. Positive and negative electrostatic potential are colored in blue and red, respectively.

A

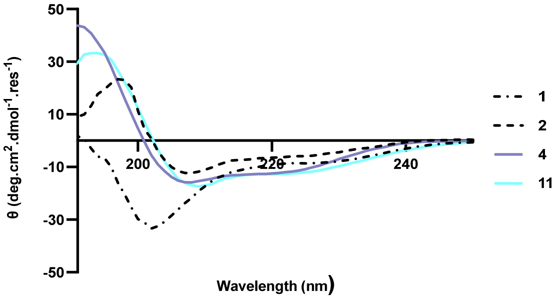

B

Name	Peptide sequence	Macrocyclization			Helicity in PBS (%)	Helicity in 50%TFE (%)
		Type	Position	Amino acid		
1	Ac-NH-Asp-Lys-Phe-Asn-His-Glu-Ala-Glu-Asp-CO-NH2	-	-	-	5.3 (0.15)[b]	36.9
2	Ac-NH-His-Glu-Ala-Glu-Asp-Leu-Phe-Tyr-Gln-CO-NH2	-	-	-	4.7 (0.35)[b]	27.3
4	Ac-NH-His-Glu-[Lys-Glu-Asp-Leu-Asp]-Tyr-Gln-CO-NH2	Lactam	i, i+4	Lys---Asp	55.5	49.4
11	Ac-NH-His-Glu-[S5-Glu-Asp-Leu-S5]-Tyr-Gln-CO-NH2	RCM	i, i+4	S5---S5	53.5	52.1

Figure S2. (A) CD data for hACE2-derived peptides measured at a concentration of 100 μM in a 50/50 mixture of TFE/10 mM sodium phosphate buffer at pH = 7.4 and 25°C. **(B)** Table overviewing derivatives **1**, **2**, **4** and **11** with focus on macrocyclization technique and measure-based calculated helicity (%) via CD. Percent helicity was calculated at 222nm as indicated in the quantification of helicity section of Supplementary information.

A

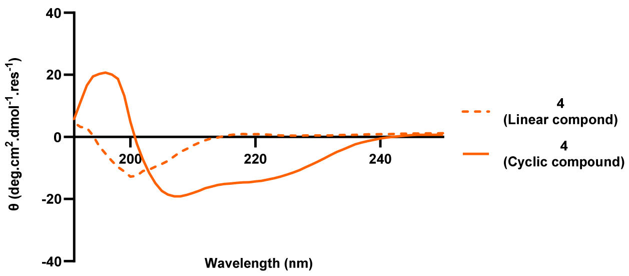

B

Name	Peptide sequence	Macrocyclization			Structure
		Type	Position	Amino acid	
4 (Linear compound)	Ac-NH-His-Glu-[Lys-Glu-Asp-Leu-Asp]-Tyr-Gln-CO-NH2	-	-	-	Random coil
4 (Cyclic compound)	Ac-NH-His-Glu-[Lys-Glu-Asp-Leu-Asp]-Tyr-Gln-CO-NH2	Lactam	i, i+4	Lys---Asp	55.5

Figure S3. (A) Comparison of the Far-UV spectra of linear **4** and lactam stapled **4** measured at concentration of 100 µM in 10 mM sodium phosphate buffer at pH = 7.4 and 25°C. **(B)** Table summarizing linear **4** and lactam stapled **4** estimated helicity (%) as described in the materials and methods of the manuscript.

A

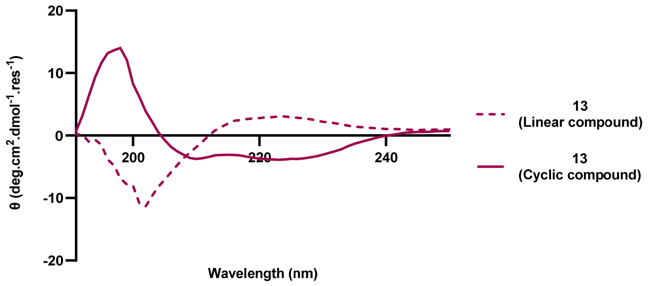

B

Name	Peptide sequence	Macrocyclization			Structure
		Type	Position	Amino acid	
13 (Linear compound)	Ac-NH-His-Glu-Lys-Glu-Asp-Leu-dPra-Tyr-Gln-CO-NH2	-	-	-	Random coil
13 (Cyclic compound)	Ac-NH-His-Glu-[Lys-Glu-Asp-Leu-dPra]-Tyr-Gln-CO-NH2	Triazole	i, i+4	Lys(N_3)---dPra	18.1

Figure S4. (A) Comparison of the Far-UV spectra of linear **13** and triazole stapled **13** measured at concentration of 100 µM in 10 mM sodium phosphate buffer at pH = 7.4 and 25°C. **(B)** Table summarizing linear **13** and lactam stapled **13** estimated helicity (%) as described in the materials and methods of the manuscript.

A

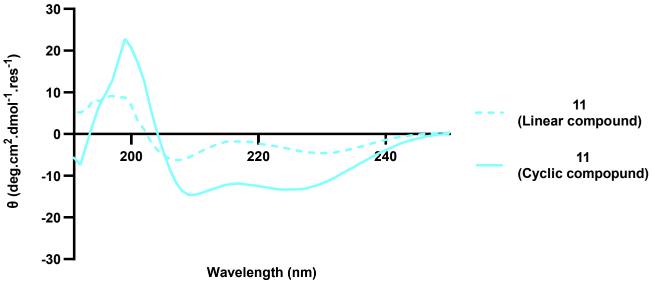

B

Name	Peptide sequence	Macrocyclization			Helicity in PBS (%)
		Type	Position	Amino acid	
11 (Linear compound)	Ac-NH-His-Glu-S5-Glu-Asp-Leu-S5-Tyr-Gln-CO-NH2	-	-	-	11.0
11 (Cyclic compound)	Ac-NH-His-Glu-[S5-Glu-Asp-Leu-S5]-Tyr-Gln-CO-NH2	RCM	i, i+4	S5---S5	53.5

Figure S5. (A) Comparison of the Far-UV spectra of linear **11** and hydrocarbon stapled **11** measured at concentration of 100 μM in 10 mM sodium phosphate buffer at pH = 7.4 and 25°C. **(B)** Table summarizing linear **11** and lactam stapled **11** estimated helicity (%) as described in the materials and methods of the manuscript.

A

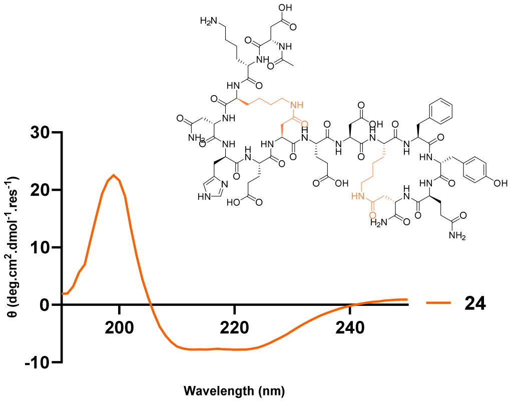

B

Name	Peptide sequence	Macrocyclization			Helicity in PBS (%)
		Type	Position	Amino acid	
24	Ac-NH-Asp-Lys-[Lys-Asn-His-Glu-Asp]-Glu-Asp-[Lys-Phe-Tyr-Gln-Asp]-CO-NH2	Double Lactam	i. i+4 and i, i+4	Lys---Asp and Lys---Asp	25.2

Figure S6. (A) The Far-UV spectra of double lactam stapled peptide **24** measured at concentration of 100 μM in 10 mM sodium phosphate buffer at pH = 7.4 and 25°C. **(B)** Table summarizing double lactam stapled peptide **24** estimated helicity (%) as described in the materials and methods of the manuscript.

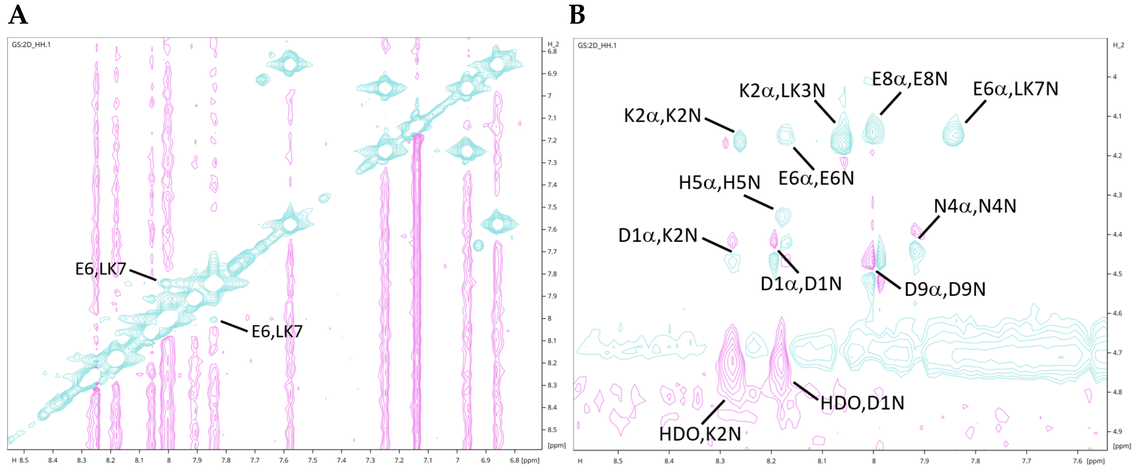

Figure S7. (**A**) The amide and (**B**) fingerprint regions of NOESY spectrum recorded for **9**.

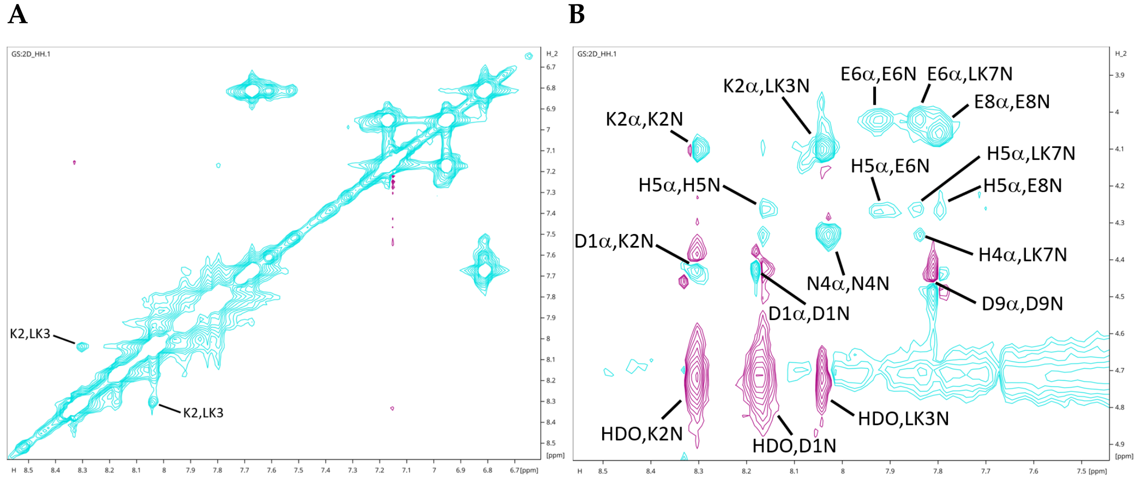

Figure S8. (**A**) The amide and (**B**) fingerprint regions of NOESY spectrum recorded for **10**.

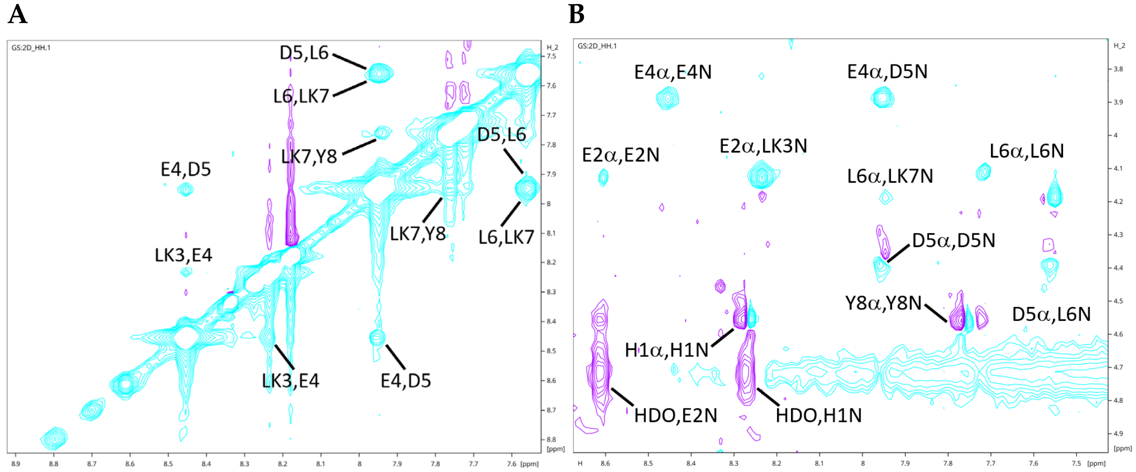

Figure S9. (**A**) The amide and (**B**) fingerprint regions of NOESY spectrum recorded for **11**.

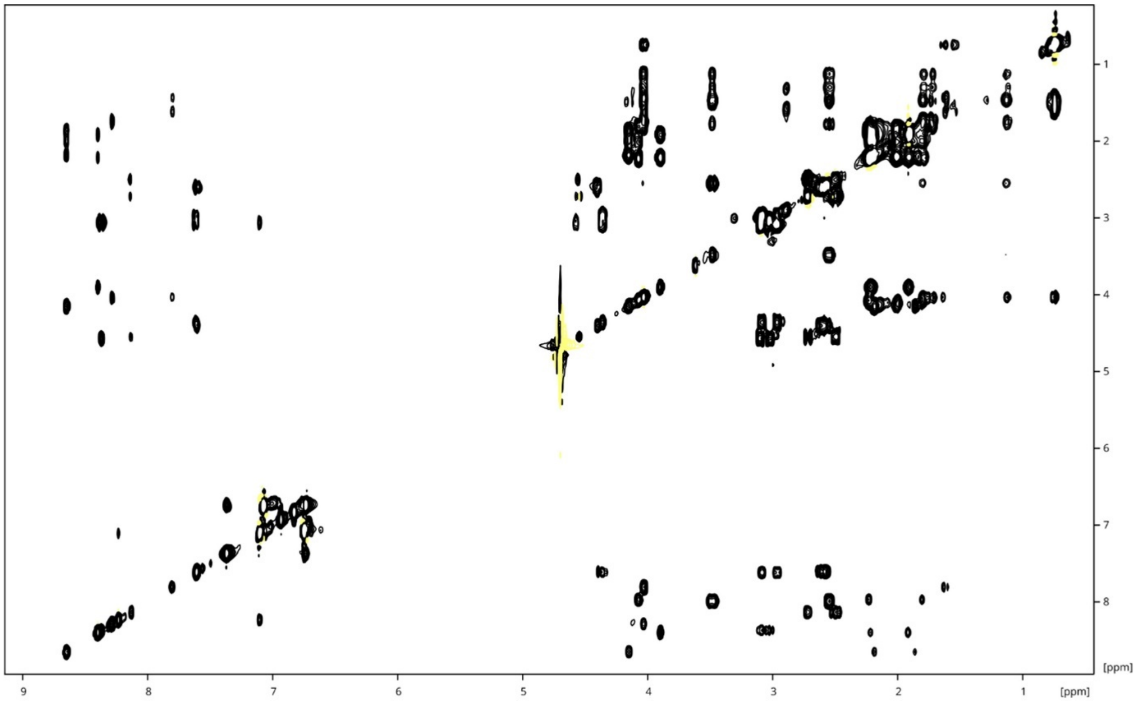

Figure S10. TOCSY spectrum recorded for **4**.

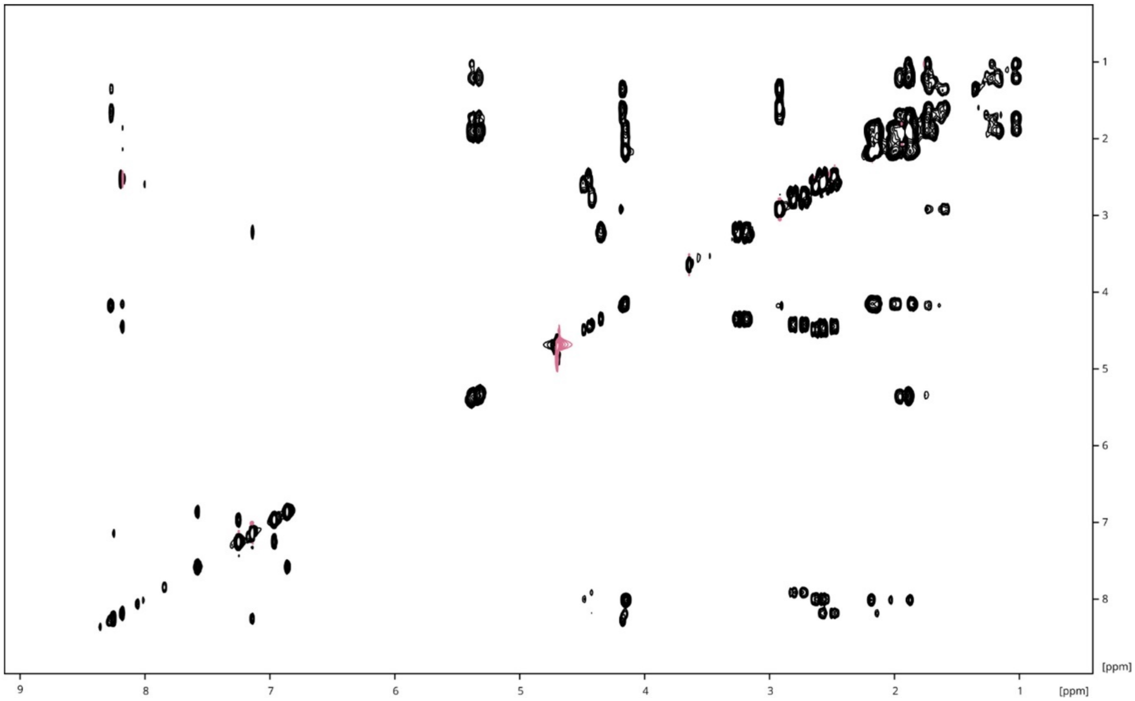

Figure S11. TOCSY spectrum recorded for **9**.

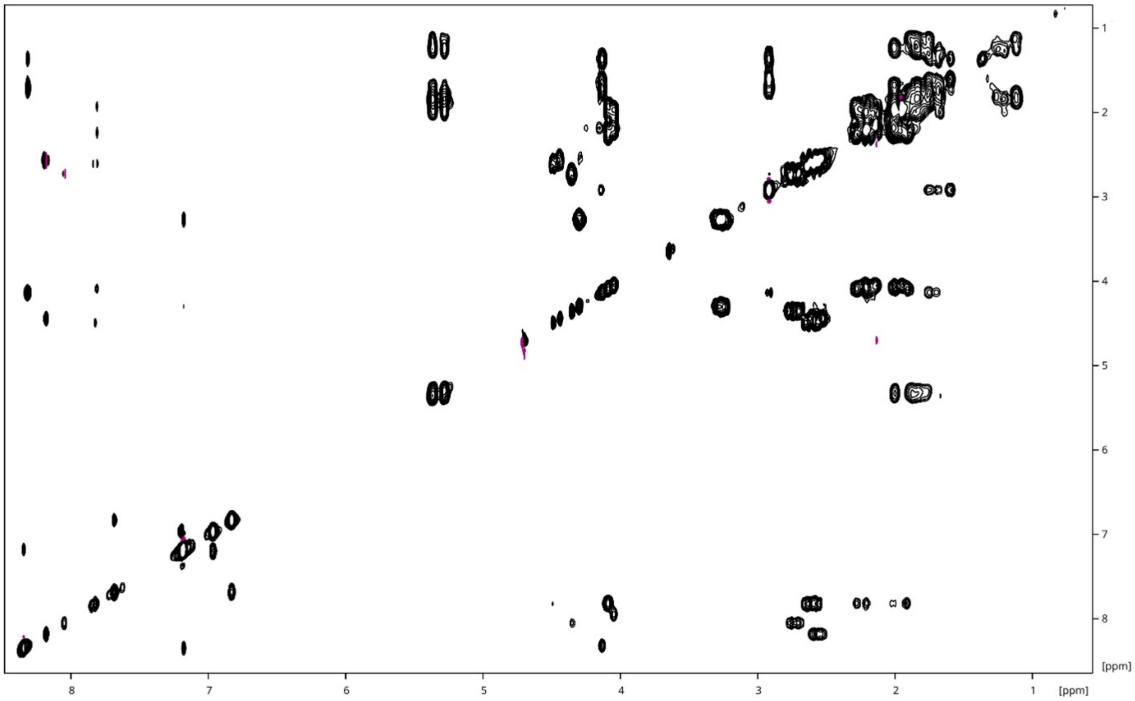

Figure S12. TOCSY spectrum recorded for **10**.

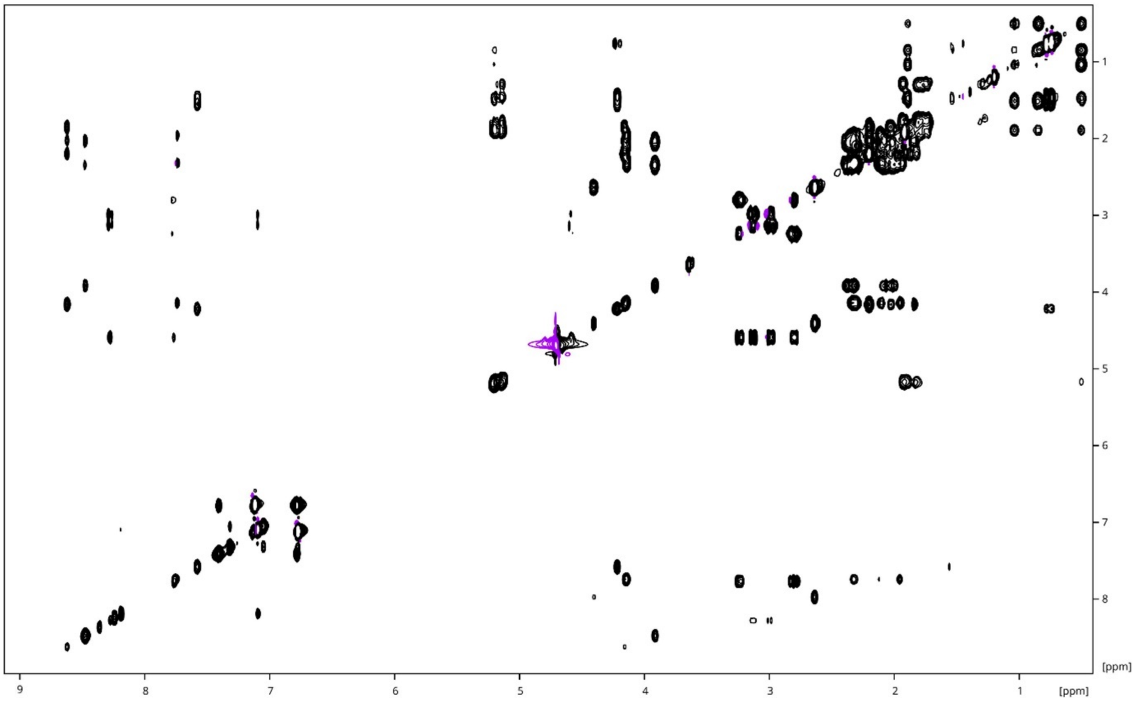

Figure S13. TOCSY spectrum recorded for **11**.

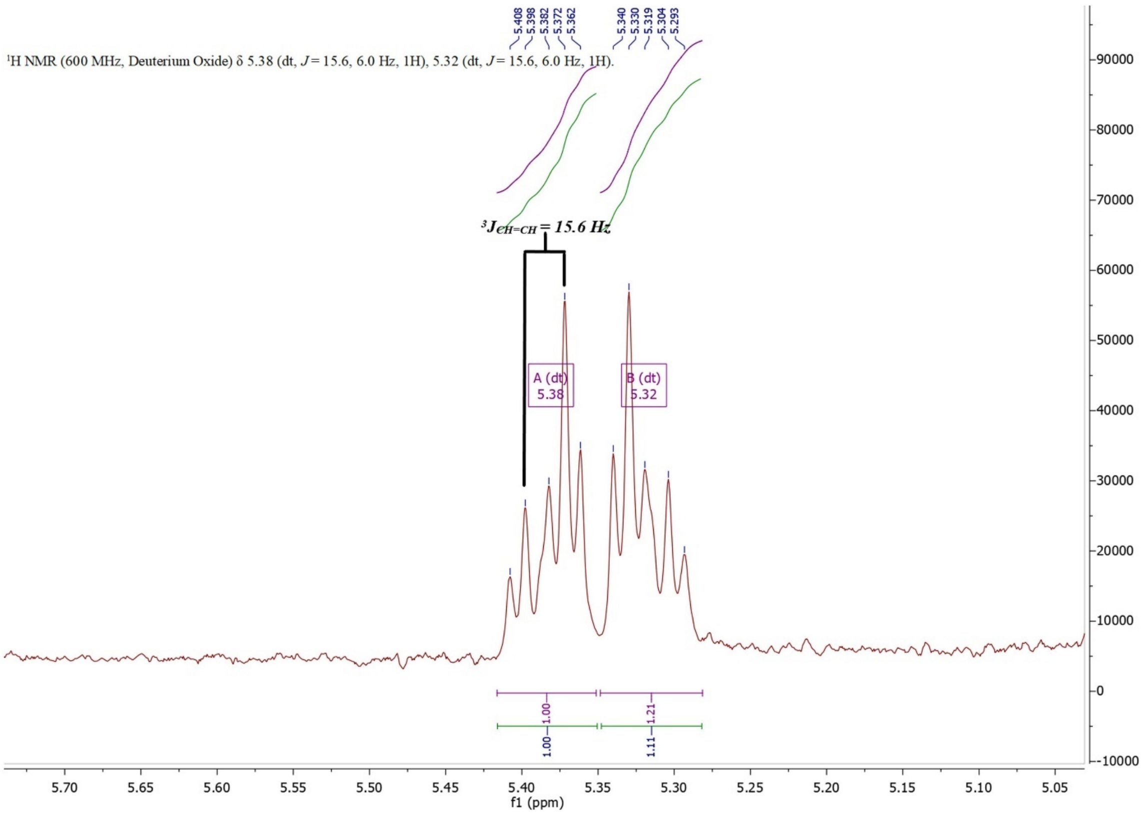

Figure S14. ^{1}H NMR Double bond isomer region spectra of **9**.

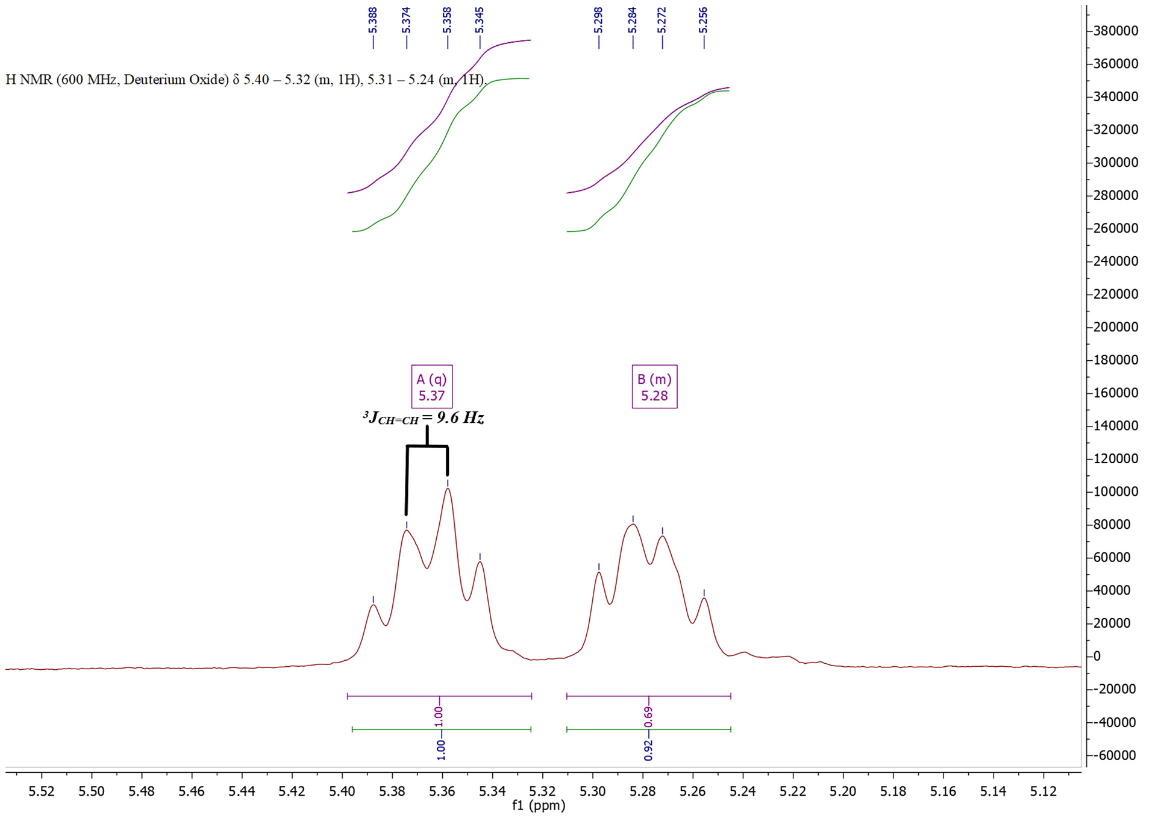

Figure S15. ^{1}H NMR Double bond isomer region spectra of **10**.

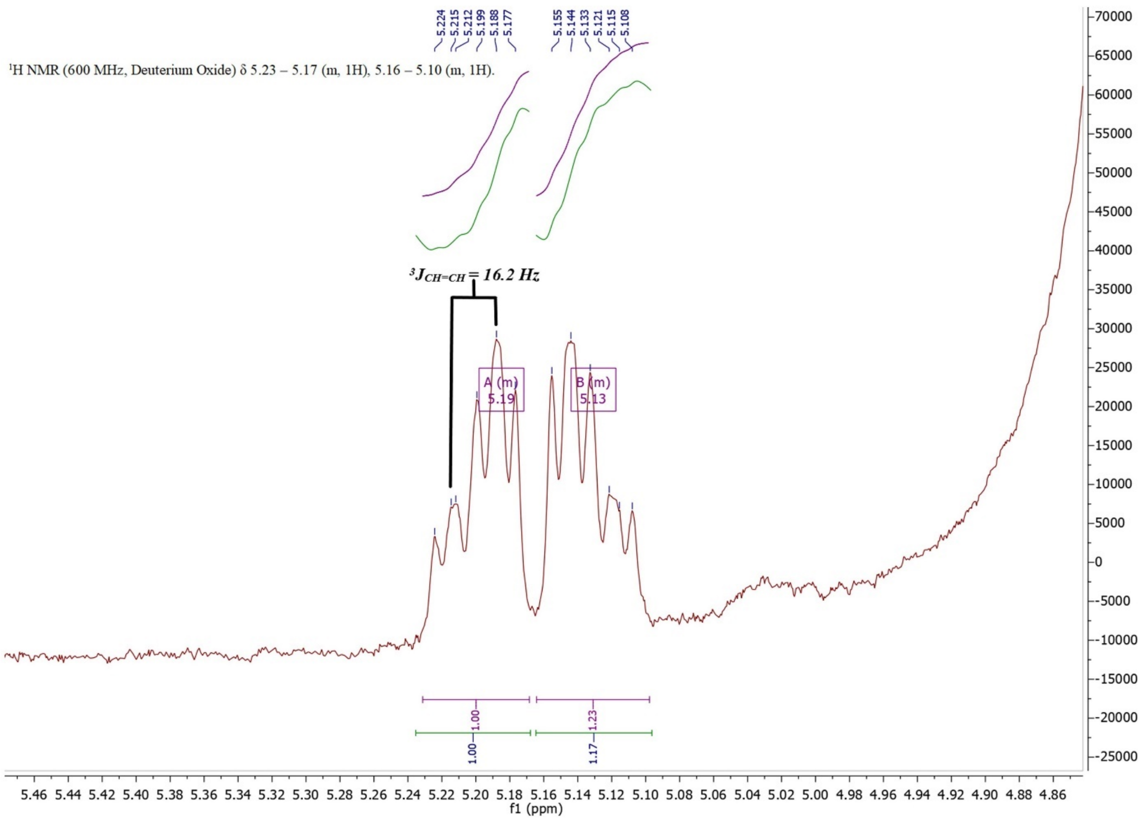

Figure S16. ^{1}H NMR Double bond isomer region spectra of **11**.

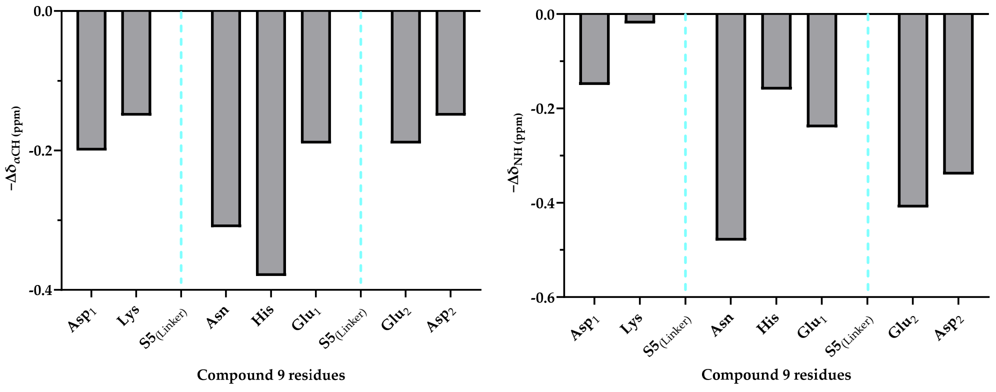

A B

Figure S17. Chemical shift information of CαH (**A**) and NH (**B**) protons compared to a characteristic random coil shift[a] for the peptide **9**. [a] Random-coil chemical shifts for 20 common amino acids followed by alanine were measured using a peptide with free N- and C-termini at pH 5.0 and 25 °C.

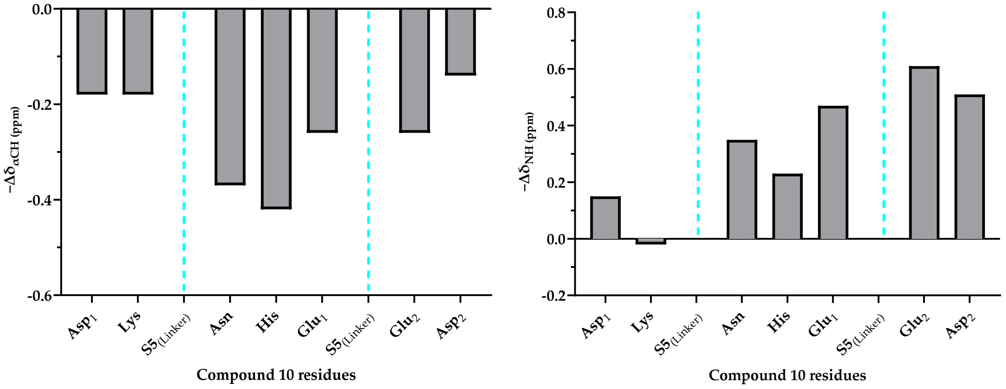

A B

Figure S18. Chemical shift information of CαH (**A**) and NH (**B**) protons compared to a characteristic random coil shift[a] for the peptide **10**. [a] Random-coil chemical shifts for 20 common amino acids followed by alanine were measured using a peptide with free N- and C-termini at pH 5.0 and 25 °C.

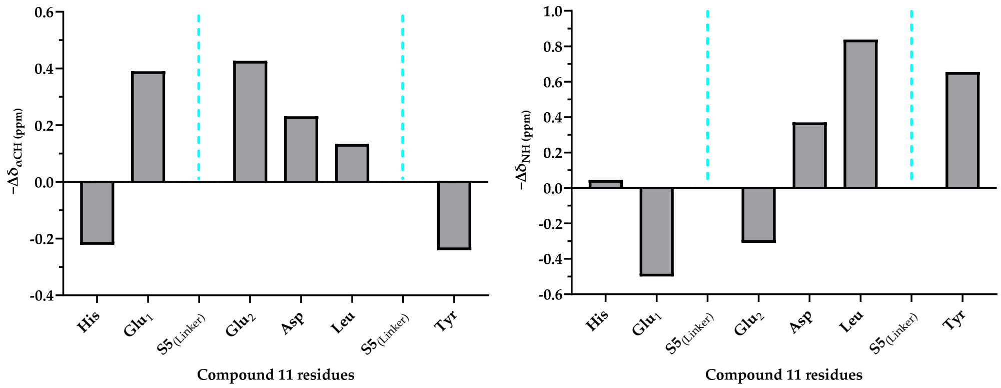

A B

Figure S19. Chemical shift information of CαH (**A**) and NH (**B**) protons compared to a characteristic random coil shift[a] for the peptide **11**. [a] Random-coil chemical shifts for 20 common amino acids followed by alanine were measured using a peptide with free N- and C-termini at pH 5.0 and 25 °C.

2. Supplementary tables

Table S1. Contact surface residues involved in hydrogen bonding (H-bond) network between SARS-CoV-2 S RBD protein and ACE2 protein.

**ACE2**	**SARS-COV-2 (Hydrogen bond forming atoms, distance in Å)**			
	**PDB 6M0J**	**PDB 6VW1**	**PDB 6LZG**	**PDB 6M17**
Ser19	-	Ala475 (HG-O, 1,74)	Ala475 (HG-O, 1.87)	-
Gln24	Asn487 (OE1-HD21, 1.88)	Asn487 (OE1-HD21, 2.22)	Asn487 (OE1-HD21, 2.13)	-
Thr27				Tyr489 (O-HH, 2.10)
Asp30	Lys417 (OD2-HZ3, 1.77)	-	Lys417 (OD1-HZ3, 2.08)	Lys417 (OD1, HE2, 2.28) (OD1- HZ3, 2.30) (OD2-HZ3, 1,74)
Lys31	-	Gln493 (HZ1-OE1, 1.98)	-	-
His34	-	-	-	-
Glu35	-	Gln493 (OE2-HE21, 1.97)	Gln493 (OE1-HE21, 2.50)	-
Glu37	Tyr505 (OE2-HH, 2.50)	Tyr505 (OE1-HH, 2.21)	-	-
Asp38	Tyr449 (OD2-HH, 2.94)	Tyr449 (OD2-HH, 2.45) (OD1-HH, 2.07)	Tyr449 (OD1-HH, 1.88)	Tyr449 (OD1-HH, 2.18) (OD2-HH, 2.59)
Tyr41	-	-	-	Asn501 (OD1-HH, 2.34)
Gln42	Gly446 (HE21-O, 2.34)	Gln498 (HE21-OE1, 2.14)	Gln498 (OE1-HE22, 1.64)	Gln498 (OE1-HE22, 1.92)
	Tyr449 (HE22-OH, 2.02)	-	-	-
Tyr83	Asn487 (HH-OD1, 1.87)	Asn487 (HH-OD1, 1.99)	Asn487 (HH-OD1, 1.88)	Asn487 (HH-OD1, 2.14)
Lys353	Gly496 (HZ2-O, 2.27)	Gly496 (HZ2-O, 2.04)	Gly496 (HZ1-O, 2.39)	Gly496 (HZ3-O, 2.27)
	Gly502 (O-H, 1.79)	Gly502 (O-H, 1.86)	Gly502 (O-H, 1.80)	-
Gly354	-	-	-	Gly502 (HA3-O, 2.10)
Arg357	-	-	-	Thr500 (HH12-OG1, 1.76)

Table S2. Contact surface residues involved in salt bridge network formation between SARS-CoV-2 S RBD protein and ACE2 protein.

ACE2	SARS-CoV-2 (Salt bridges, distance in Å)			
	**PDB 6M0J**	**PDB 6VW1**	**PDB 6LZG**	**PDB 6M17**
Asp30	Lys417<br>(OD2-NZ, 2.76)	-	Lys417<br>(OD1-NZ, 2.74)<br>(OD2-NZ, 3.76)	Lys417<br>(OD1-NZ, 3.01)<br>(OD2-NZ, 2.68)

Table S3. Contact surface residues involved in Hydrophobic network formation between SARS-CoV-2 S RBD protein and ACE2 protein. (PDB 6M0J)

ACE2	SARS-CoV-2	Hydrophobic contacts (distance in Å)
Leu79		CD2-CD1 (4.16)
Met82	Phe486	CB-CE1 (3.63)
Tyr83		CE1-CE1 (3.79)
	Leu455	CG-CG2 (7.47)
	Phe456	CG2-CZ (3.93)
Thr27	Tyr473	CG2-CE2 (4.27)
	Ala475	CG2-CB (3.95)
Lys353	Tyr505	CG-CD2 (3.75)

Table S4. Main-chain hydrogen bonds stabilizing the α1-helix of hACE2. (PDB 6M0J)

ACE2	ACE2	Hydrogen bond (distance in Å)
Asp38	Lys353	OD1-NZ (2.9)
Tyr41	Asp355	OD1-HH (1.79)
Thr20	Gln24	N-O (3.31)
Ile21	Ala25	O-N (2.98)
Glu22	Lys26	O-N (2.88)
Ala23	Thr27	O-N (3.16)
Gln24	Phe28	O-N (2.76)
Ala25	Leu29	O-N (2.8)
Lys26	Asp30	O-N (2.9)
Thr27	Lys31	O-N (3.02)
Phe28	Phe32	O-N (2.84)
Leu29	Asn33	O-N (2.82)
Asp30	His34	O-N (3.59)
Lys31	Glu35	O-N (3.1)
Phe32	Ala36	O-N (2.64)
Asn33	Glu37	O-N (3.03)
His34	Asp38	O-N (3.63)
Glu35	Leu39	O-N (3.11)
Ala36	Phe40	O-N (2.69)
Glu37	Tyr41	O-N (3.00)
Asp38	Gln42	O-N (3.01)
Leu39	Ser43	O-N (3.21)
Phe40	Ser44	O-N (2.90)

Table S5. ^{1}H NMR assignments for **9**.

Residue	NH	CαH	CβH	CγH	CδH	CεH
N-terminus						
Asp	8.19	4.44	2.50, 2.58	-	-	-
Lys	8.27	4.17	1.72,1.74	1.60, 1.65	*, *	2.92, *
S5 (linker)	8.06	-	*, *	*, *	*, *	*
Asn	7.92	4.43	2.73, 2.81	-	*, *	-
His	8.26	4.35	3.19, 3.26	-	*, *	*, *
Glu	8.18	4.16	1.86, 199	2.14	-	-
S5 (linker)	7.84	-	*, *	*, *	*, *	*
Glu	8.01	4.16	1.88, 2.02	2.19	-	-
Asp	8.00	4.49	2.57, 2.64	-	-	-
C-terminus						

** The protons could not be assigned due to rapid chemical exchange or other proton shift superposition.*

Table S6. ^{1}H NMR assignments for **10**.

Residue	NH	CαH	CβH	CγH	CδH	CεH
N-terminus						
Asp	8.19	4.46	2.55, 2.60	-	-	-
Lys	8.31	4.14	1.68, 1.75	1.35, *	*, *	2.92
S5 (linker)	8.04	-	*, *	*, *	*, *	*
Asn	8.05	4.37	2.70, 2.77	-	*, *	-
His	8.19	4.31	3.26, *	-	*, *	*, *
Glu	7.95		1.901, 2.10	2.21, 2.28	-	-
S5 (linker)	7.89	-	*, *	*, *	*, *	*
Glu	7.81	4.09	1.92, 2.02	2.22, 2.28	-	-
Asp	7.83	4.50	2.58, 2.64	-	-	-
C-terminus						

** The protons could not be assigned due to rapid chemical exchange or other proton shift superposition.*

Table S7. ^{1}H NMR assignments for **11**.

Residue	NH	CαH	CβH	CγH	CδH	CεH
N-terminus						
His	8.275	4.562	2.985, 3.114	-	*, *	*, *
Glu	8.62<br>8.70	4.16<br>4.24	1.85, 2.03<br>1.84, 2.12	2.20, *<br>2.24, *	-	-
S5 (linker)	8.23	-	*, *	*, *	*, *	*, *
Glu	8.47	3.913	2.068, 2.093	2.325, 2.374	-	-
Asp	7.97	4.408	2.636, *	-	-	-
Leu	7.582<br>7.40	4.216<br>4.30	1.464	1.56 and<br>1.531	0.765	-
S5 (linker)	7.96	-	*, *	*, *	*, *	*, *
Tyr	7.766	4.591	2.80, 3.24	-	*, *	3.242, *
Gln	*	*	*, *	*, *	-	*, *
C-terminus						

** The protons could not be assigned due to rapid chemical exchange or other proton shift superposition.*

Table S8. Schematic representation of NOESY patterns involving NH and CαH protons observed in NOESY spectrum recorded for α-helix compared to **11**. Horizontal lines of various lengths indicate NOE connectivities between protons of peptide sequences. The thickness of solid lines is proportional to the observed strong, medium, and weak NOEs signal intensities.

	Theoretical		**11**
	3.6_{13}-helix	3_{10}-helix	
$d_{NN(i,i+1)}$	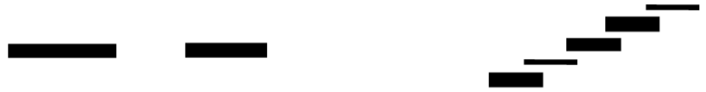		
$d_{\alpha N(i+1)}$	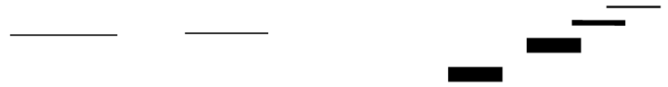		
$^3J_{\alpha NH}$	4 4 4 4 4 4 4 4	4 4 4 4 4 4	7.8 5.4 - 3.0 5.4 6.6 3.6 7.8 -
	1 2 3 4 5 6 7	1 2 3 4 5 6	Ac-NH-His-Glu-[S5-Glu-Asp-Leu-S5]-Tyr-Gln-CO-NH_2

3. Peptide characterization tables and UPLC-MS spectra

Entry	Name	Sequence	Formula	Mass (Da)			Purity %
				Calculated	UPLC-MS $[M+H]^+$	HRMS $[M+2H]^{2+}$	
**1**	SF03-01	Ac-NH-Asp-Lys-Phe-Asn-His-Glu-Ala-Glu-Asp-CO-NH2	C48H68N14O19	1144.48	1145.90	573.25	99
**2**	SF03-02	Ac-NH-His-Glu-Ala-Glu-Asp-Leu-Phe-Tyr-Gln-CO-NH2	C54H73N13O18	1191.52	1193.19	596.77	100

Table S9. Name, Sequence, Formula, Exact calculated mass, Observed mass and purity of linear peptides. Abbreviations: NH-*Ac* = Acetylated N-terminal, CO-NH_2 = C-terminal Amide

Entry	Name	Sequence	Formula	Mass (Da)			Purity %
				Calculated	UPLC-MS $[M+H]^+$	HRMS $[M+2H]^{2+}$	
**3**	SF03-11	Ac-NH-Asp-Lys-[Lys-Asn-His-Glu-Asp]-Glu-Asp-CO-NH2	C46H69N15O20	1151.48	1152.96	576.75	100
**5**	SF03-29	Ac-NH-Asp-Lys-[Asp-Asn-His-Glu-Lys]-Glu-Asp-CO-NH2	C46H69N15O20	1151.48	1153.33	576.75	95
**7**	SF03-16	Ac-NH-Asp-Lys-[Lys-Asn-His-Glu-Glu]-Glu-Asp-CO-NH2	C47H71N15O20	1165.50	1166.70	583.76	100
**9**	SF03-06_A	Ac-NH-Asp-Lys-[S5-Asn-His-Glu-S5]-Glu-Asp-CO-NH2	C50H76N14O19	1176.54	1178.26	589.28	100
**10**	SF03-06_B				1178.18	589.28	100
**12**	SF03-19	Ac-NH-Asp-Lys-[Lys-Asn-His-Glu-dPra]-Glu-Asp-CO-NH2	C47H69N17O19	1175.50	1177.66	588.76	100
**14**	SF03-20	Ac-NH-Asp-Lys-[dPra-Asn-His-Glu-Lys]-Glu-Asp-CO-NH2	C47H69N17O19	1175.50	1177.13	588.76	98
**16**	SF03-23	Ac-NH-Asp-Lys-[Dap-Asn-His-Glu-Dap]-Glu-Asp-CO-NH2	C48H68N20O19	1229.19	1230.22	615.26	100
**18**	SF03-60	Ac-NH-Asp-Lys-[Cys-Asn-His-Glu-Cys]-Glu-Asp-CO-NH2	C50H70N14O19S2	1235.31	1236.28	618.23	98
**20**	SF03-62	Ac-NH-Asp-Lys-[Cys-Asn-His-Glu-Cys]-Glu-Asp-CO-NH2	C48H62F4N14O19S2	1279.22	640.38	640.19	95
**22**	SF03-41	Ac-NH-Pro-Asp-Lys-[Lys-Asn-His-Glu-Asp]-Glu-Asp-CO-NH2	C51H76N16O21	1248.54	1250.39	625.28	100

Table S10. Name, Sequence, Formula, Exact calculated mass, Observed mass by UPLC-MS and HRMS, and purity of SF03-01 macrocyclic derivatives. Abbreviations: NH-*Ac* = Acetylated N-terminal, CO-*NH2* = C-terminal Amide

Entry	Name	Sequence	Formula	Mass (Da)			Purity %
				Calculated	UPLC-MS $[M+H]^+$	HRMS $[M+2H]^{2+}$	
**4**	SF03-14	Ac-NH-His-Glu-[Lys-Glu-Asp-Leu-Asp]-Tyr-Gln-CO-NH2	C52H74N14O19	1198.53	1200.33	600.27	100
**6**	SF03-30	Ac-NH-His-Glu-[Asp-Glu-Asp-Leu-Lys]-Tyr-Gln-CO-NH2	C52H74N14O19	1198.53	1200.33	600.27	98
**8**	SF03-17	Ac-NH-His-Glu-[Lys-Glu-Asp-Leu-Glu]-Tyr-Gln-CO-NH2	C53H76N14O19	1212.54	1214.21	607.28	100
**11**	SF03-08_B	Ac-NH-His-Glu-[S5-Glu-Asp-Leu-S5]-Tyr-Gln-CO-NH2	C56H81N13O18	1223.58	1225.35	612.80	96
**13**	SF03-21	Ac-NH-His-Glu-[Lys-Glu-Asp-Leu-dPra]-Tyr-Gln-CO-NH2	C53H74N16O18	1222.54	1224.27	612.28	95
**15**	SF03-22	Ac-NH-His-Glu-[dPra-Glu-Asp-Leu-Lys]-Tyr-Gln-CO-NH2	53H74N16O18	1222.54	1224.27	612.28	100
**17**	SF03-24	Ac-NH-His-Glu-[Dap-Glu-Asp-Leu-Dap]-Tyr-Gln-CO-NH2	C54H73N19O18	1275.54	1277.49	638.78	98
**19**	SF03-61	Ac-NH-His-Glu-[Cys-Glu-Asp-Leu-Cys]-Tyr-Gln-CO-NH2	C56H75N13O18S2	1282,41	1283.42	641.75	100
**21**	SF03-63	Ac-NH-His-Glu-[Cys-Glu-Asp-Leu-Cys]-Tyr-Gln-CO-NH2	C54H67F4N13O18S2	1326.32	664.00	663.71	97
**23**	SF03-40	Ac-NH-Pro-His-Glu-[Lys-Glu-Asp-Leu-Asp]-Tyr-Gln-CO-NH2	C57H81N15O20	1295.58	1297.31	648.80	100

Table S11. Name, Sequence, Formula, Exact calculated mass, Observed mass by UPLC-MS and HRMS, and purity of SF03-02 macrocyclic derivatives. Abbreviations: NH-*Ac* = Acetylated N-terminal, CO-NH_2 = C-terminal Amide

Entry	Name	Sequence	Formula	Mass (Da)			Purity %
				Calculated	UPLC/MS $[M+H]^+$	HRMS $[M+2H]^{2+}$	
**Linear 4**	SF03-14_LIN	Ac-NH-His-Glu-Lys-Glu-Asp-Leu-Asp-Tyr-Gln-CO-NH2	C52H76N14O20	1217,26	1217.81	609.28	97
**Linear 8**	SF03-07	Ac-NH-His-Glu-S5-Glu-Asp-Leu-S5-Tyr-Gln-CO-NH2	C58H85N13O18	1252,39	1252.79	626.82	98
**24**	SF03-15	Ac-NH-Asp-Lys-[Lys-Asn-His-Glu-Asp]-Glu-Asp-[Lys-Phe-Tyr-Gln-Asp]-CO-NH2	C79H110N22O28	1815,88	908.61	908.40	95

Table S12. Name, Sequence, Formula, Exact calculated mass, Observed mass by UPLC-MS and HRMS, and purity of SF03-02 macrocyclic derivatives. Abbreviations: NH-*Ac* = Acetylated N-terminal, CO-NH_2 = C-terminal Amide

SF03-01: UPLC-MS

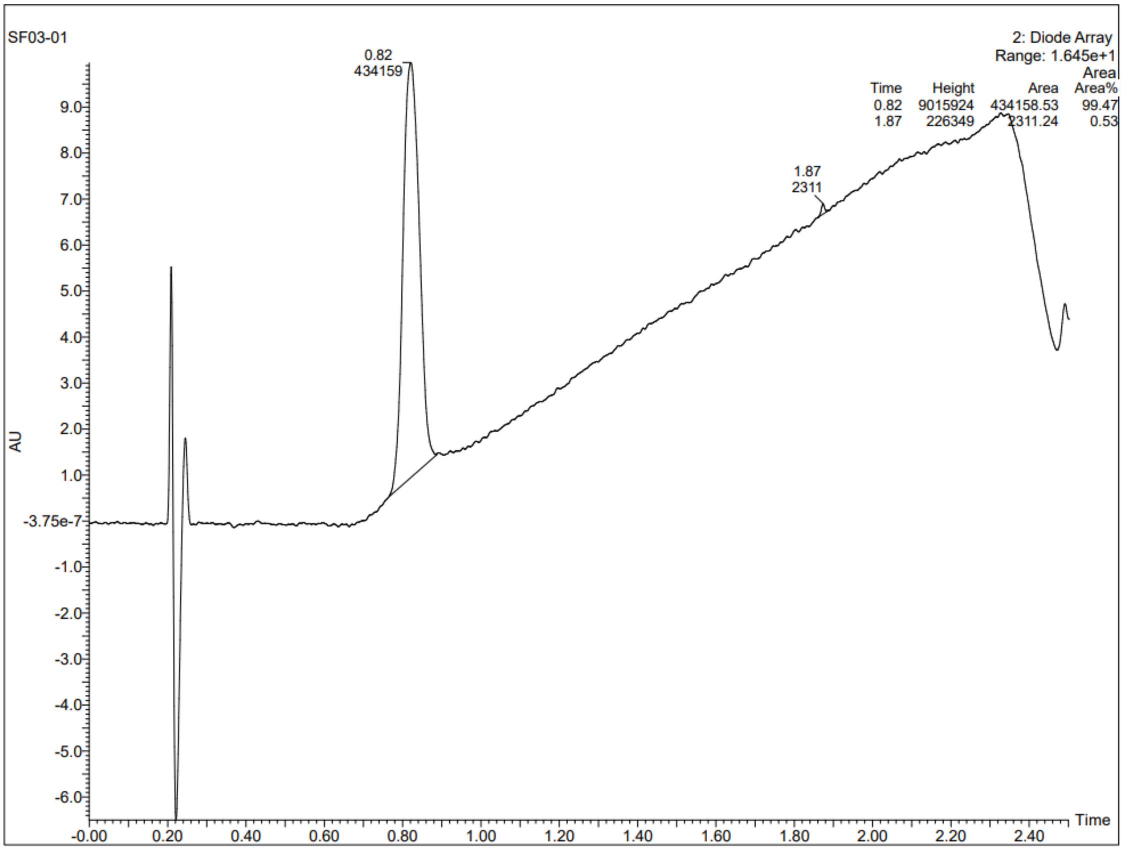

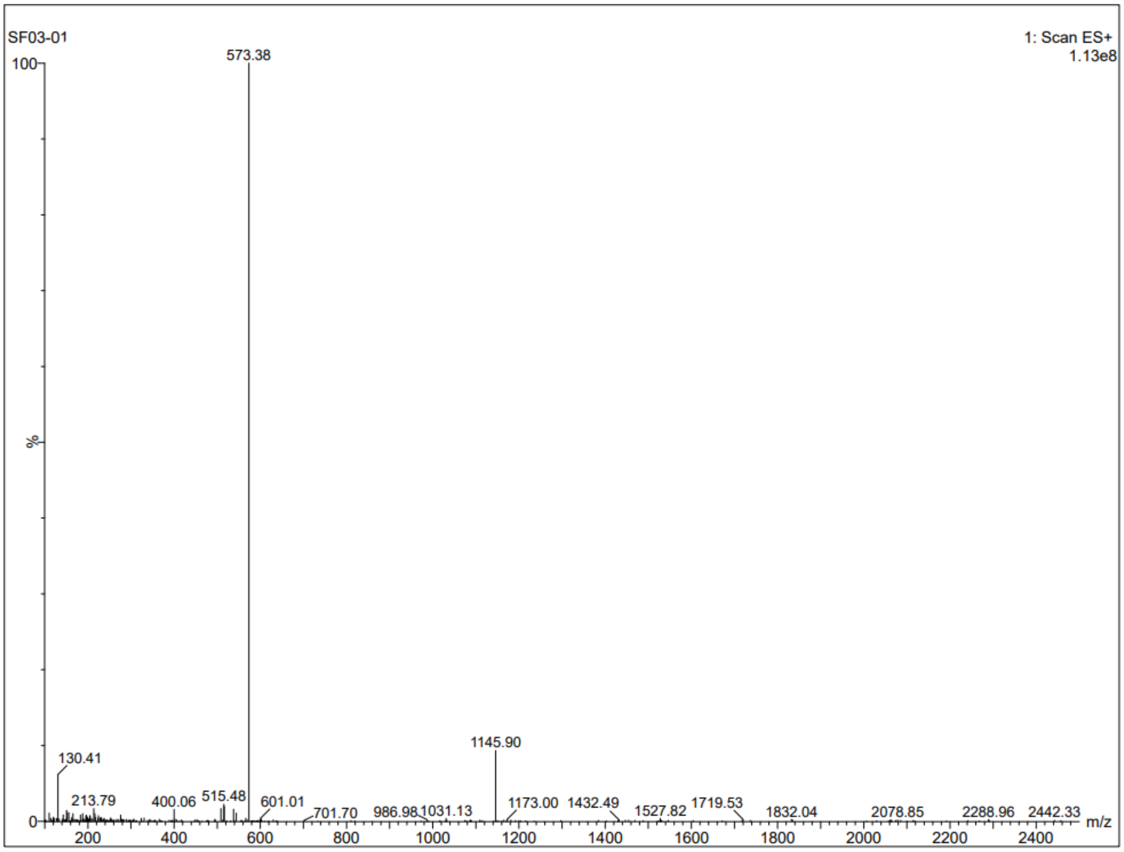

SF03-02: UPLC-MS

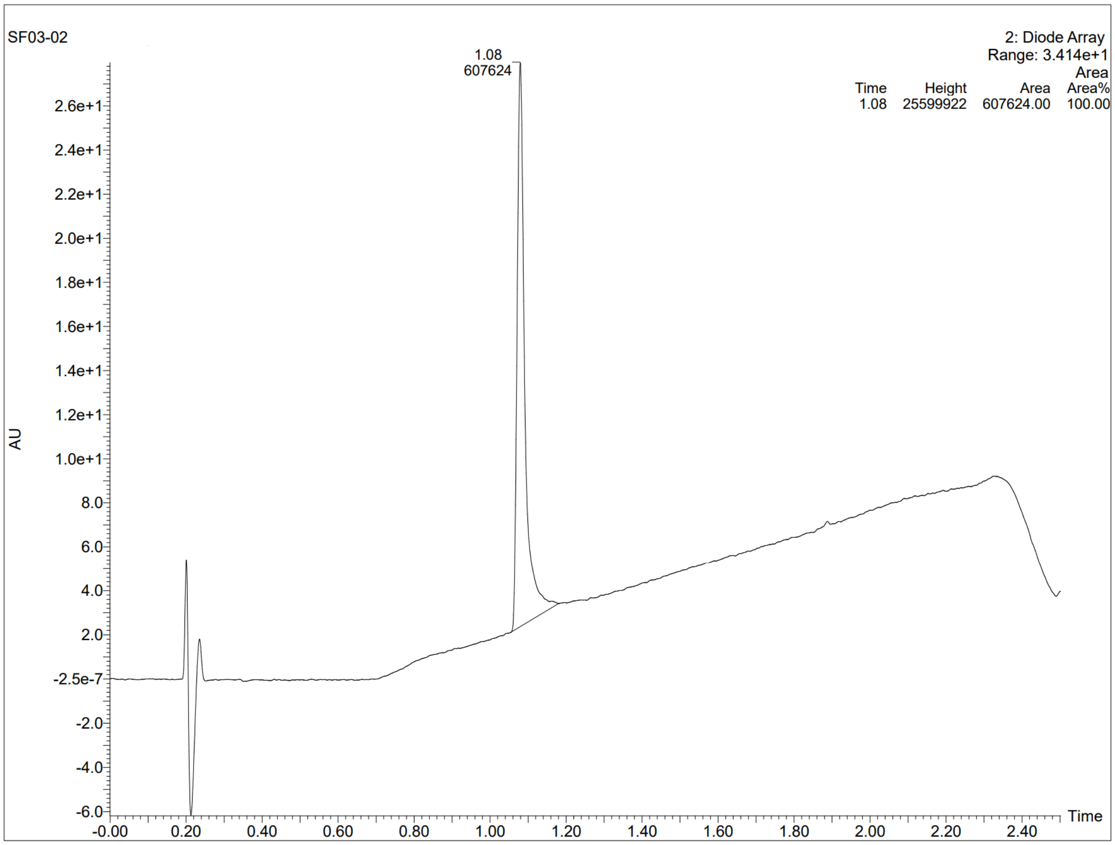

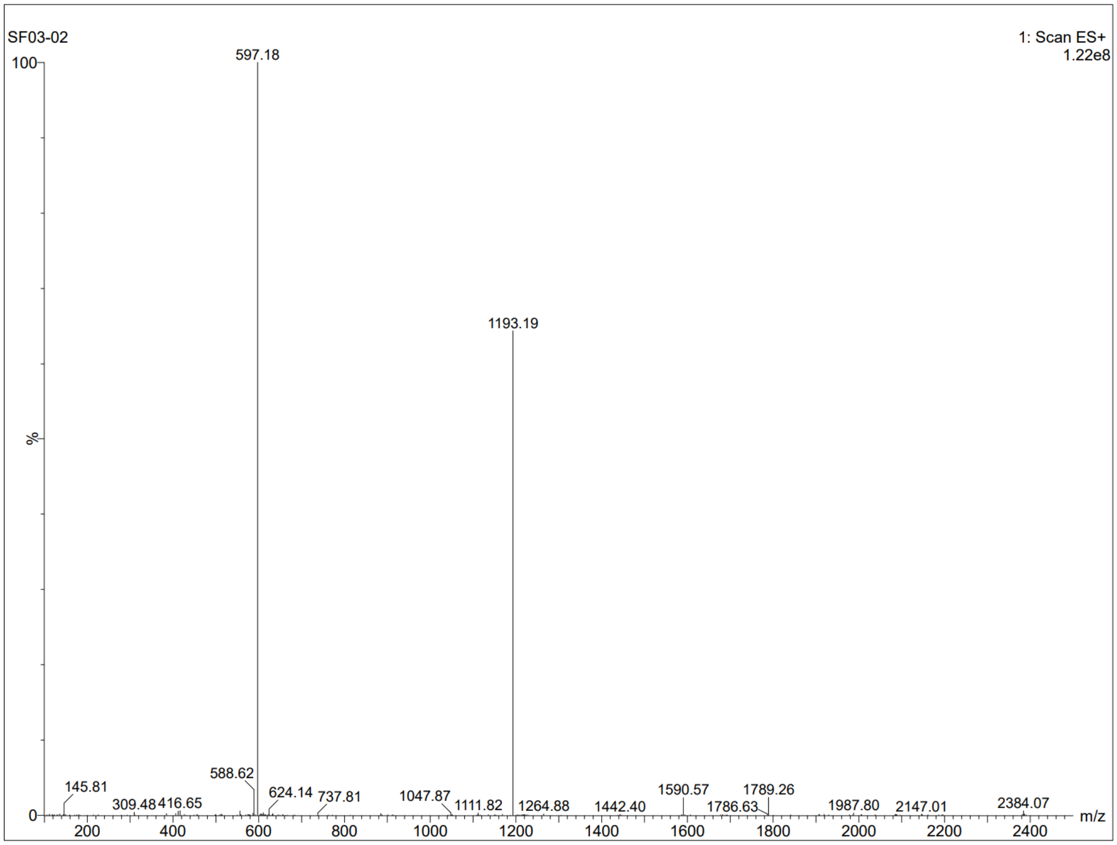

SF03-11: UPLC-MS

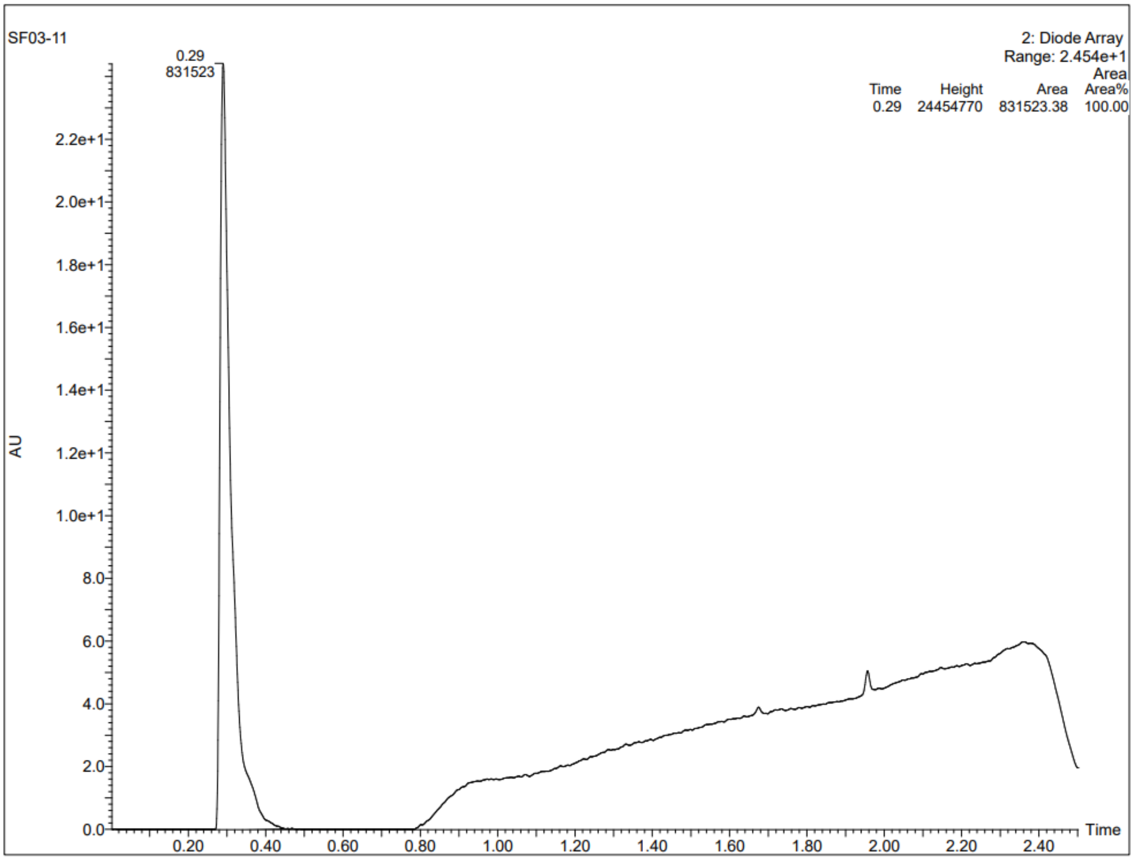

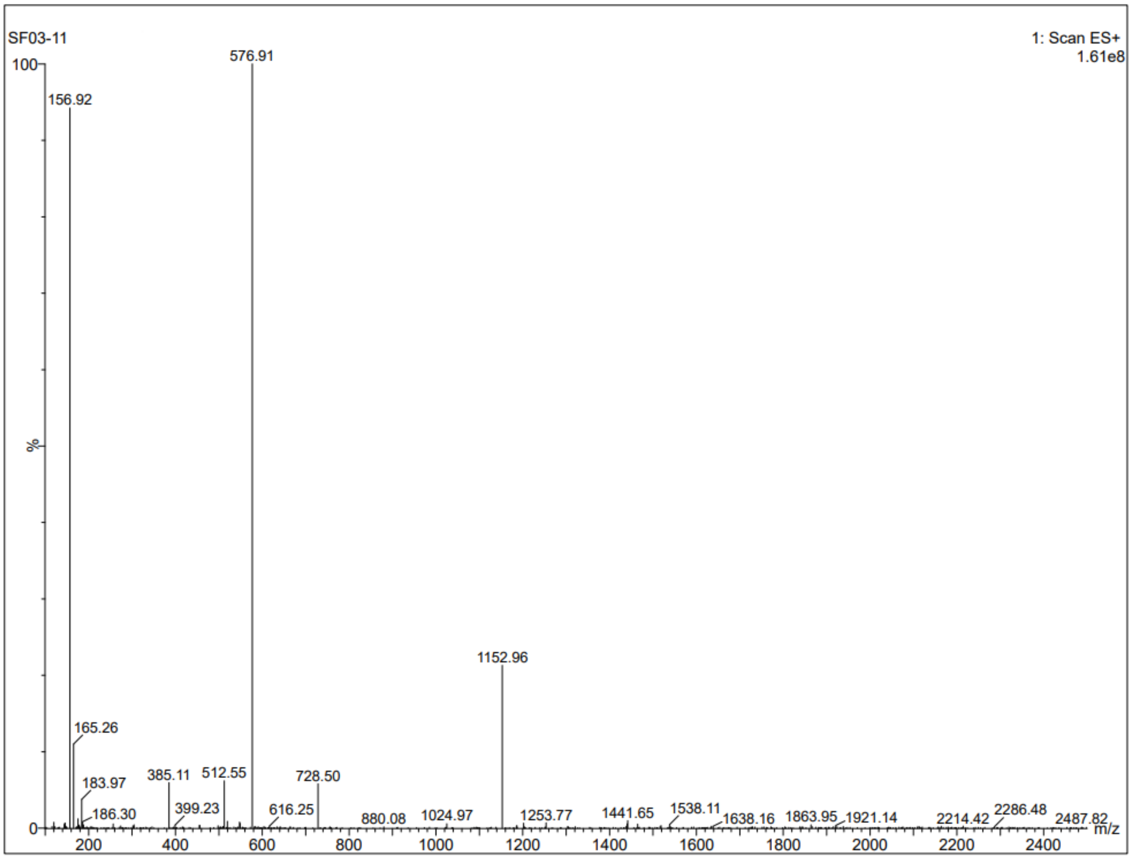

SF03-29: UPLC-MS

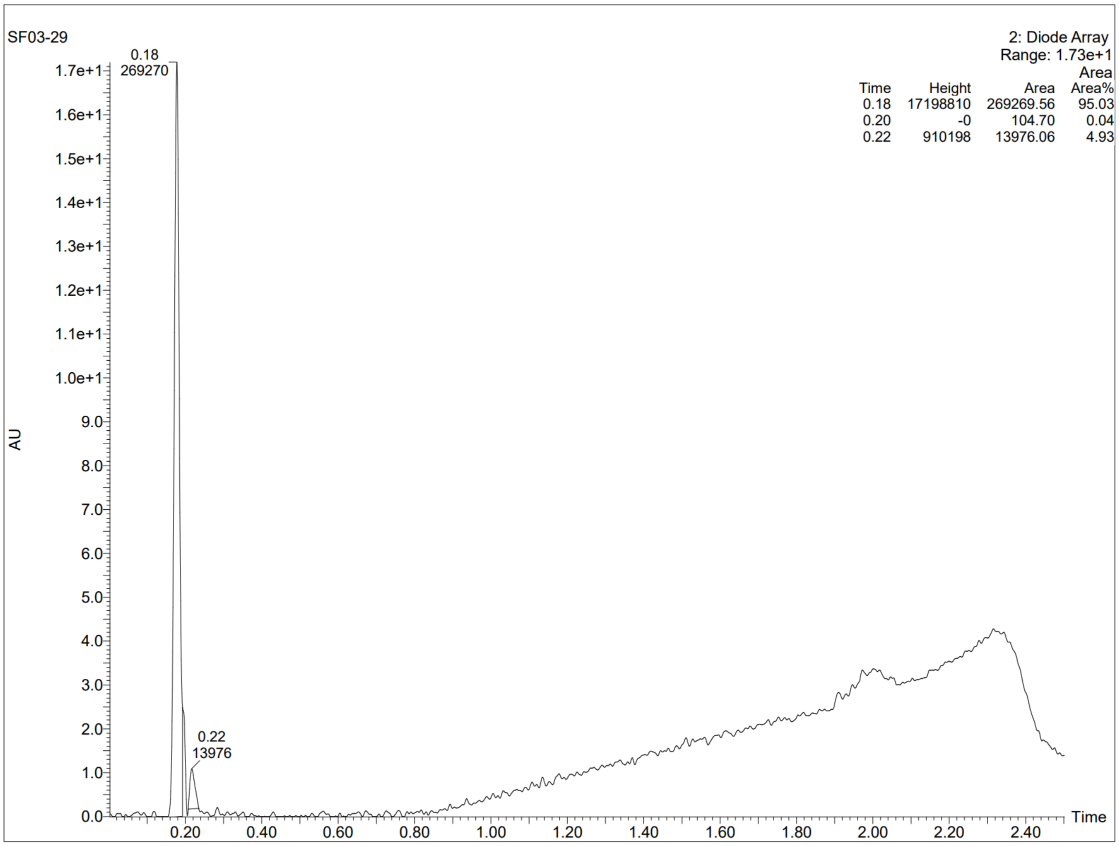

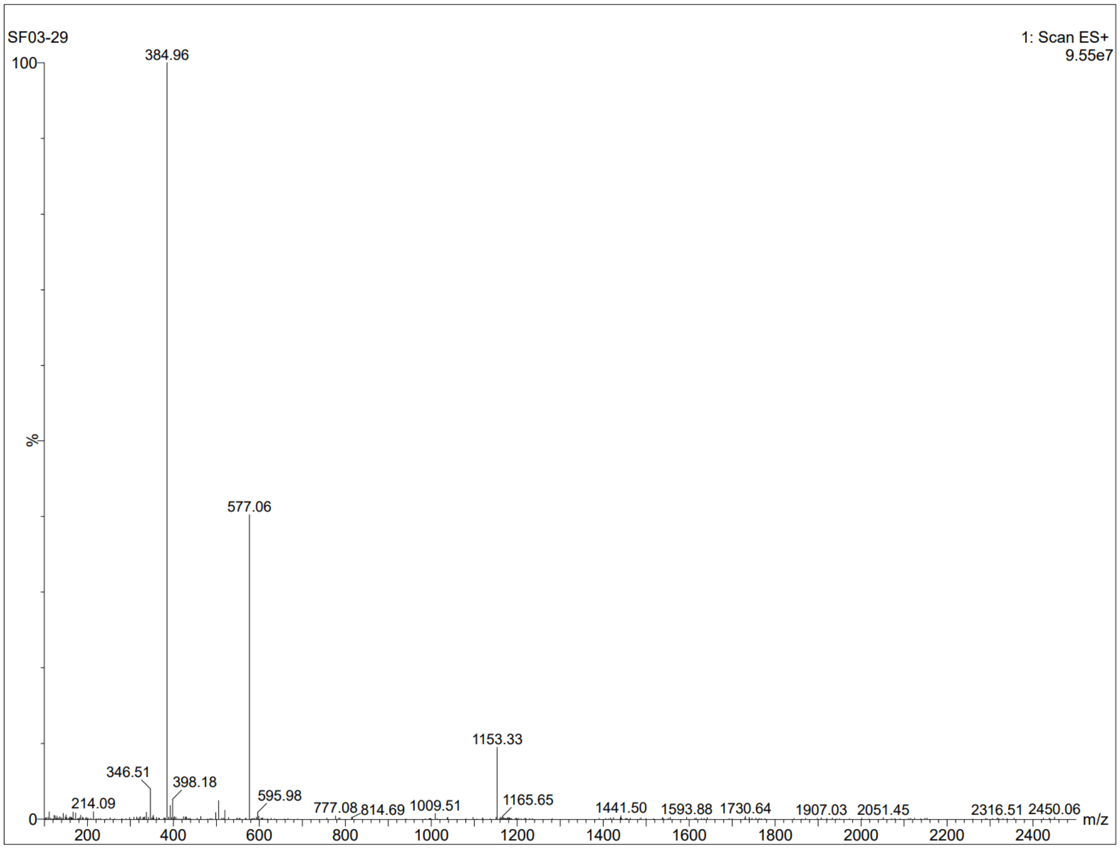

SF03-16: UPLC-MS

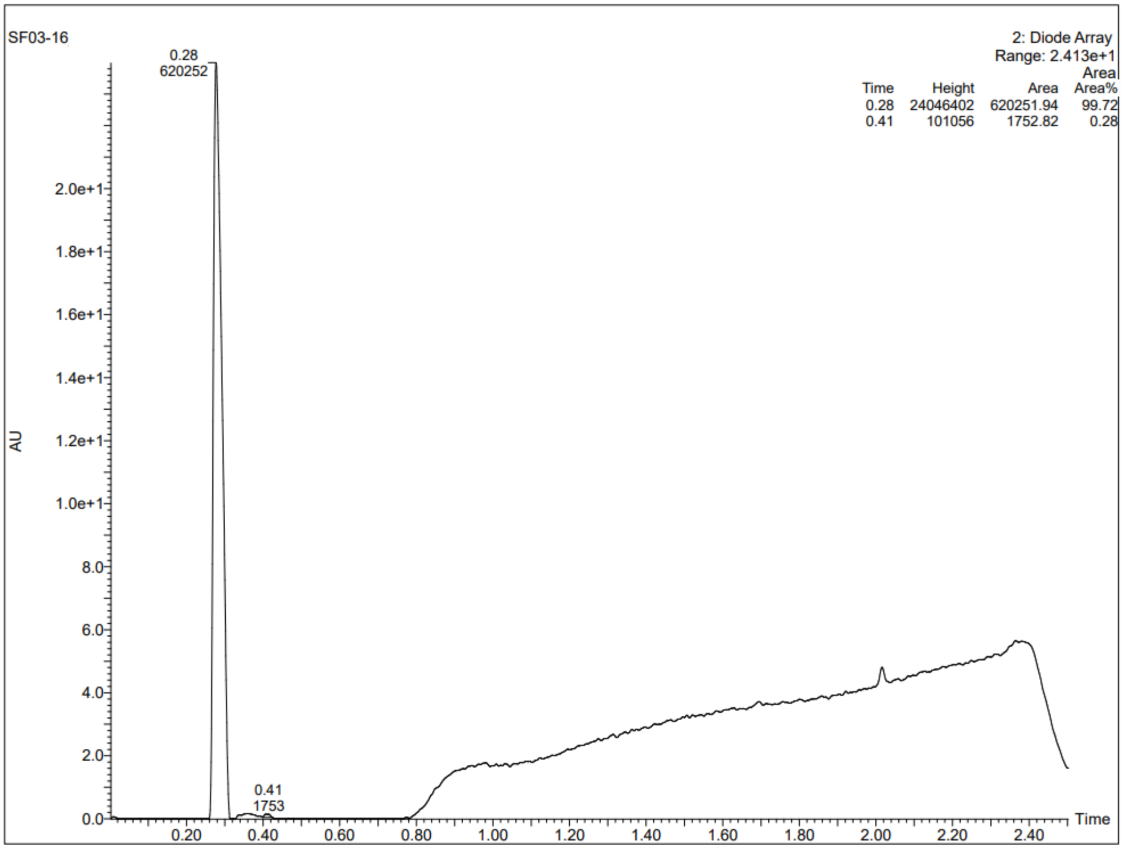

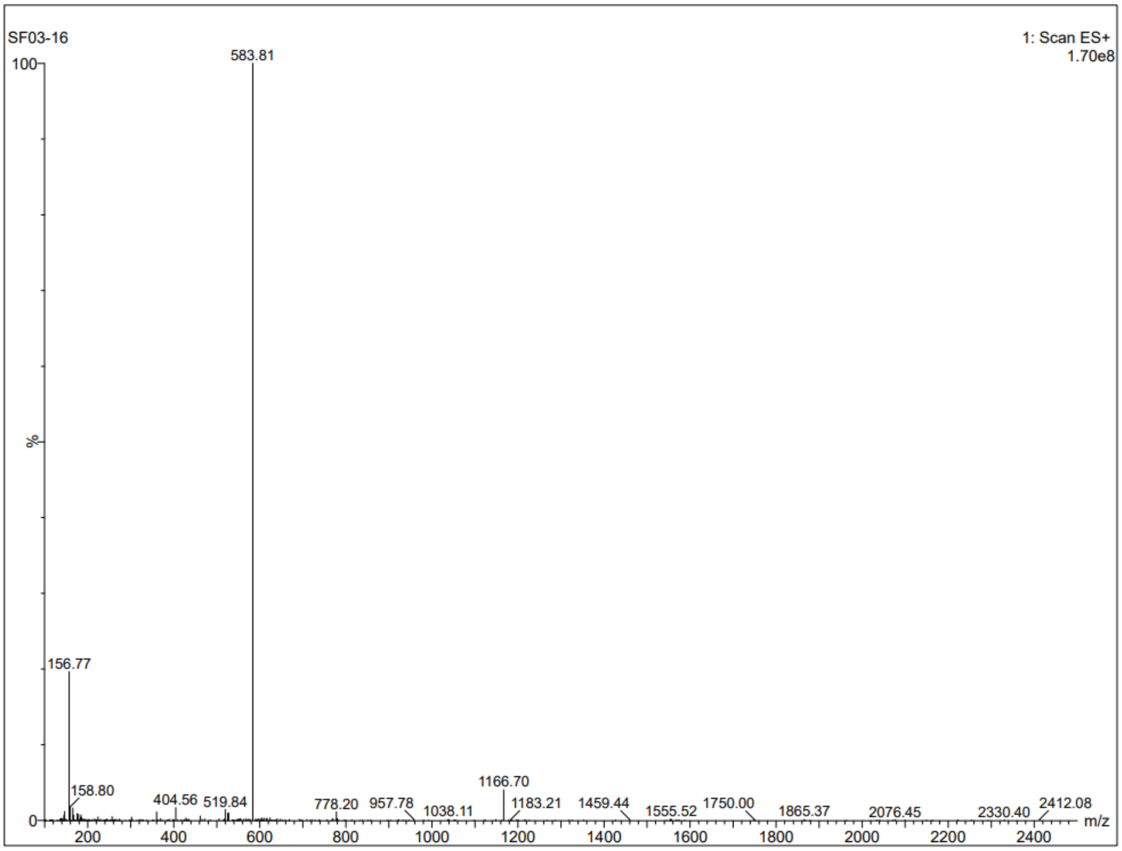

SF03-06_Product 1: UPLC-MS

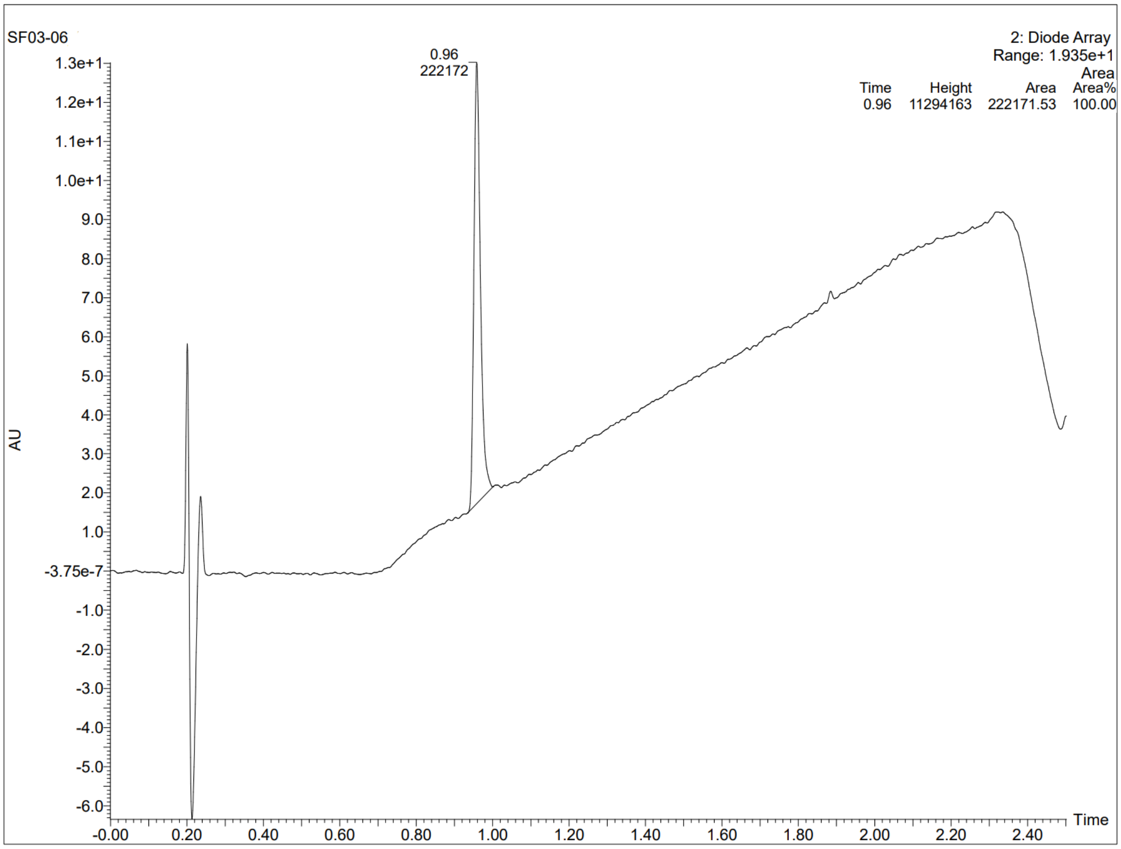

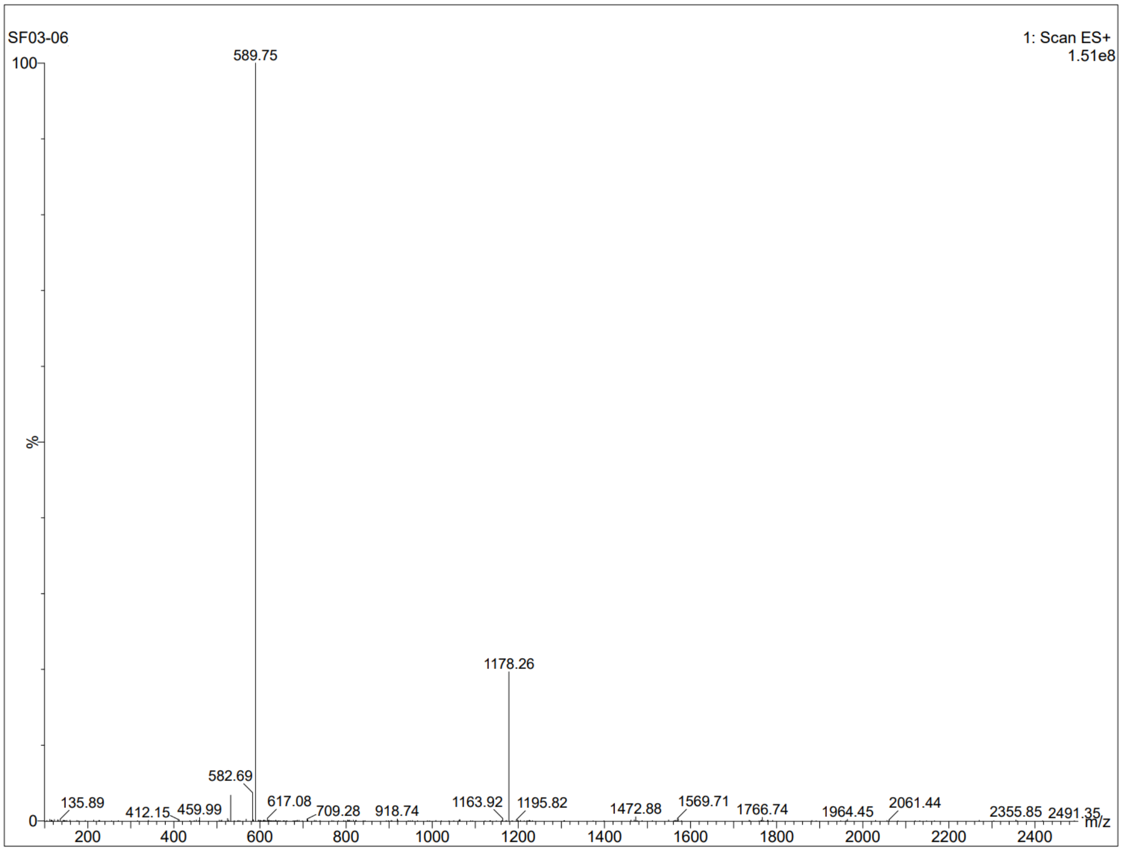

SF03-06_Product 2: UPLC-MS

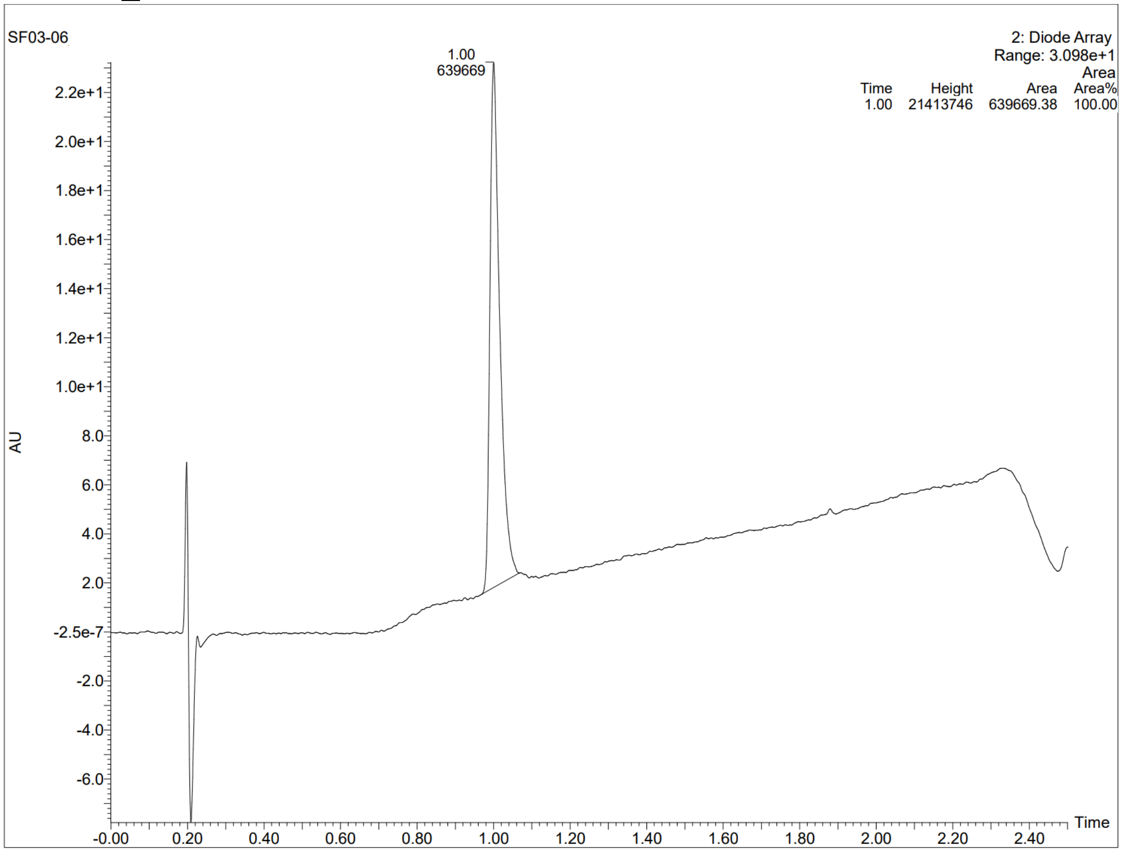

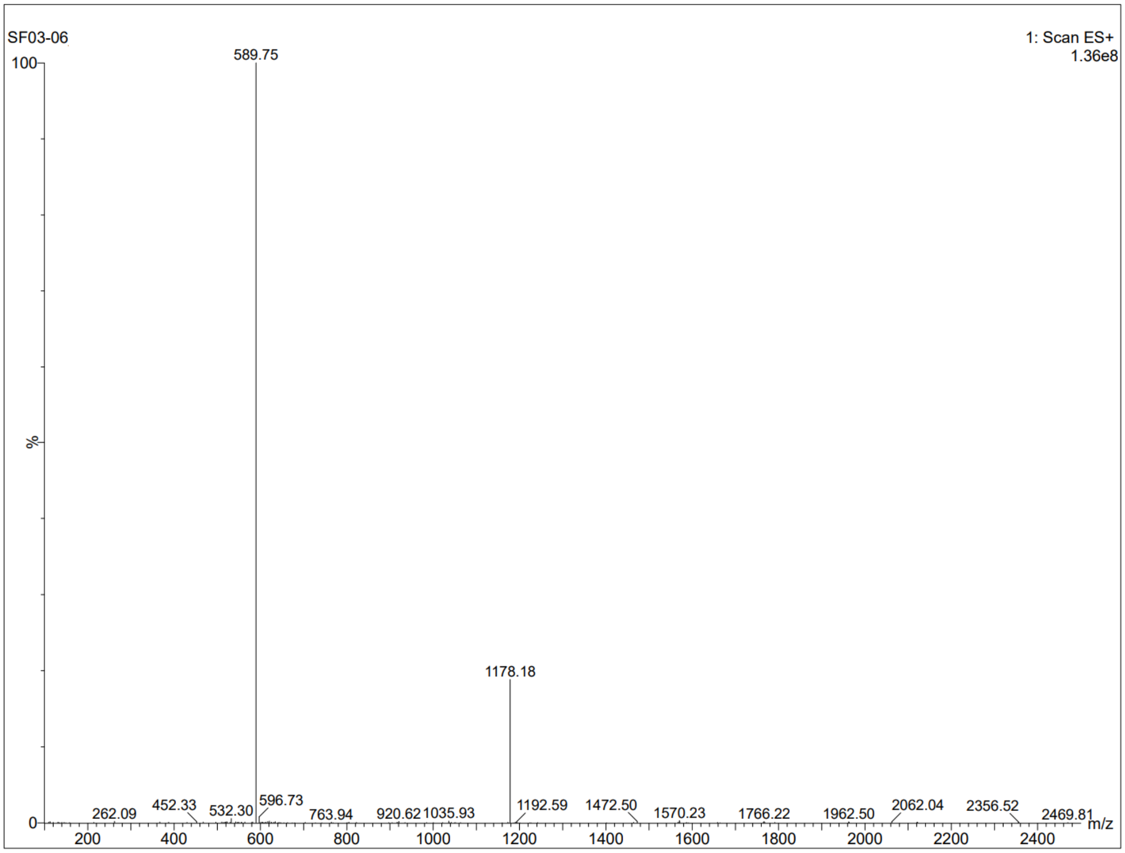

SF03-19: UPLC-MS

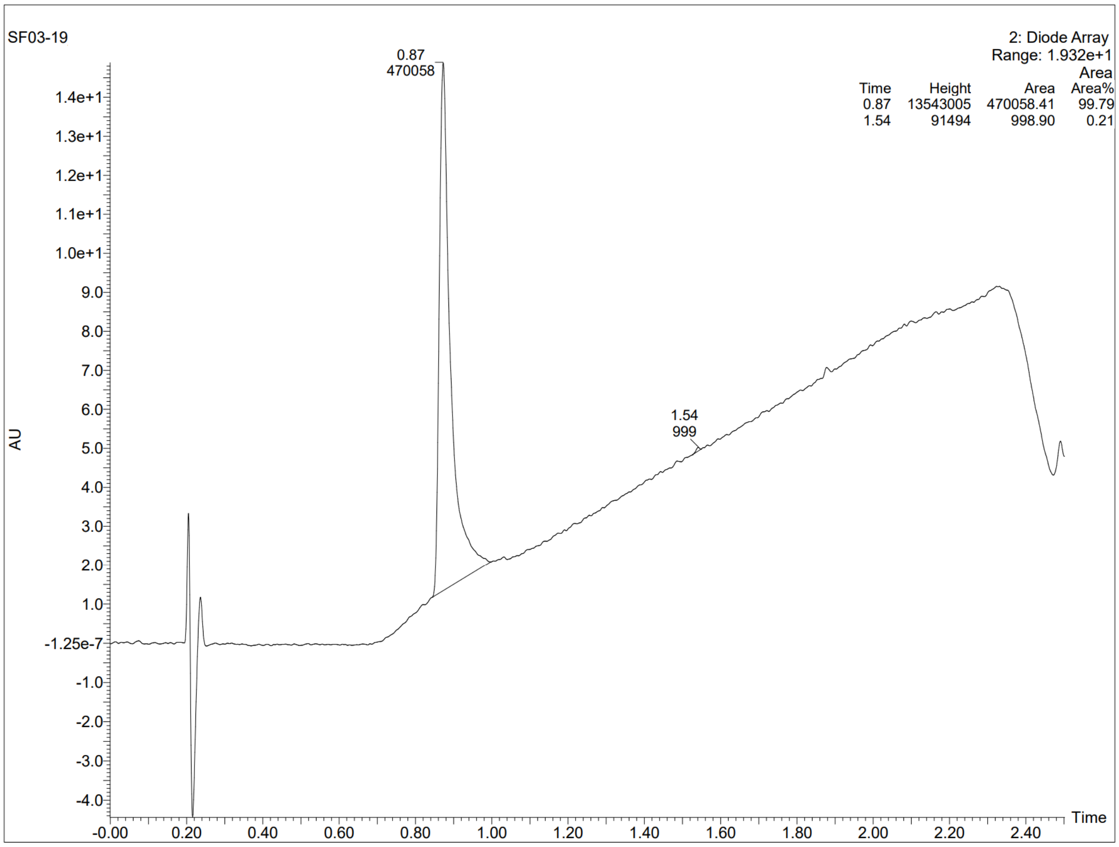

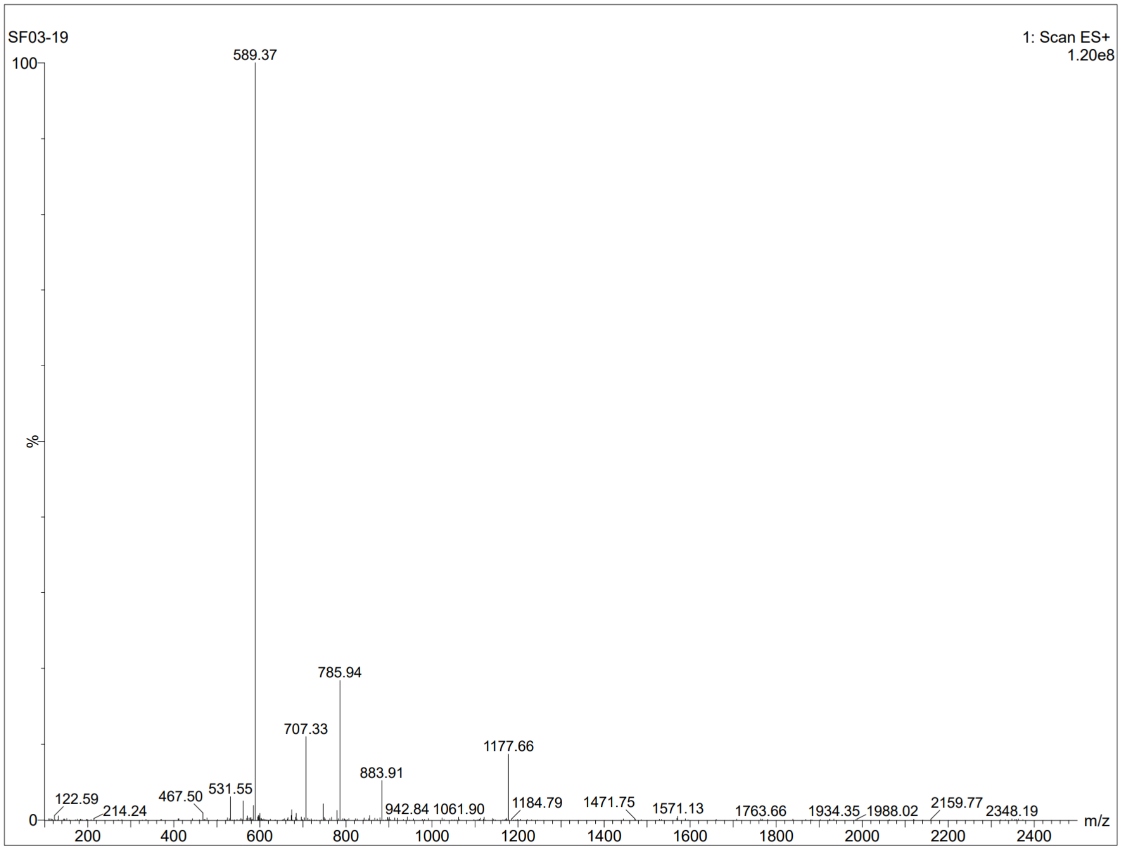

SF03-20: UPLC-MS

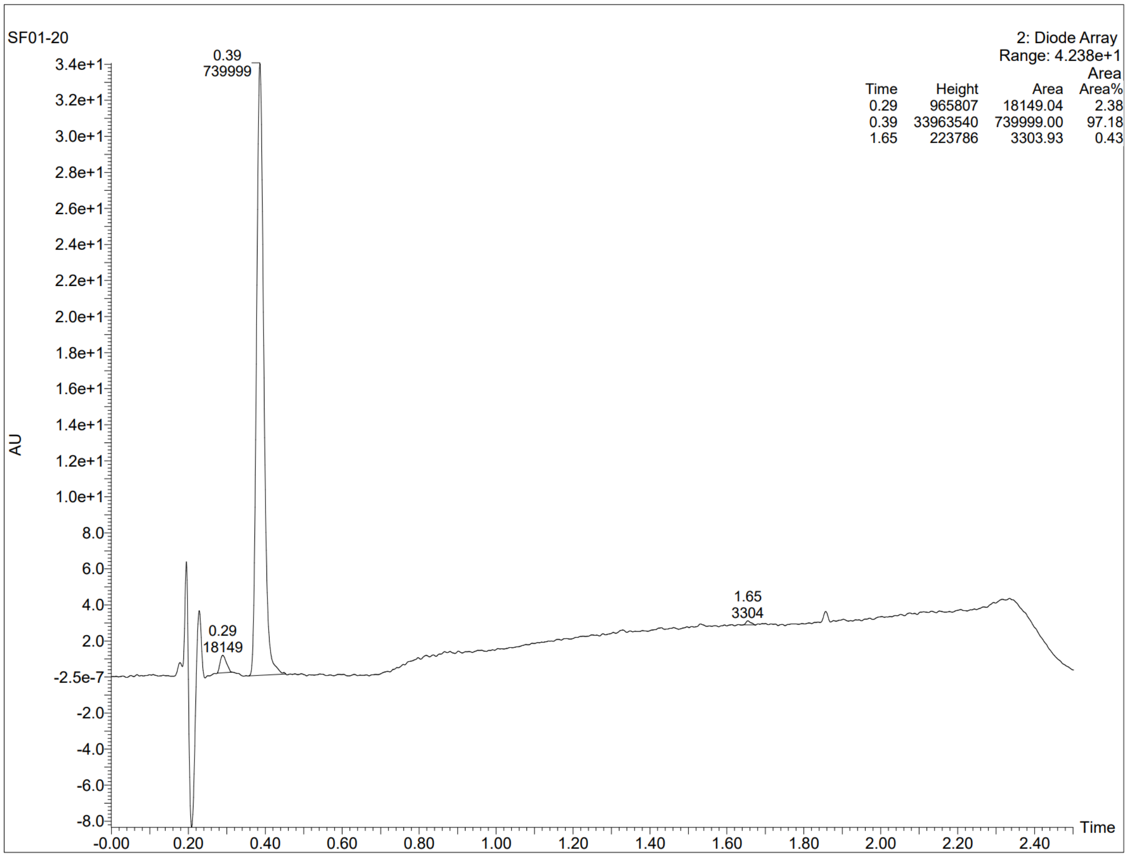

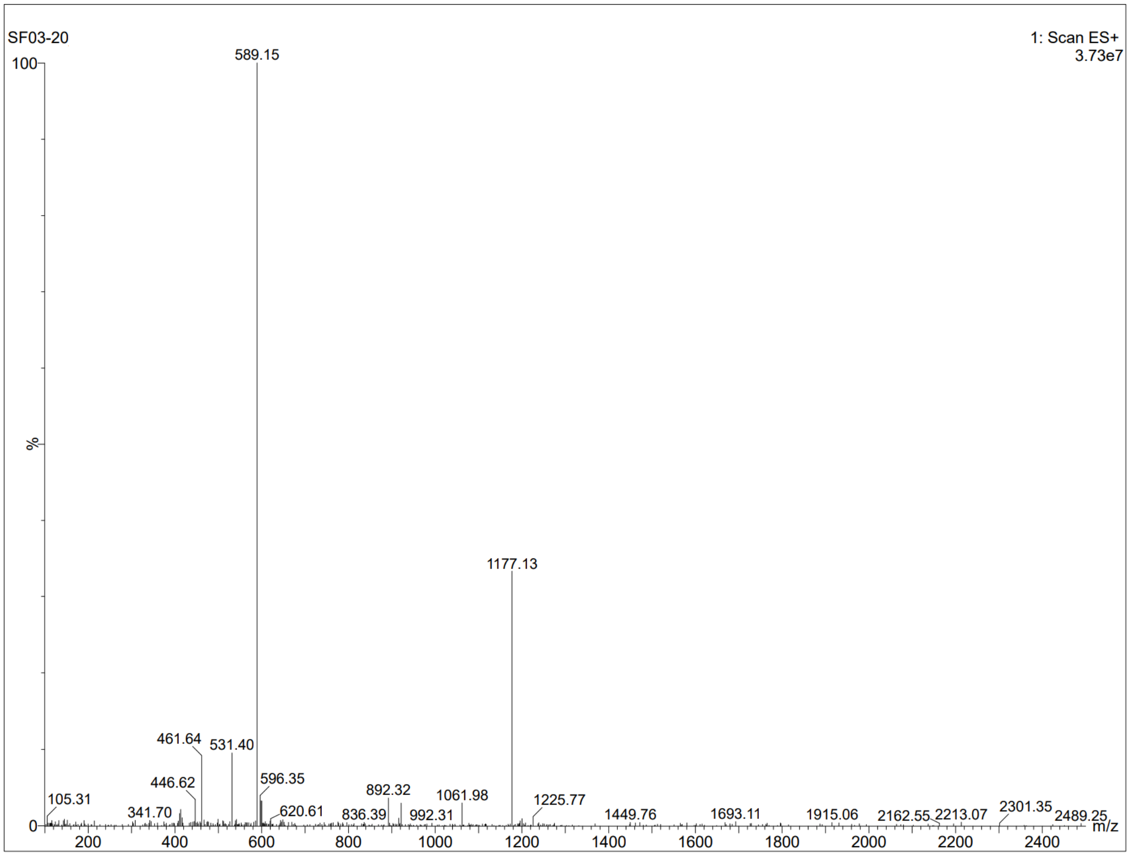

SF03-23: UPLC-MS

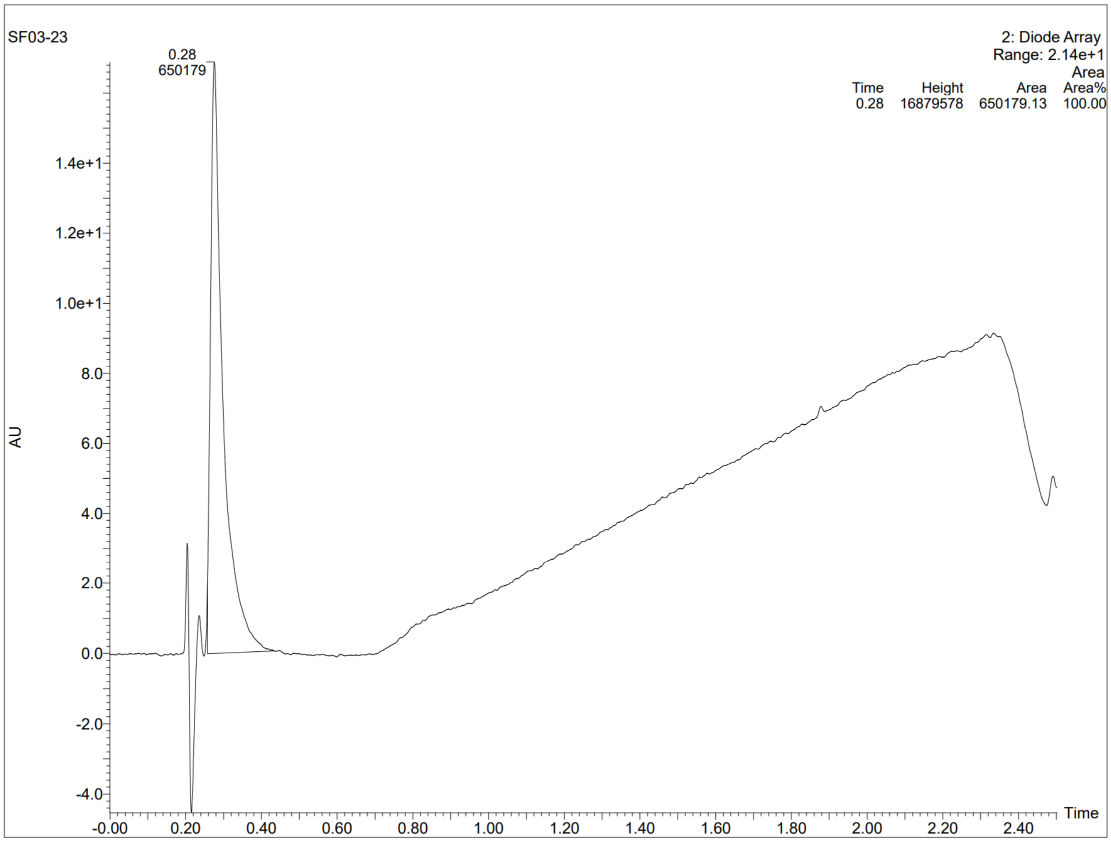

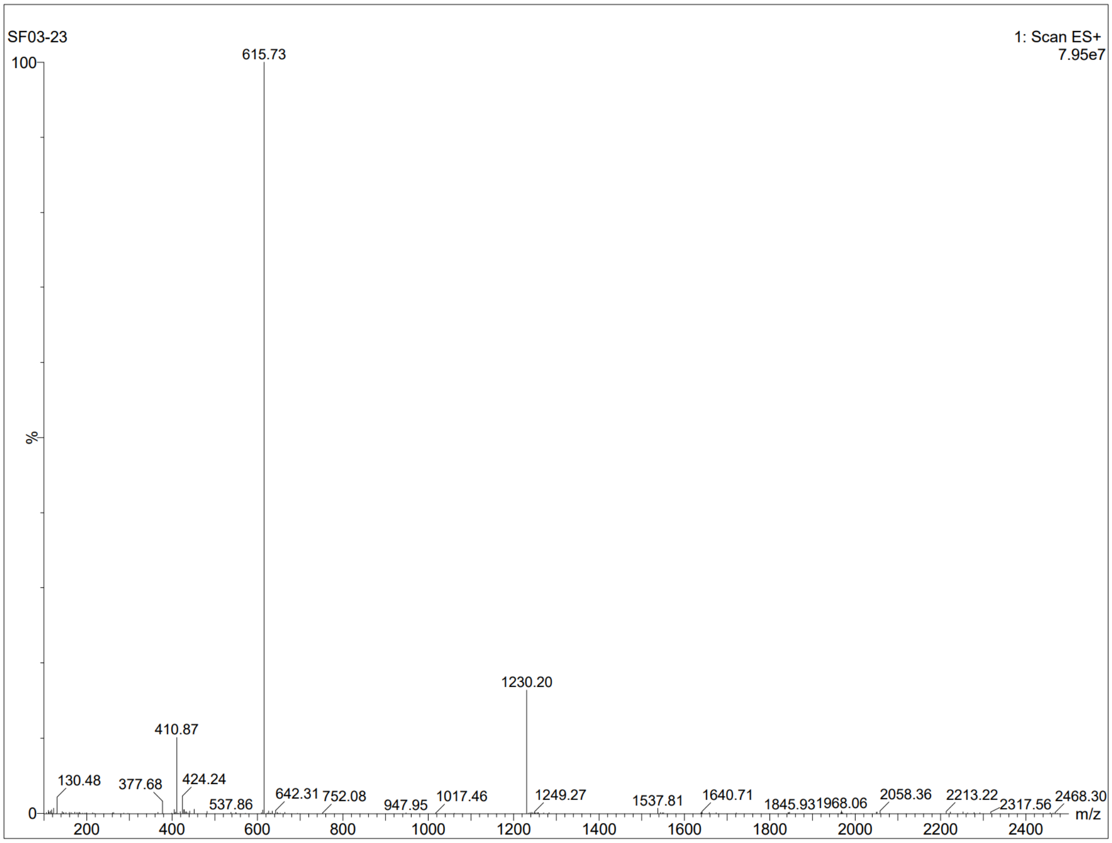

SF03-60: UPLC-MS

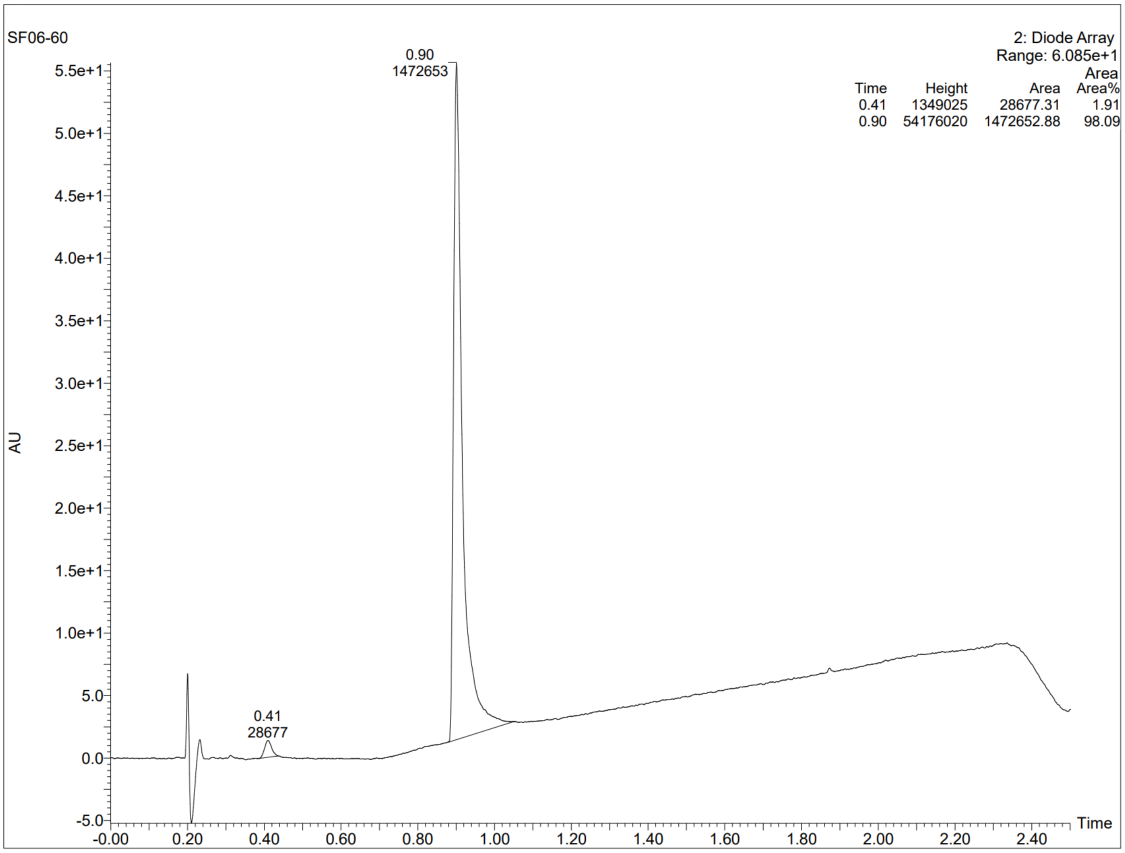

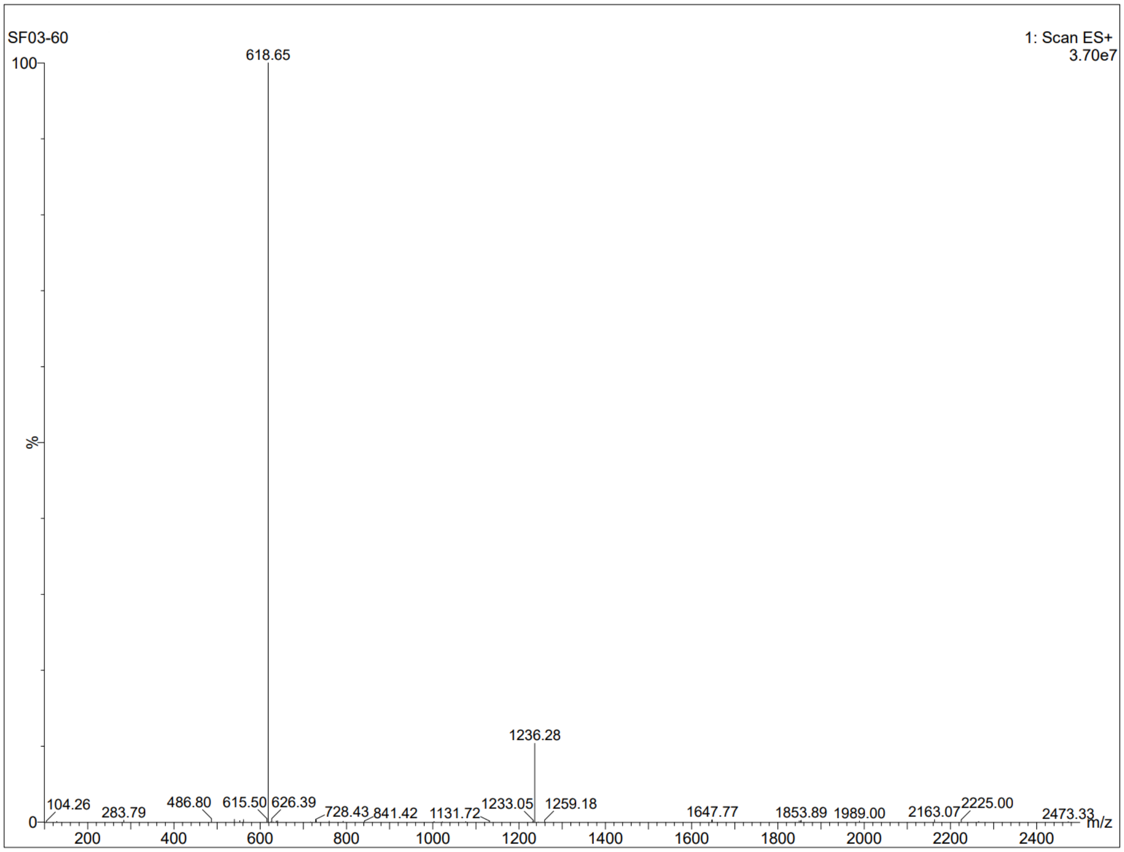

SF03-62: UPLC-MS

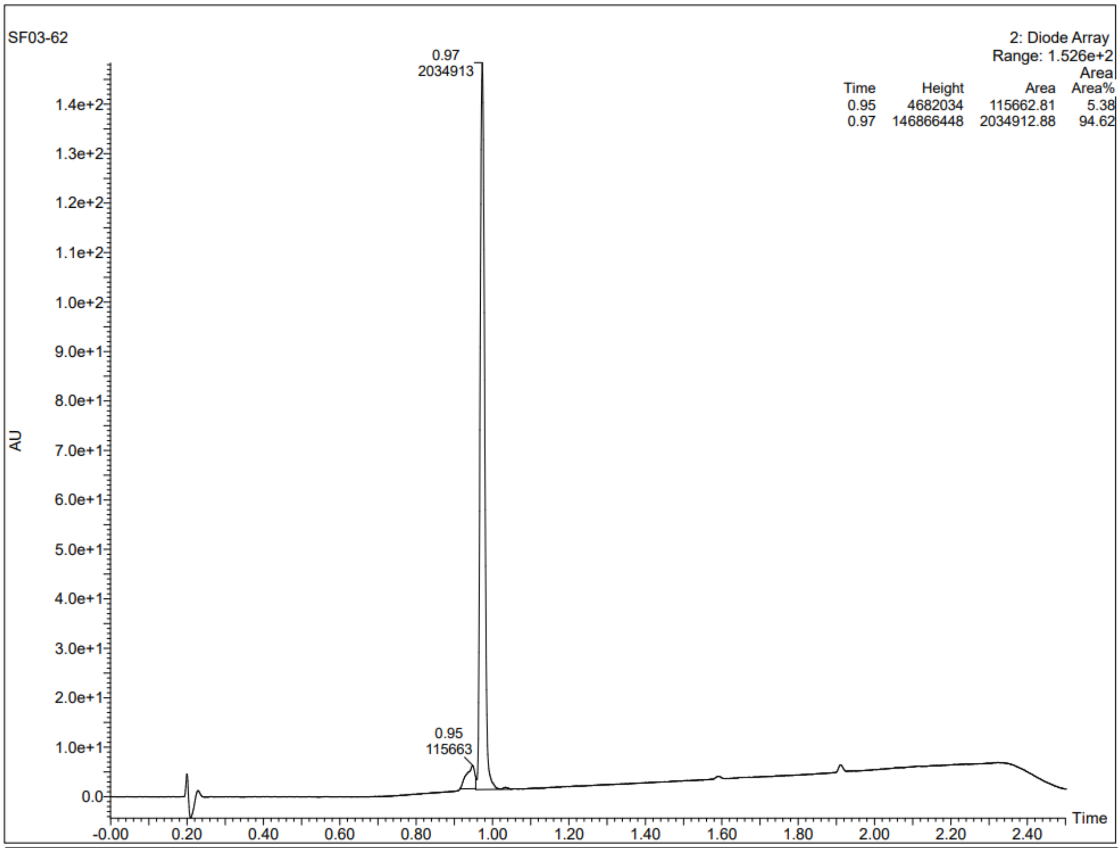

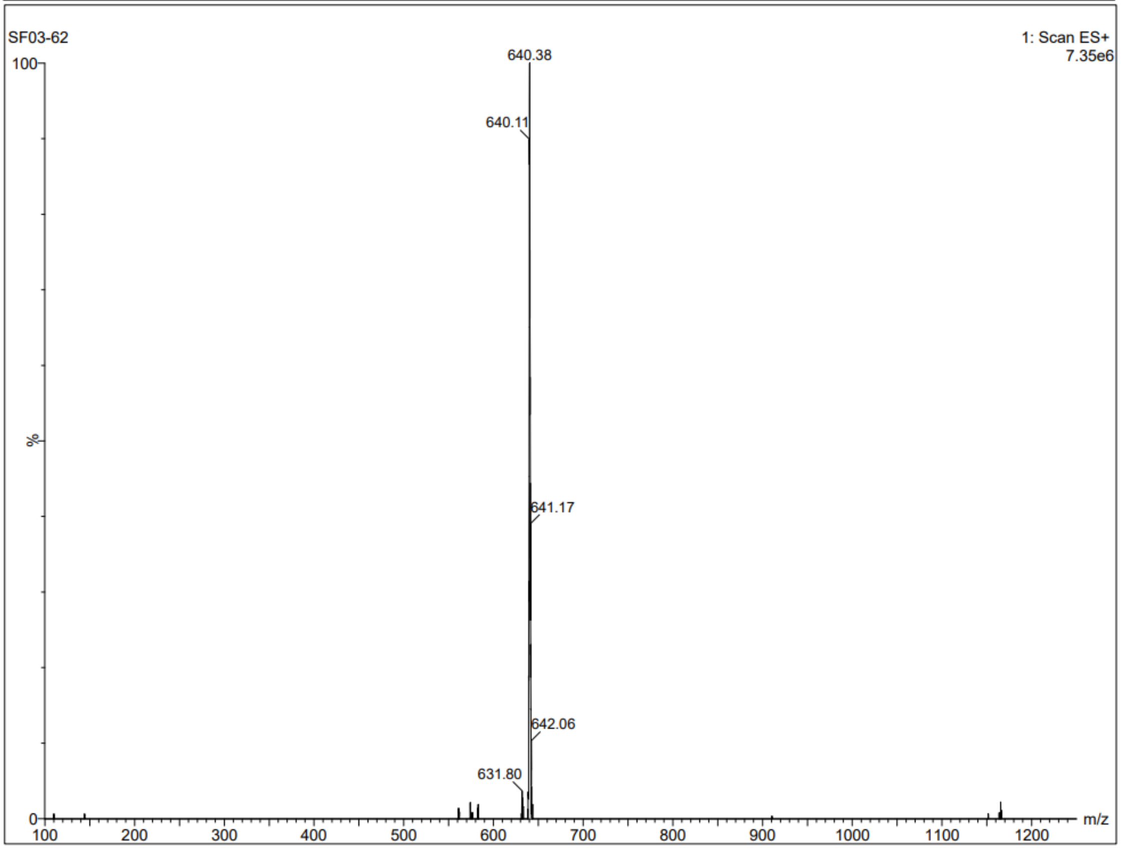

SF03-41: UPLC-MS

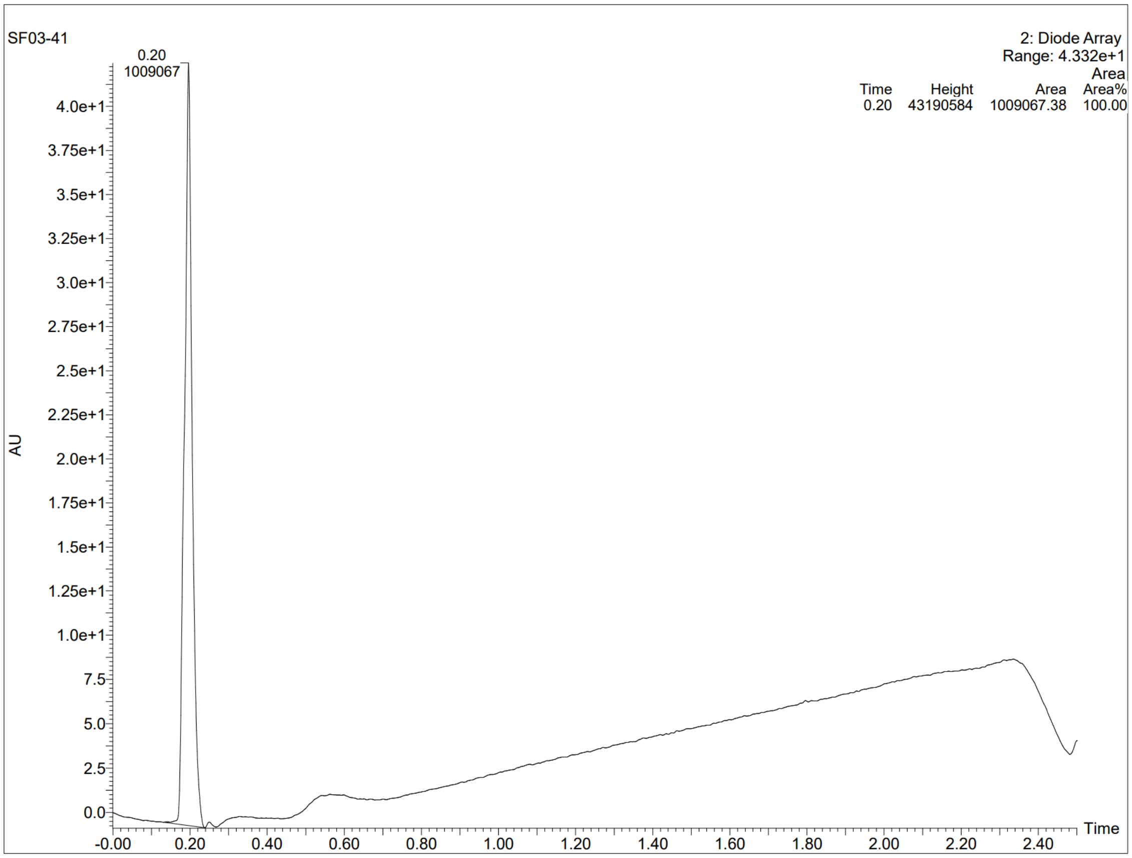

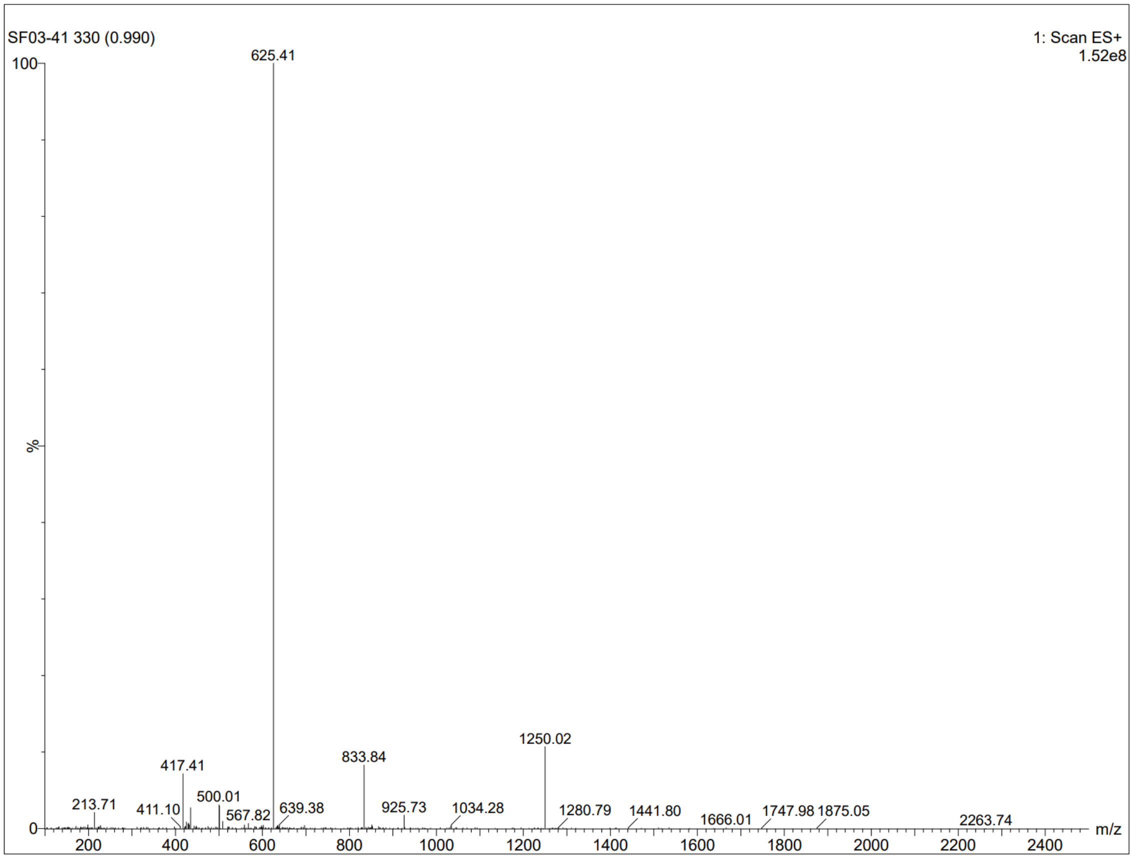

SF03-14: UPLC-MS

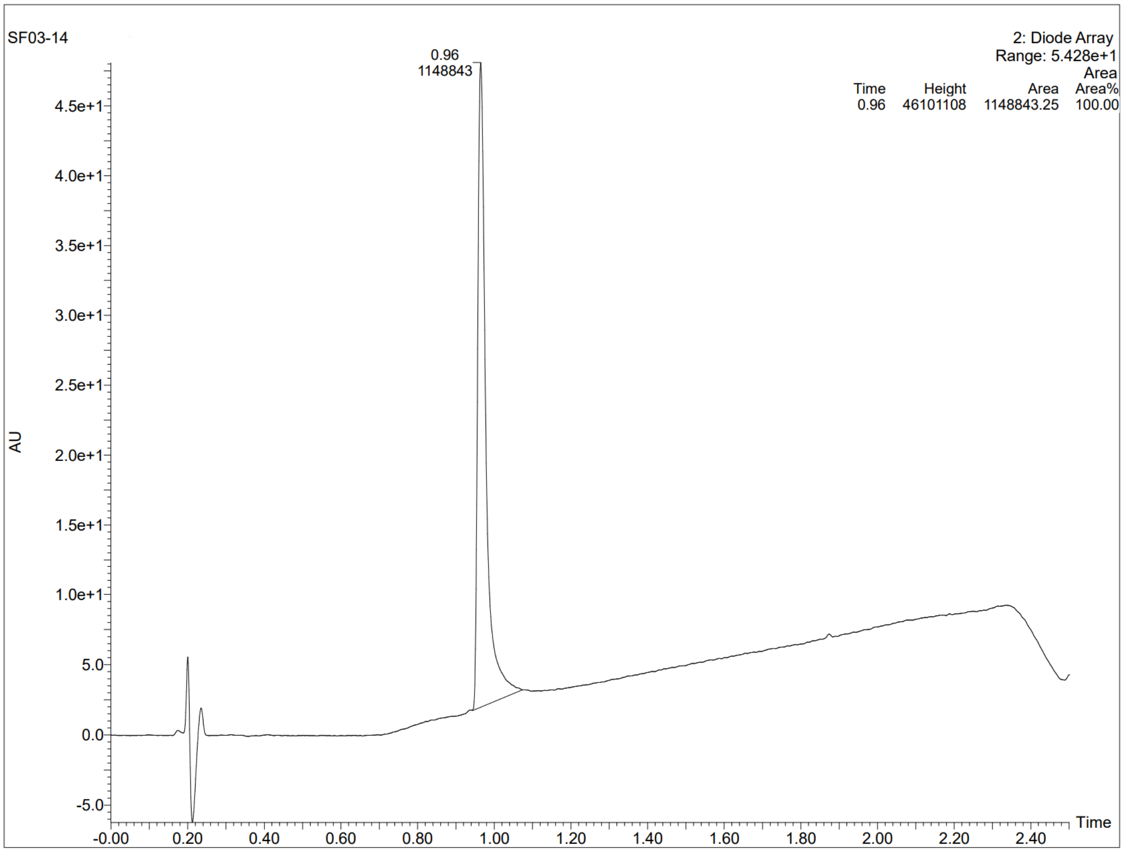

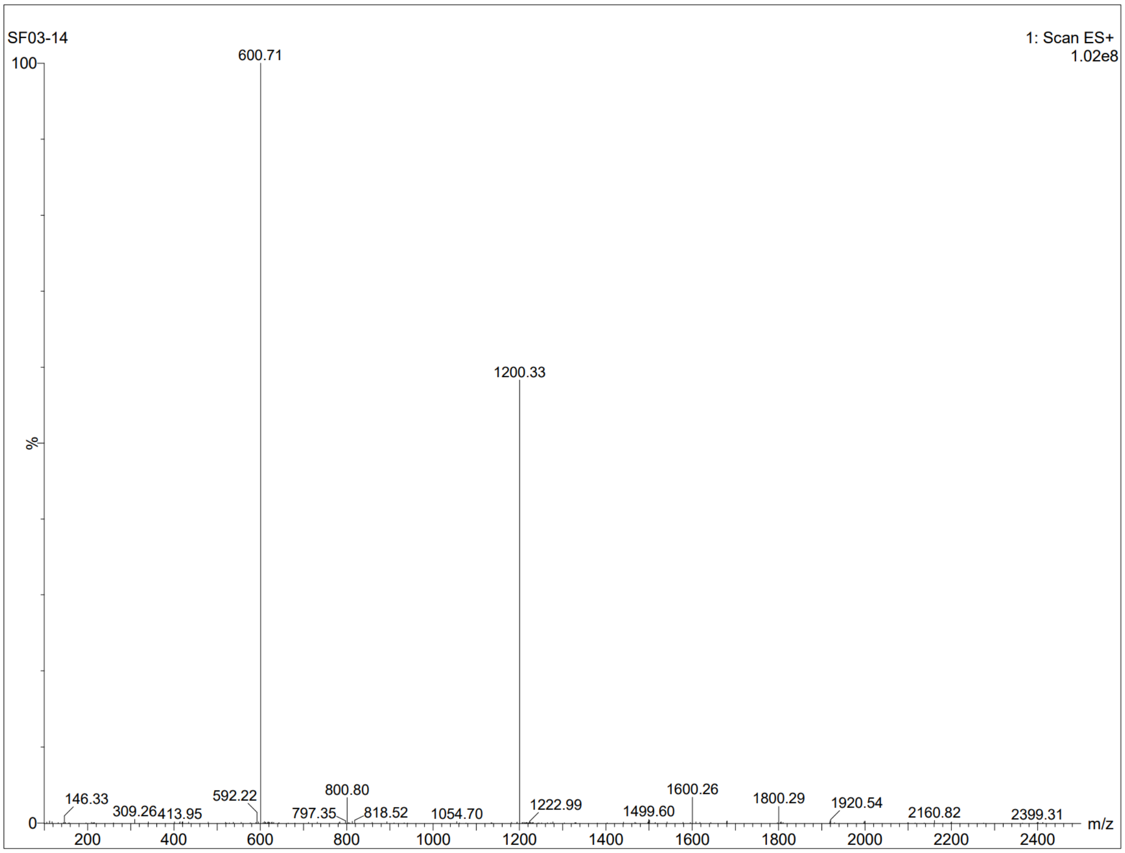

SF03-30: UPLC-MS

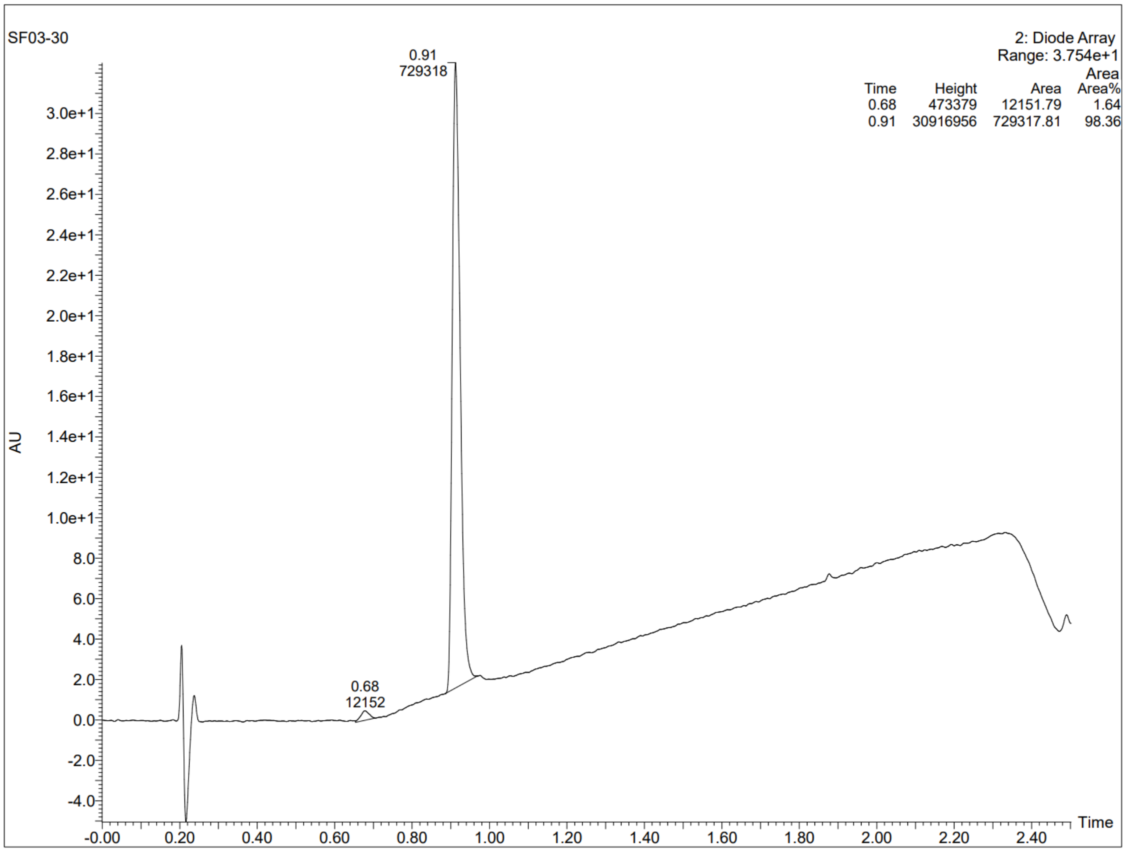

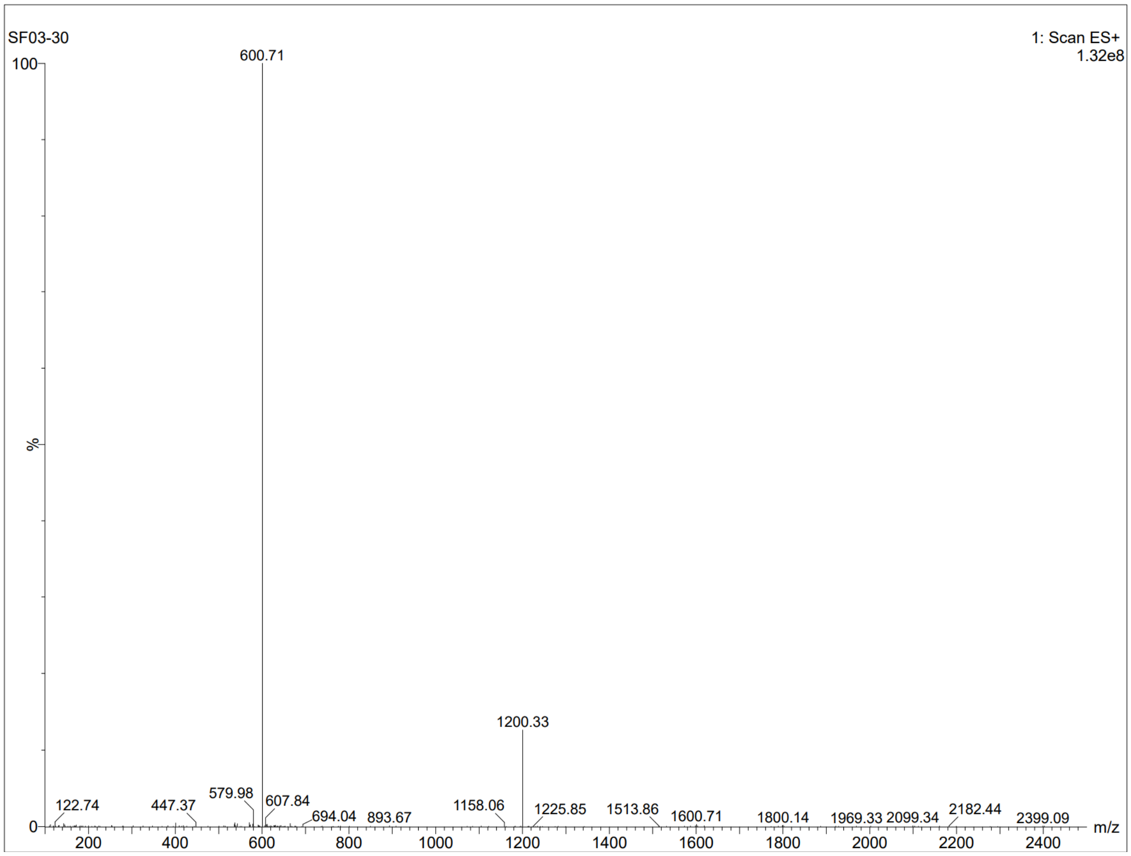

SF03-17: UPLC-MS

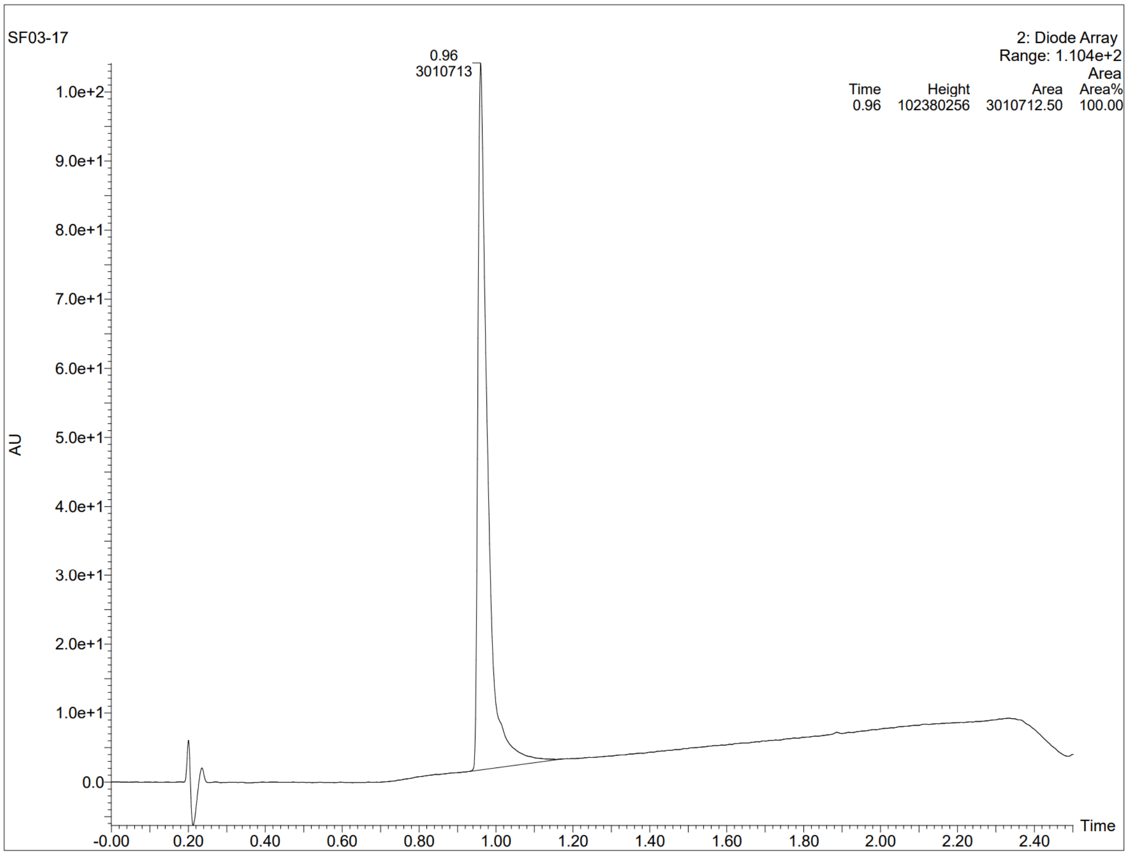

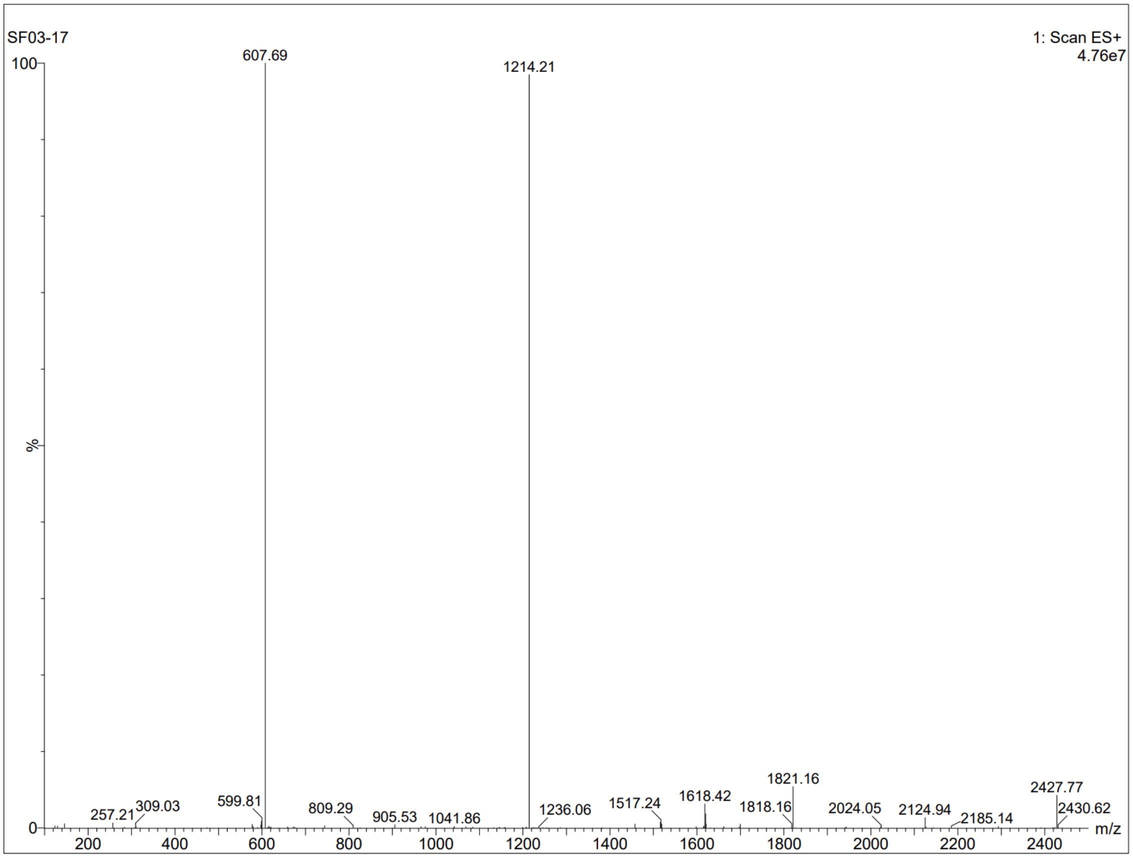

SF03-08_Product 2: UPLC-MS

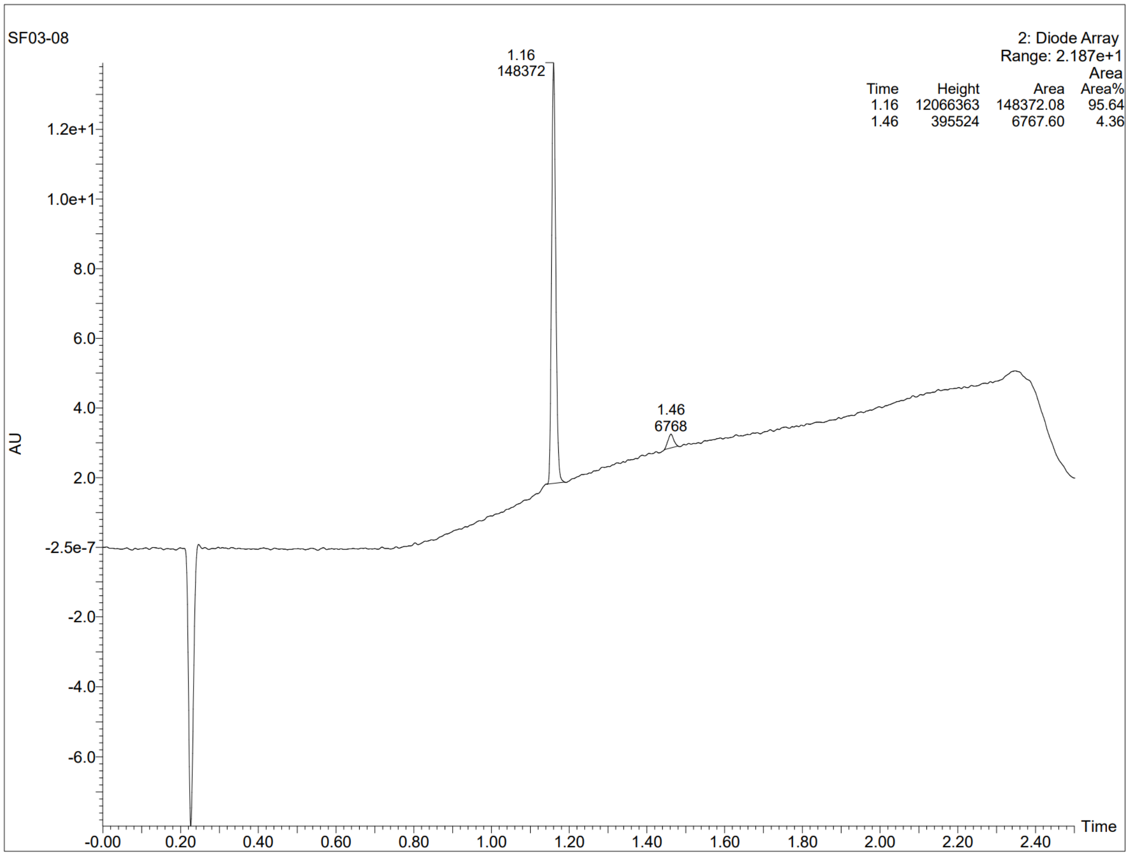

Time	Height	Area	Area%
1.16	12066363	148372.08	95.64
1.46	395524	6767.60	4.36

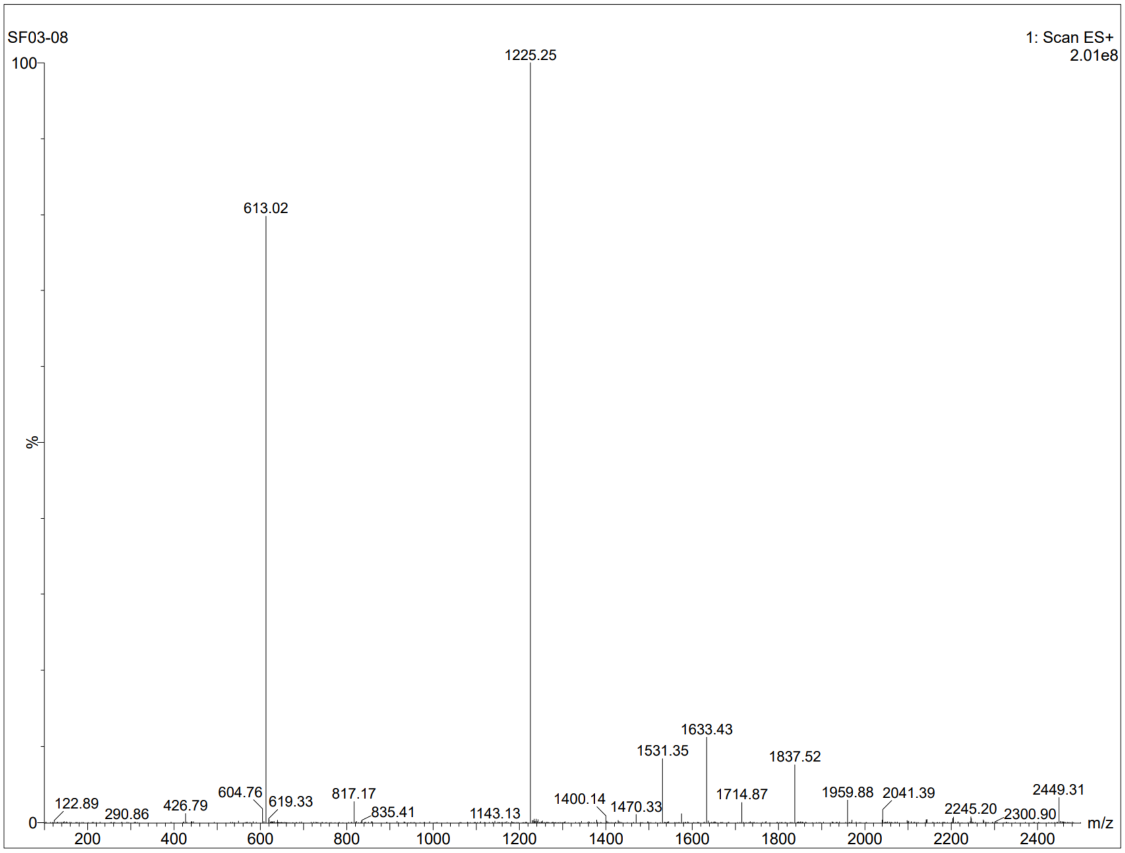

SF03-21: UPLC-MS

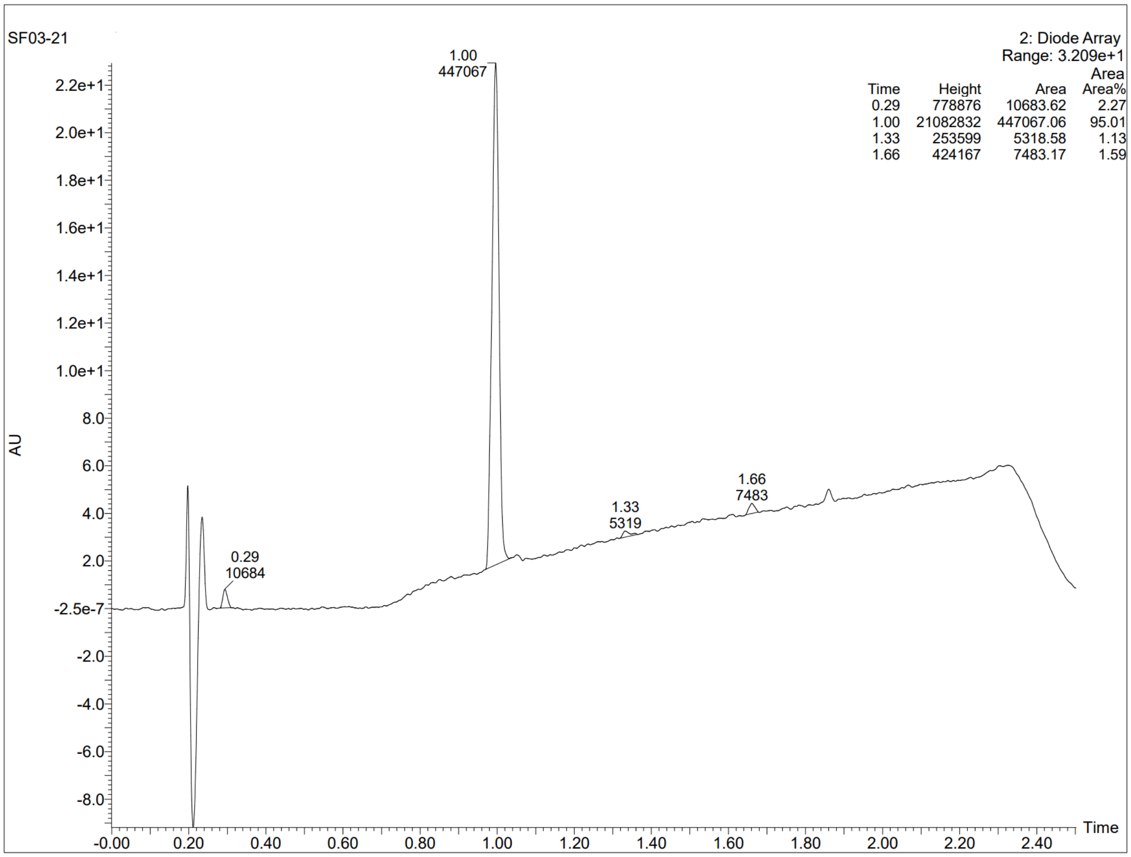

Time	Height	Area	Area%
0.29	778876	10683.62	2.27
1.00	21082832	447067.06	95.01
1.33	253599	5318.58	1.13
1.66	424167	7483.17	1.59

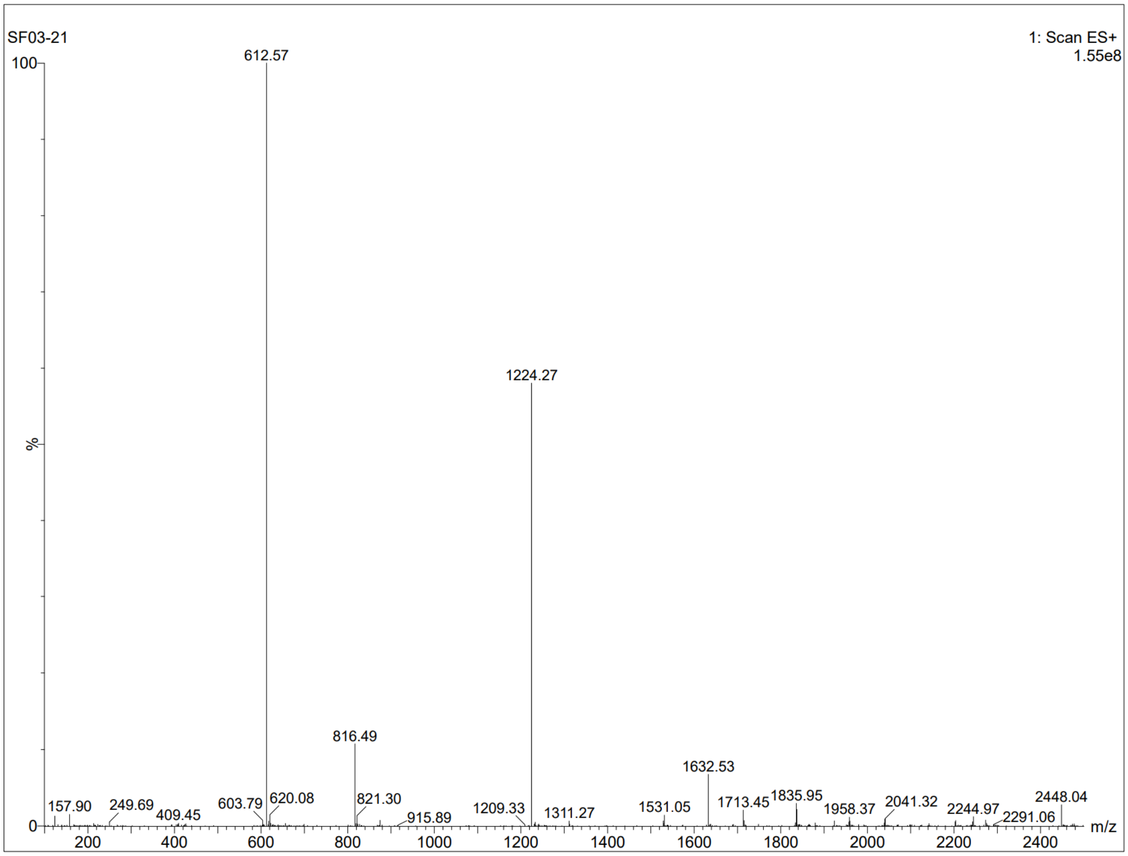

SF03-22: UPLC-MS

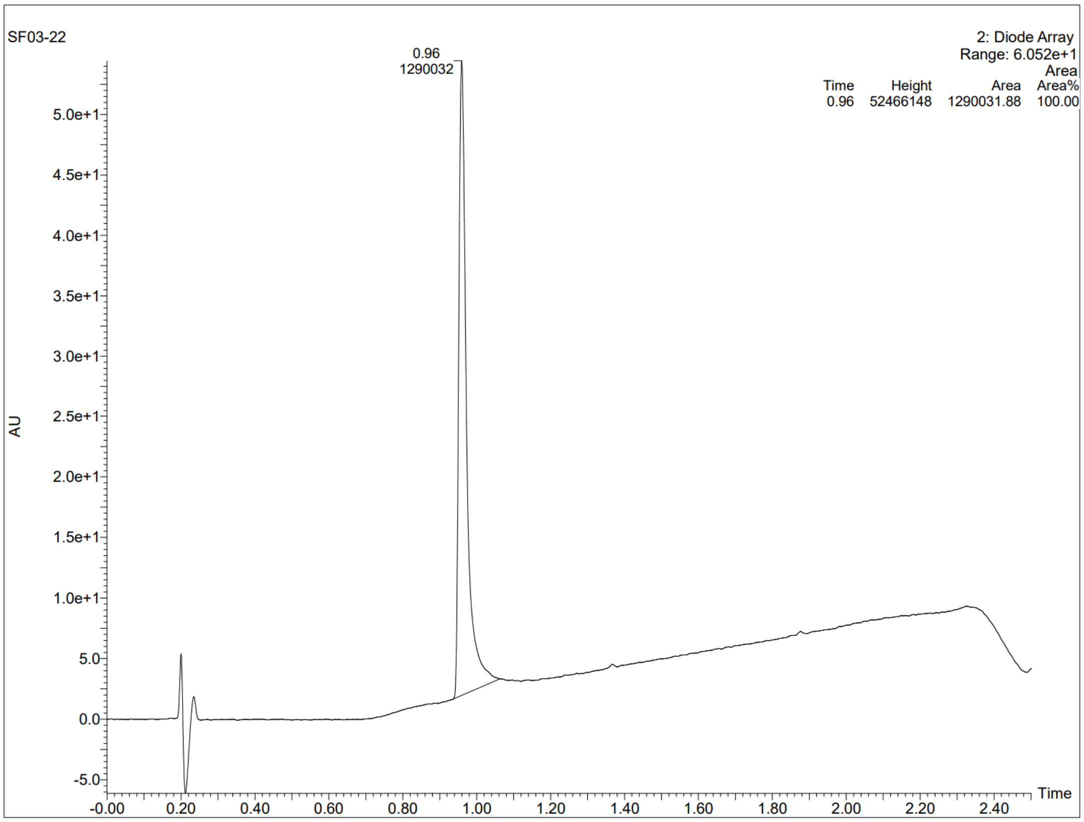

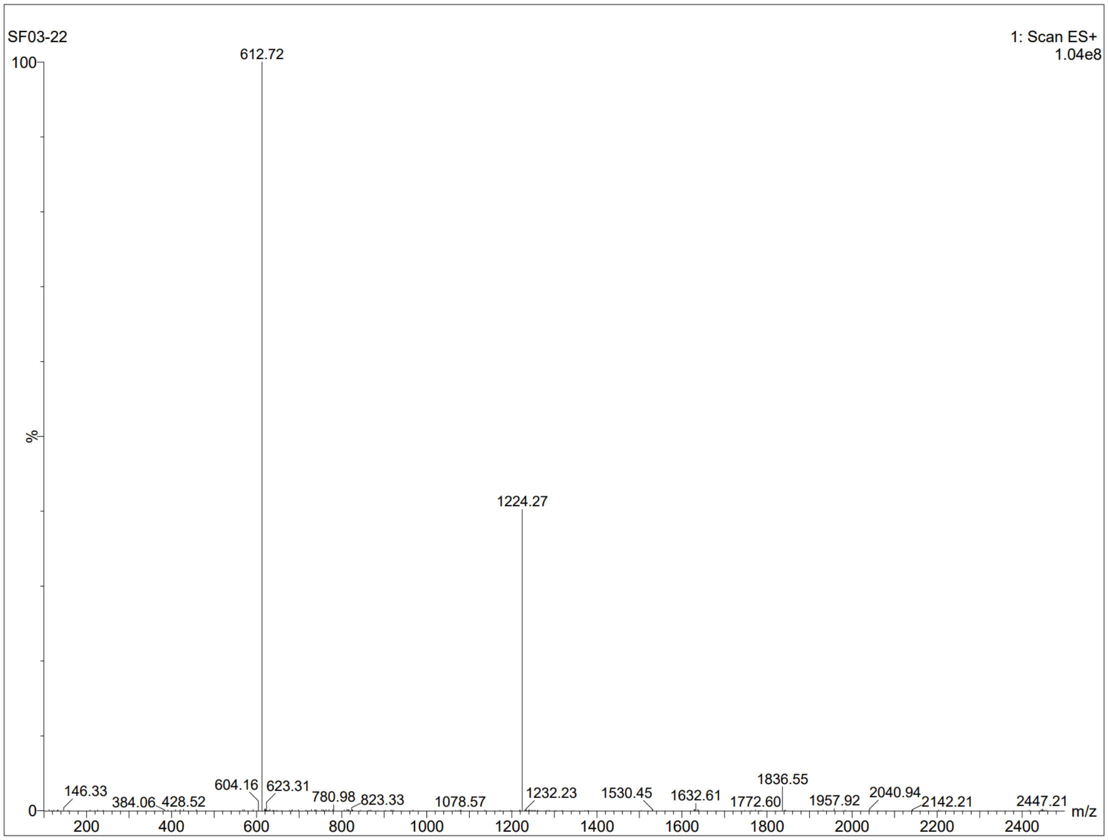

SF03-24: UPLC-MS

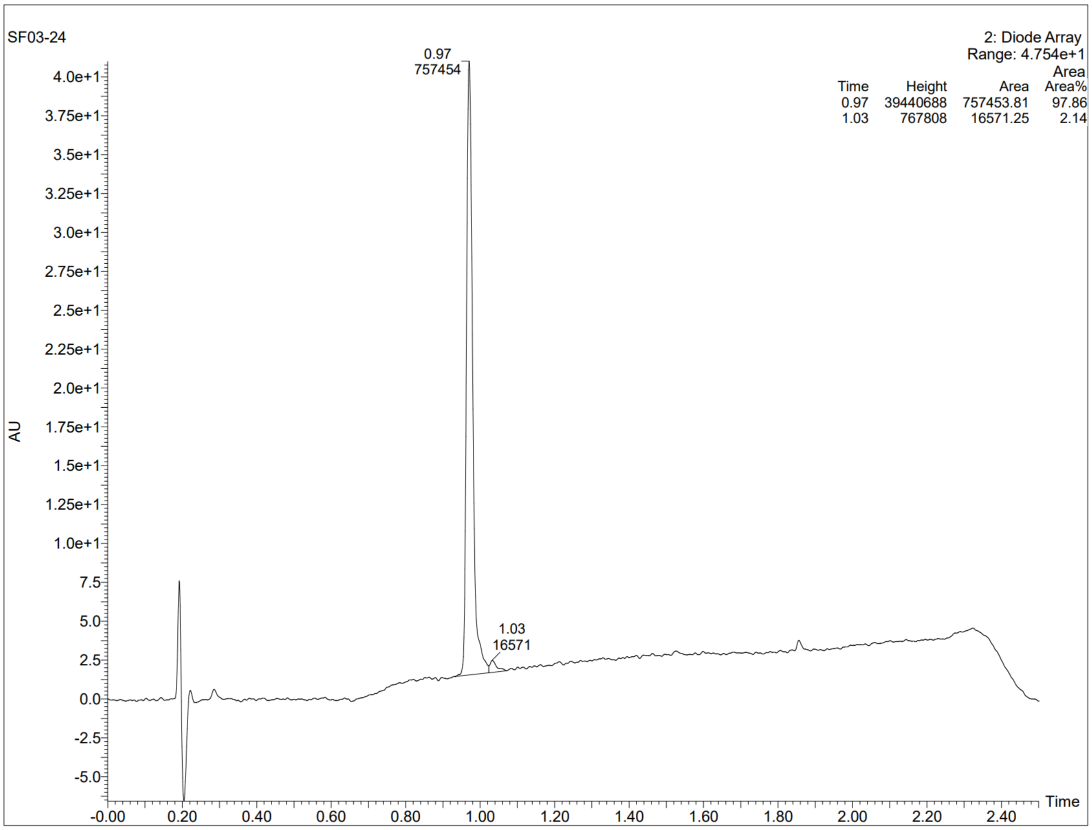

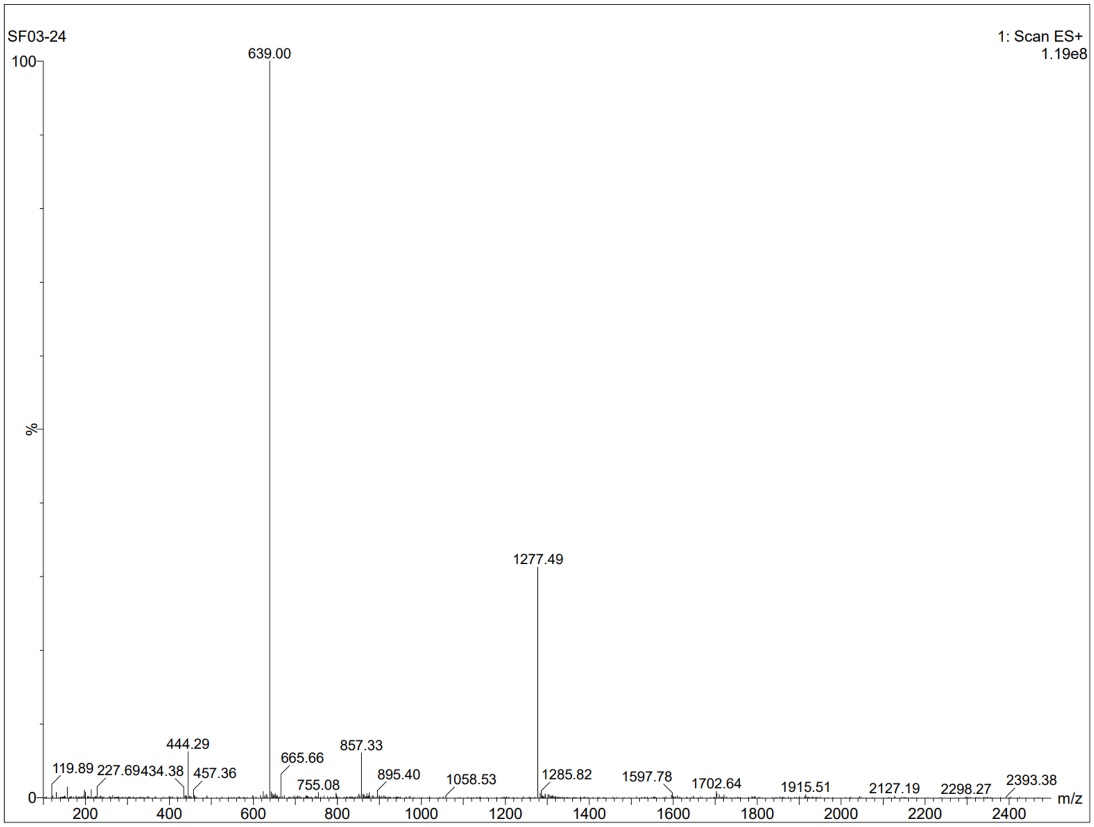

SF03-61: UPLC-MS

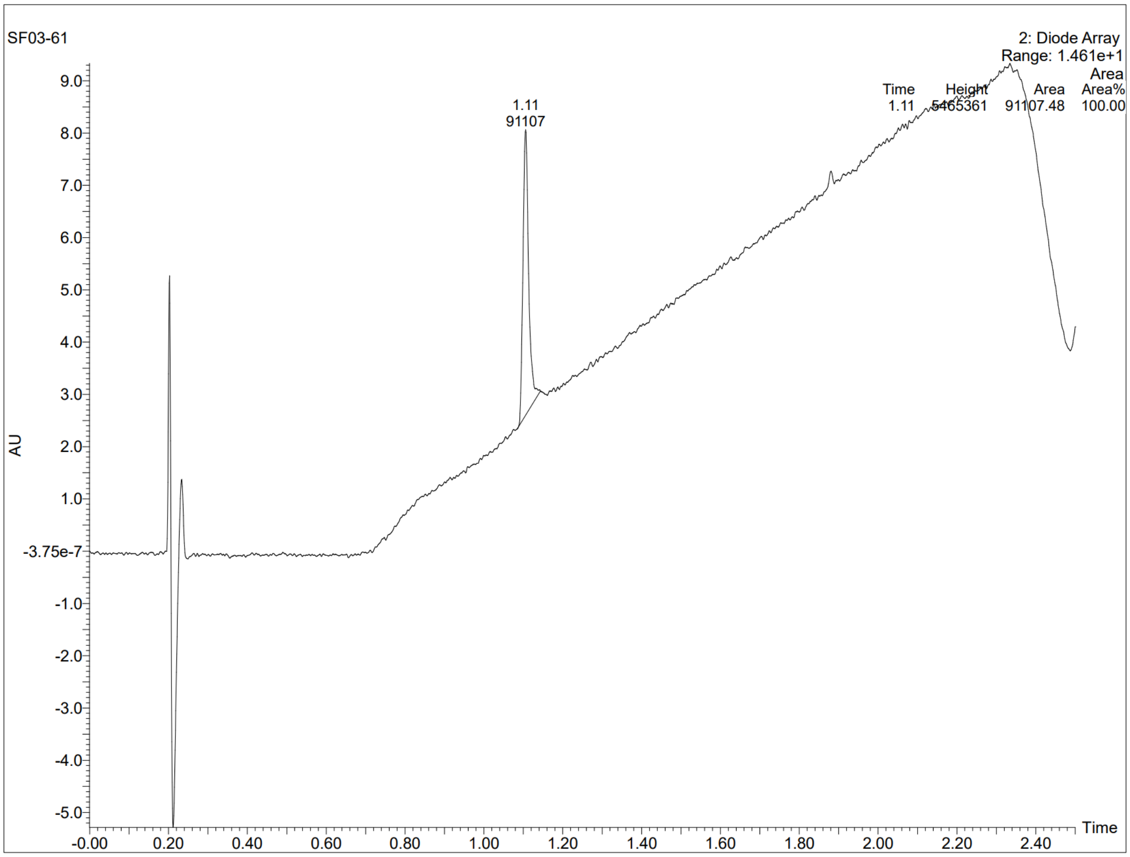

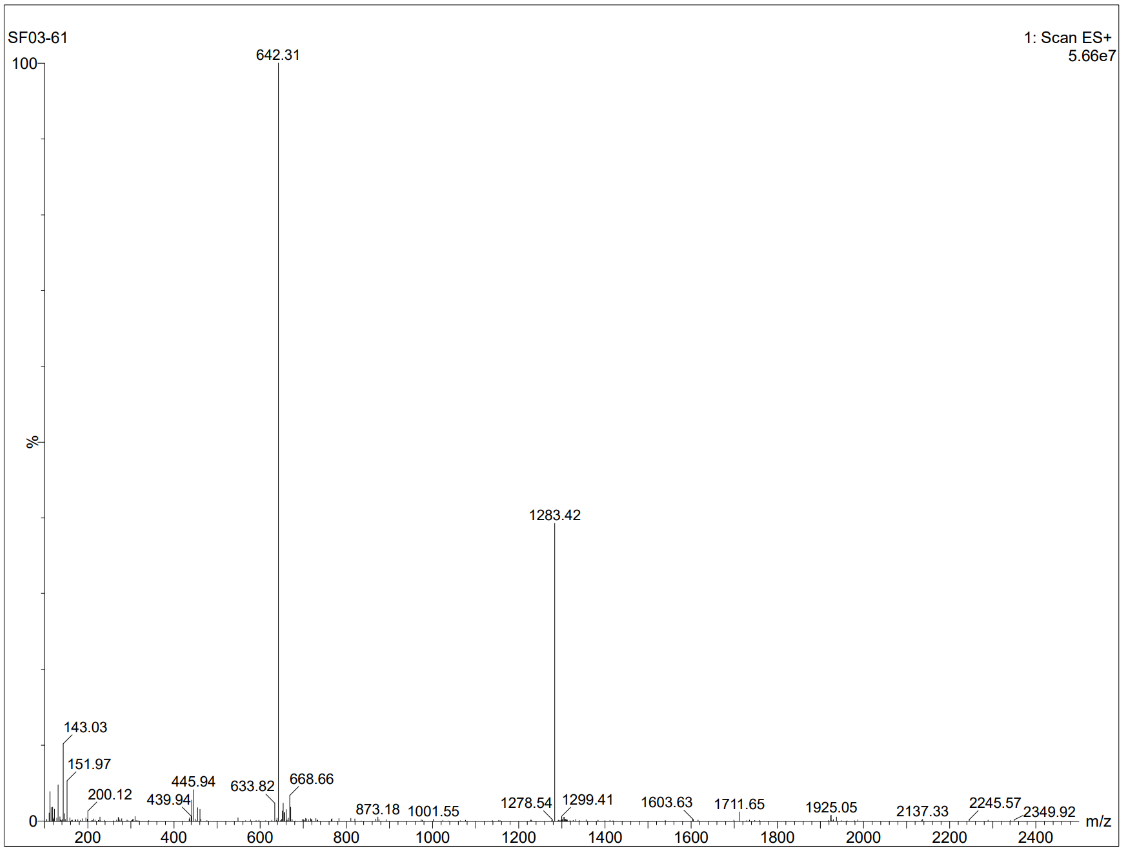

SF03-63: UPLC-MS

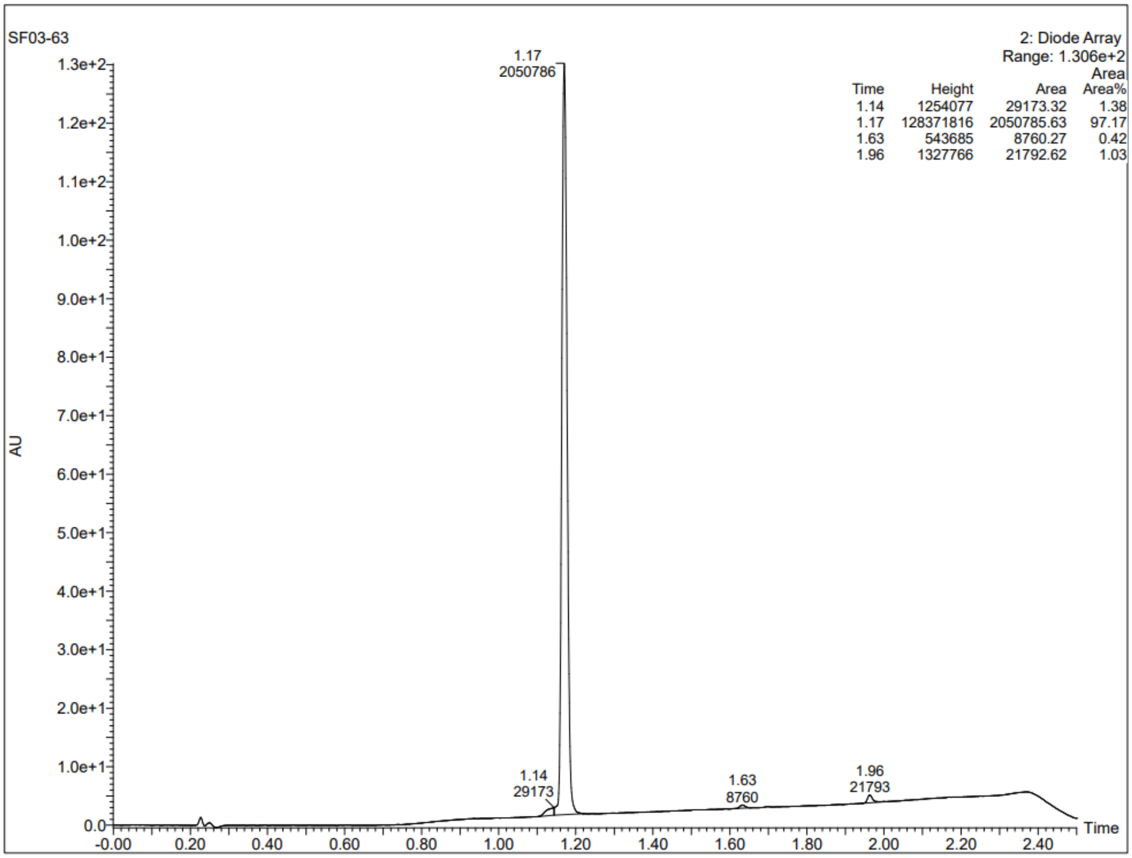

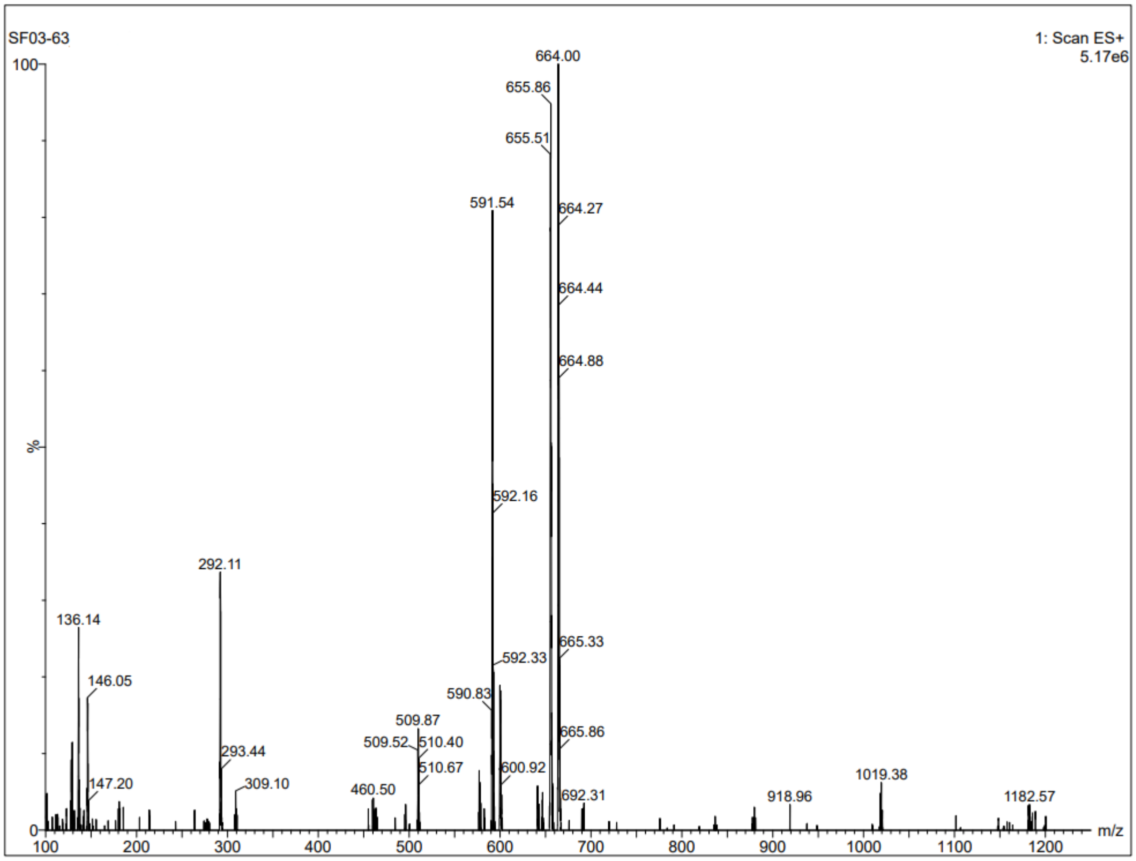

SF03-40: UPLC-MS

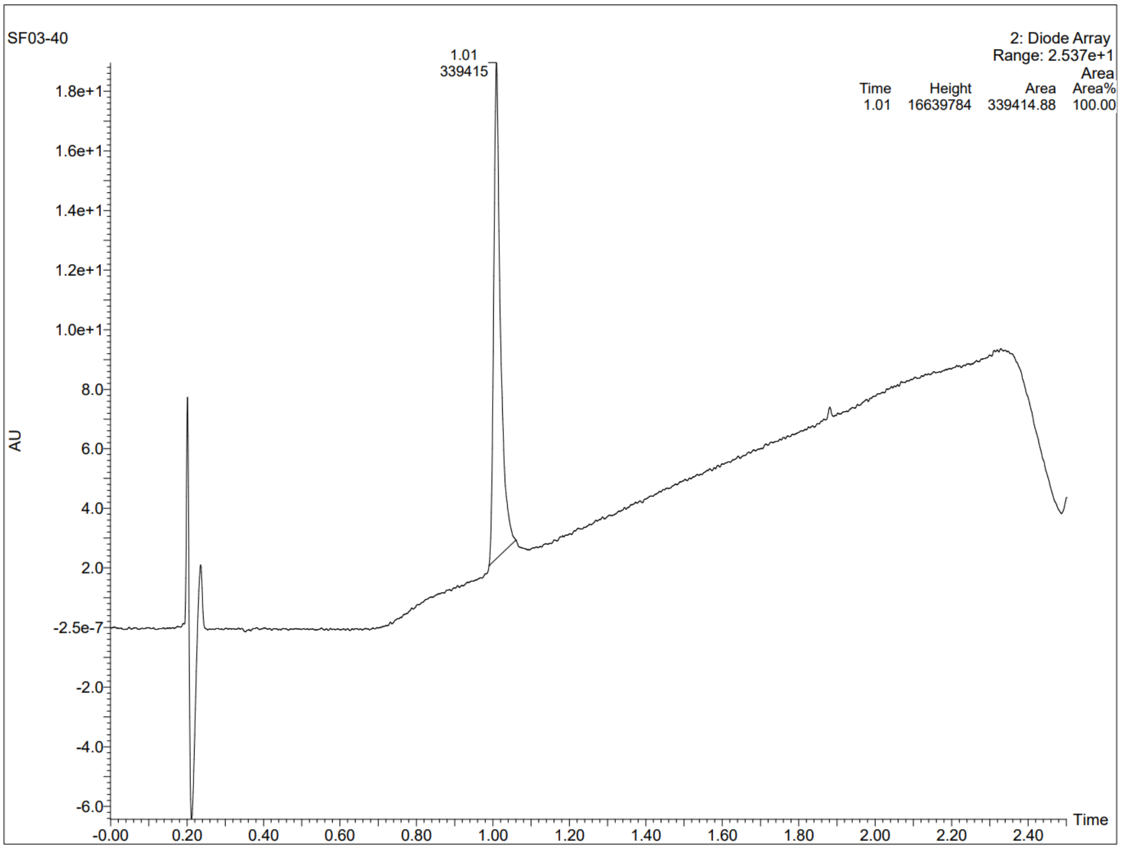

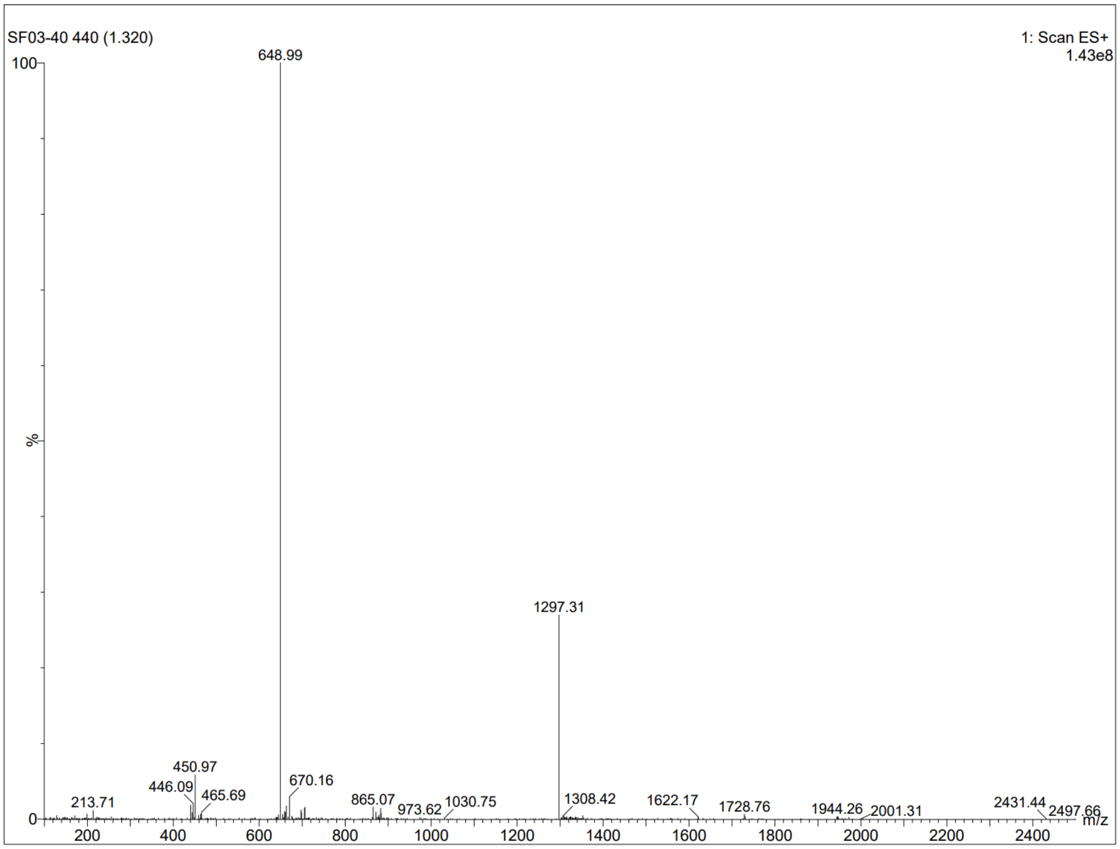

SF03-14_LIN: UPLC-MS

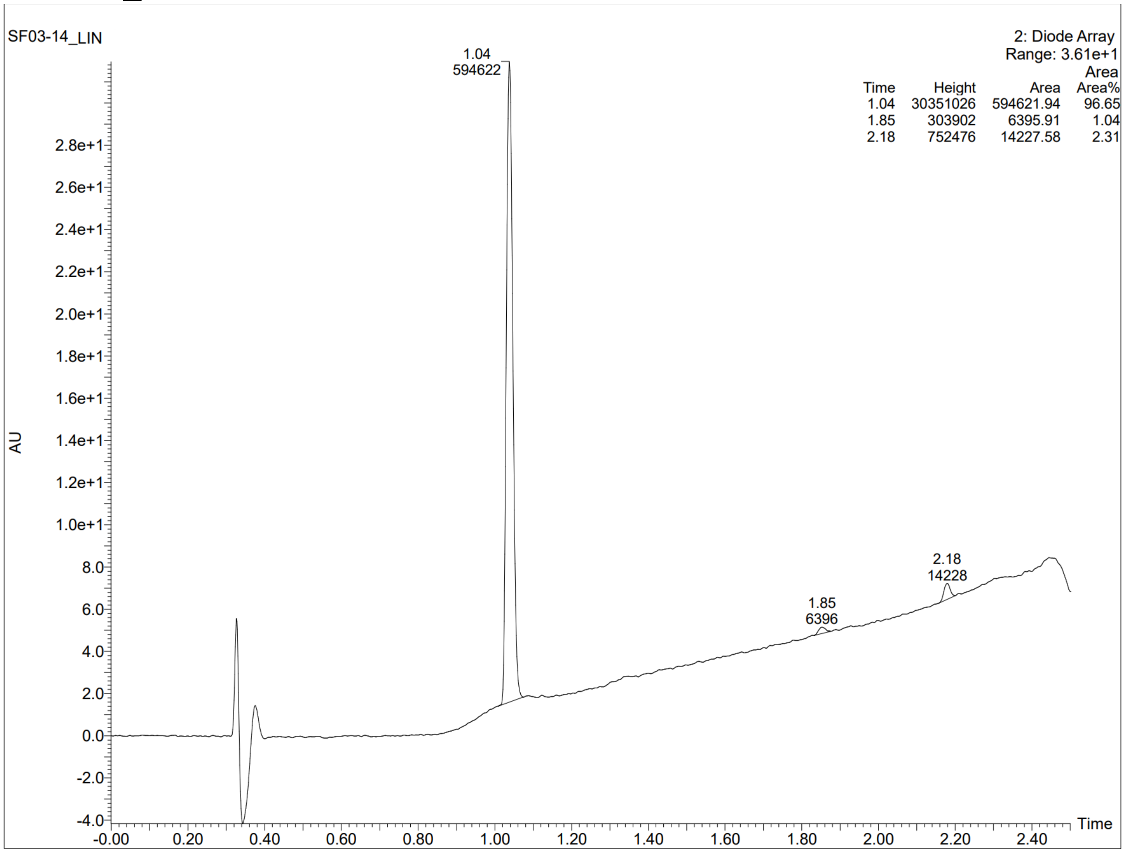

Time	Height	Area	Area Area%
1.04	30351026	594621.94	96.65
1.85	303902	6395.91	1.04
2.18	752476	14227.58	2.31

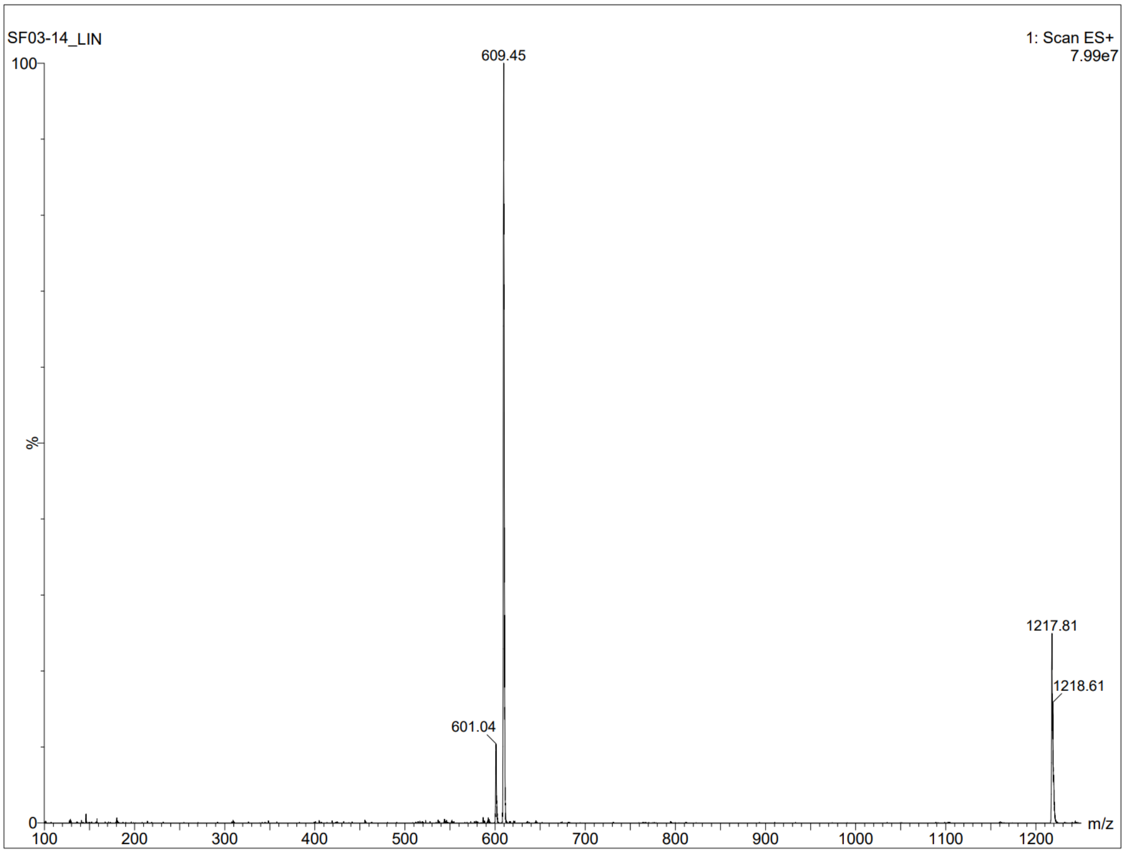

SF03-07: UPLC-MS

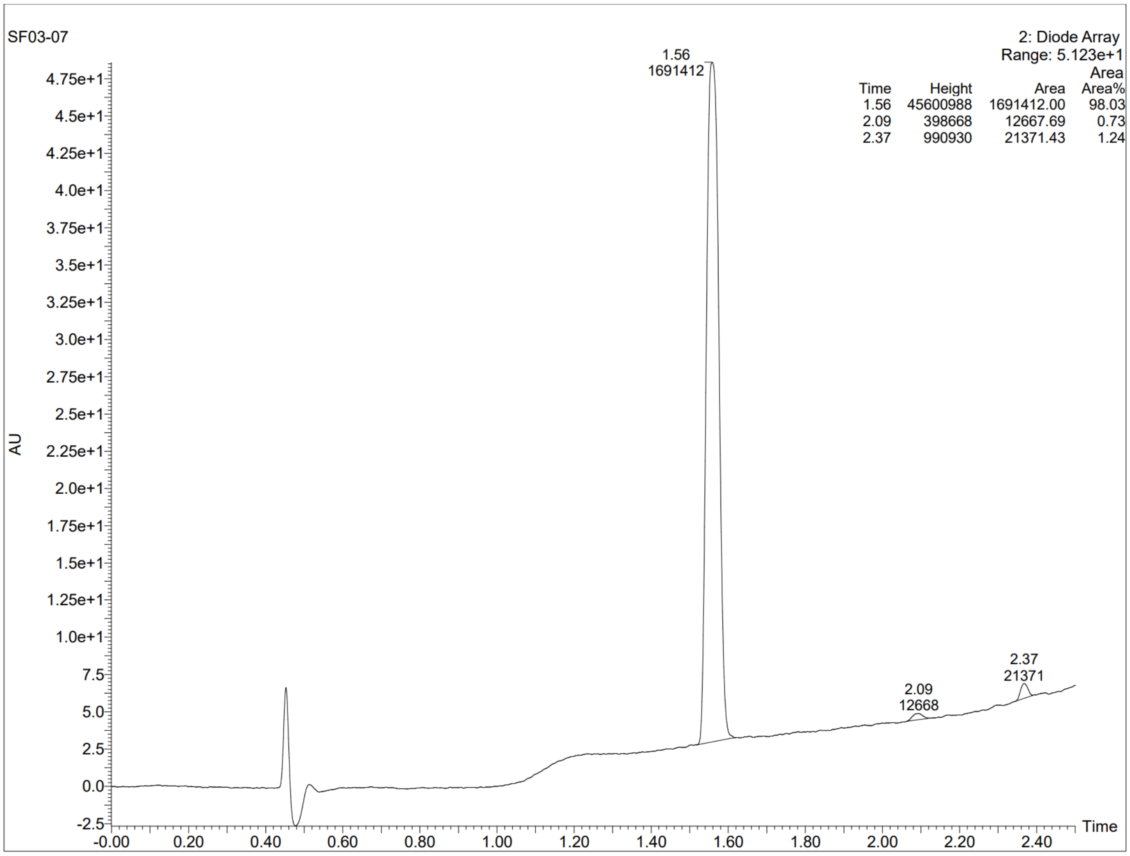

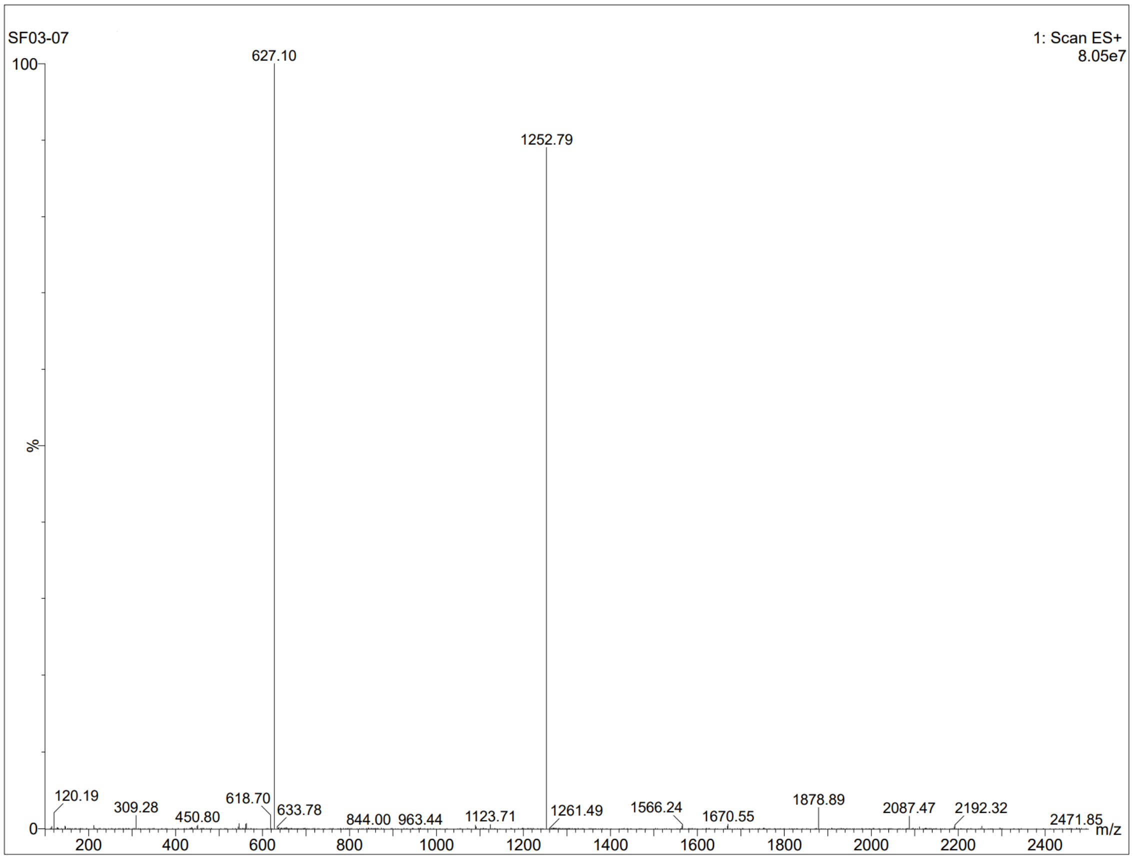

SF03-15: UPLC-MS

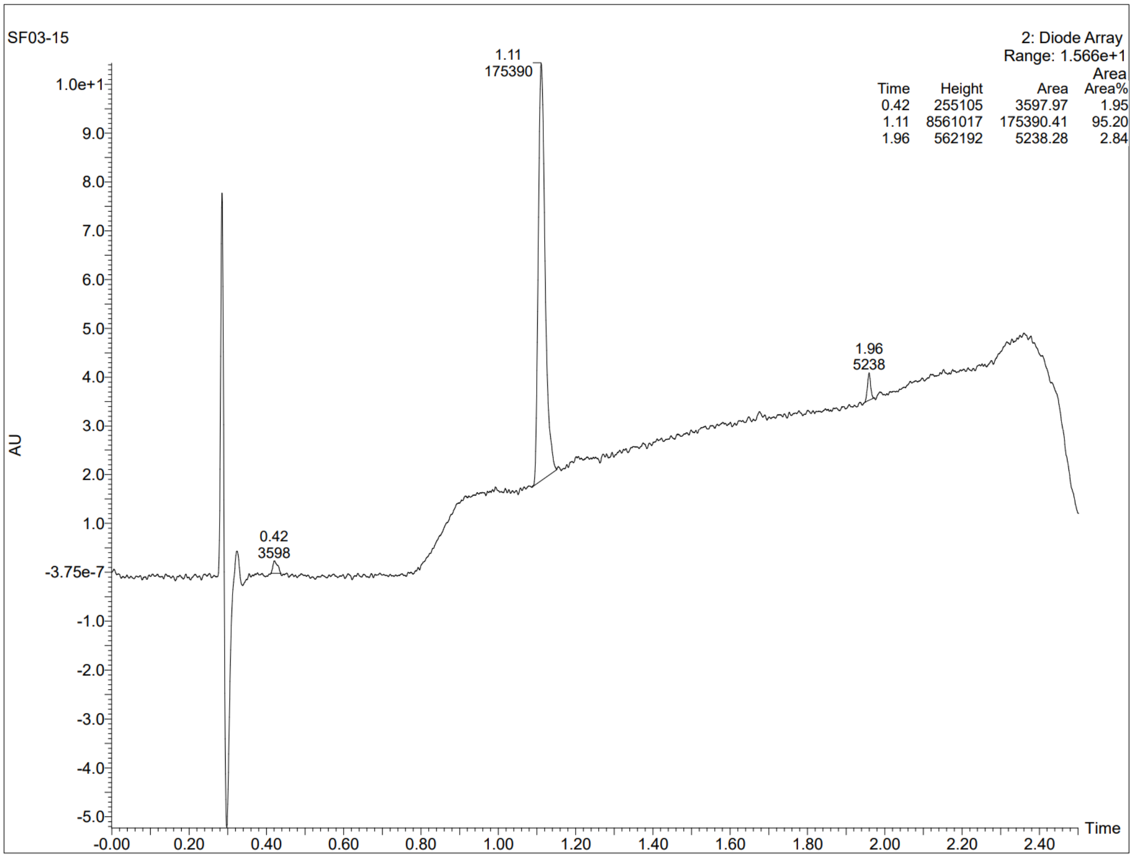

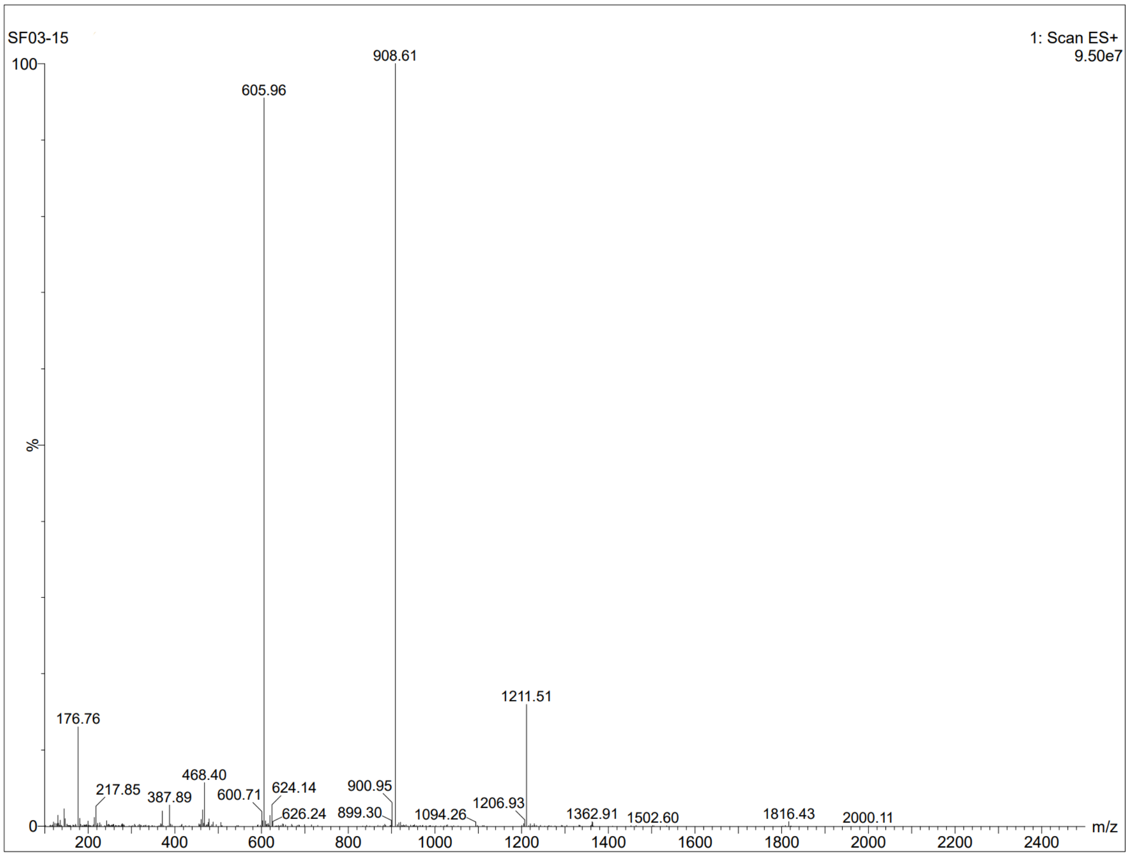

4. 1H Nuclear magnetic resonance (NMR) spectra

a. ^{1}H NMR of **4** (600 MHz, tampon phosphate, pH 6.8, 40nM KPO4, 40 KCl).

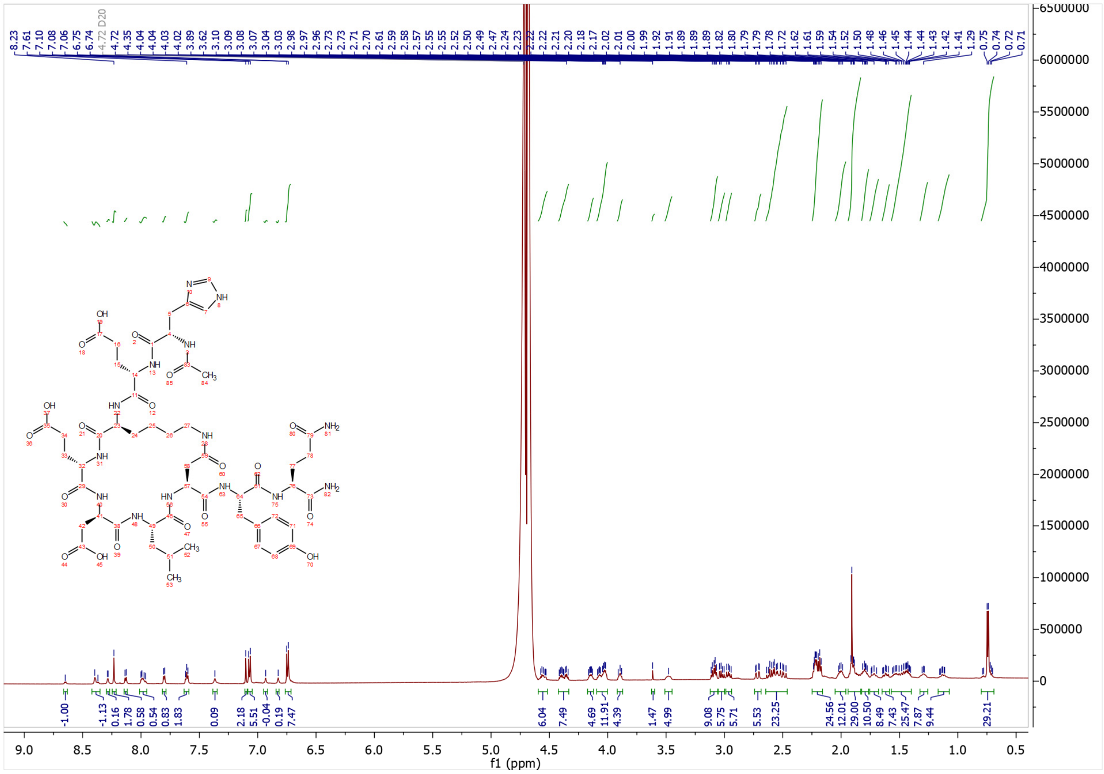

b. ^{1}H NMR of **9** (600 MHz, tampon phosphate, pH 6.8, 40nM KPO4, 40 KCl).

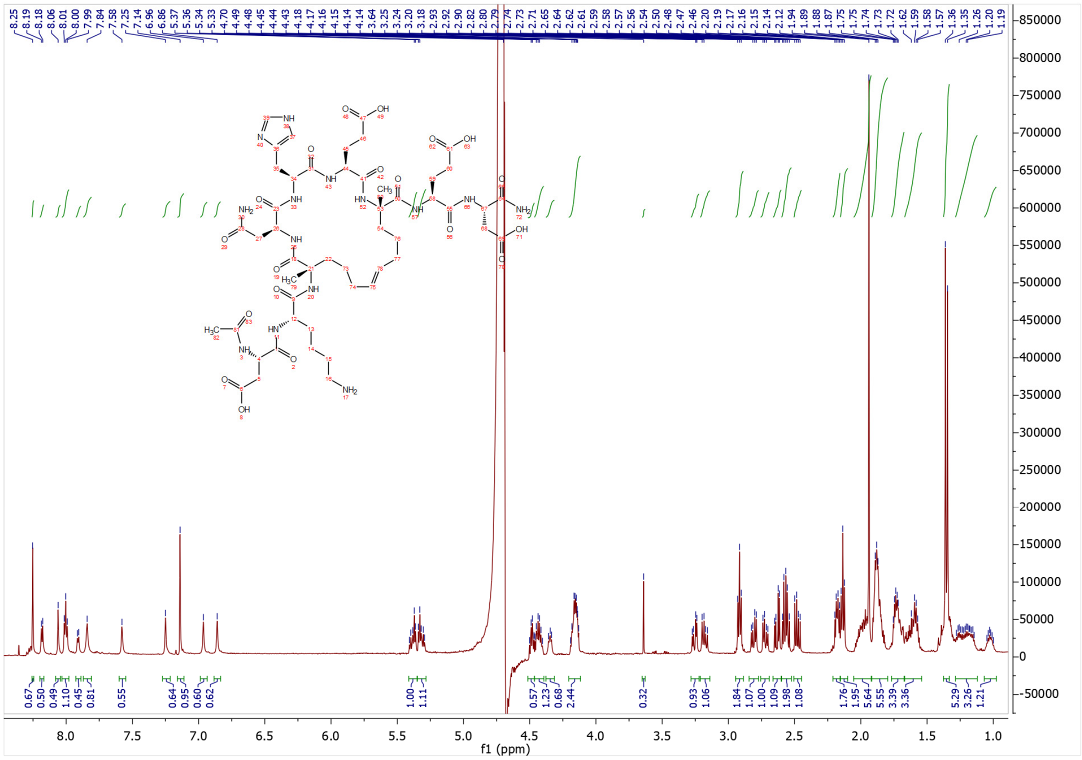

c. ^{1}H NMR of **10** (600 MHz, tampon phosphate, pH 6.8, 40nM KPO4, 40 KCl).

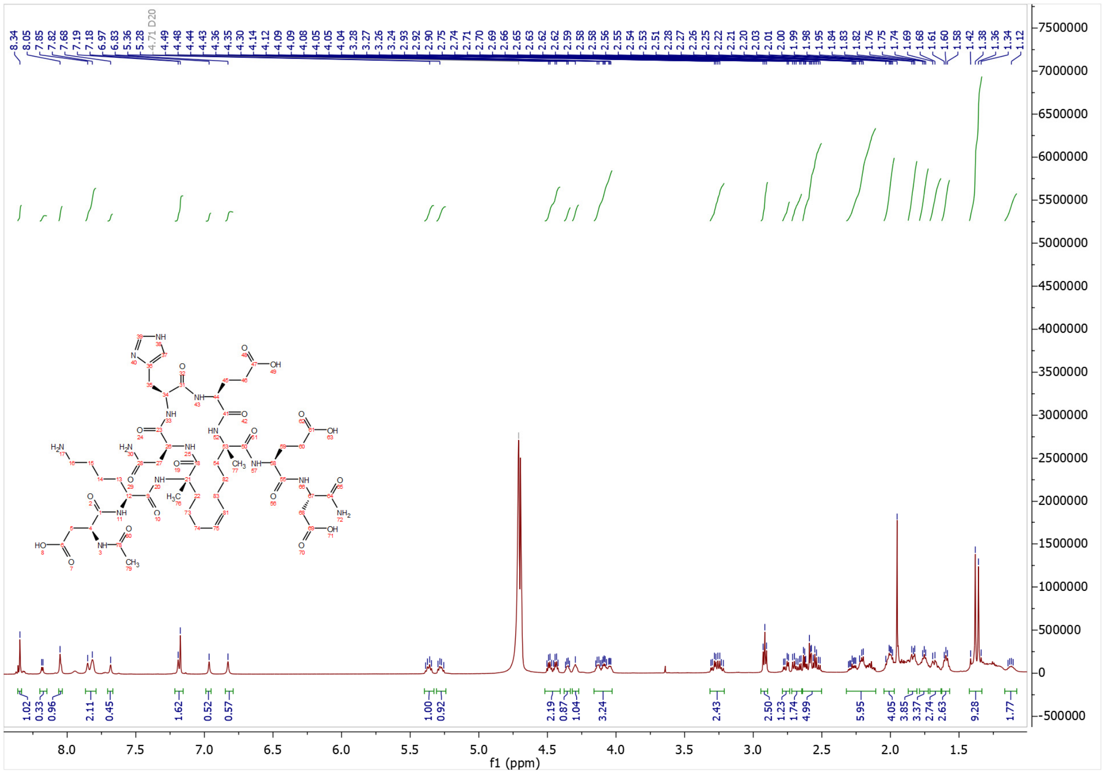

d. ^{1}H NMR of **11** (600 MHz, tampon phosphate, pH 6.8, 40nM KPO4, 40 KCl).

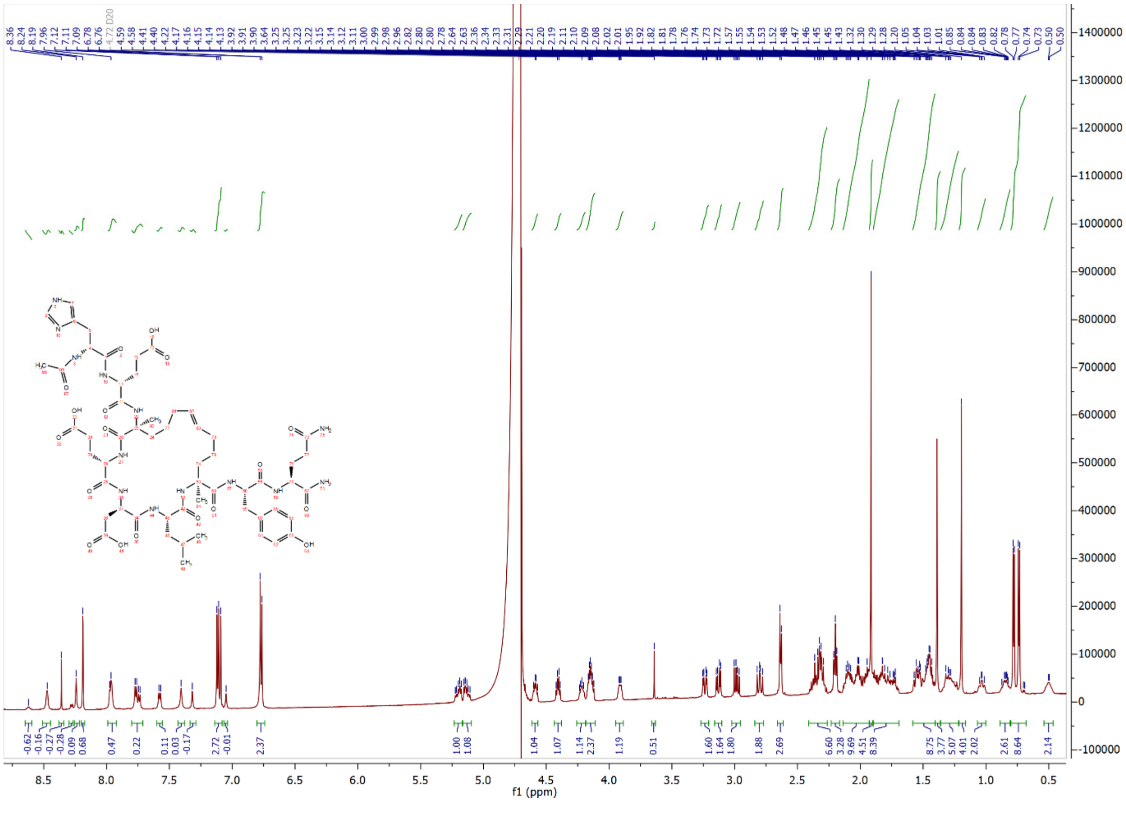

Supplement: Supplementary file 1 [file ijms-25-00166-s001.zip › ijms-2752679-supplementary.pdf]
